# Pulmonary and systemic responses to aerosolized lysate of *Staphylococcus aureus* and *Escherichia coli* in calves

**DOI:** 10.1186/s12917-020-02383-7

**Published:** 2020-05-29

**Authors:** Laura L. Bassel, Carmon Co, Alaina Macdonald, Laurel Sly, Erin E. McCandless, Joanne Hewson, Raksha Tiwari, Shayan Sharif, Laura Siracusa, Mary Ellen Clark, Jeff L. Caswell

**Affiliations:** 1 Department of Pathobiology, Ontario Veterinary College, University of Guelph, Guelph, ON N1G 2W1 Canada; 2 Global Therapeutics Research, Veterinary Medicine Research and Development, Zoetis Inc., Kalamazoo, MI USA; 3 Department of Clinical Studies, Ontario Veterinary College, University of Guelph, Guelph, Ontario Canada

**Keywords:** Cattle, Inflammation, Innate immunity, Lung, Bacteria, Bronchoalveolar lavage fluid, Diffuse alveolar damage, Pathology, Proteomics

## Abstract

**Background:**

Constitutive and inducible defenses protect the respiratory tract from bacterial infection. The objective of this study was to characterize the response to an aerosolized lysate of killed bacteria, as a basis for studying the regulation and in vivo effects of these inducible innate immune responses.

**Results:**

Bacterial lysate consisting of heat-killed and sonicated *Staphylococcus aureus* and *Escherichia coli* was aerosolized to 6 calves and systemic and pulmonary innate immune and inflammatory responses were measured in the first 24 h relative to baseline. Evaluated parameters included clinical parameters (body temperature and heart and respiratory rates), blood acute phase proteins and leukocyte counts, and leukocytes and proteins in bronchoalveolar lavage fluid. Mild clinical signs with increased heart rates and rectal temperatures developed following administration of the lysate, with resolution by 24 h. Serum haptoglobin and plasma fibrinogen concentrations were elevated at 24 h relative to baseline. Bronchoalveolar lavage fluid (BALF) had increased cellularity and increased proportion of neutrophils, as well as higher concentrations of interleukin (IL)-8, IL-10 and total protein at 24 h relative to baseline. Mass spectrometry identified 965 unique proteins in BALF: 19 proteins were increased and 26 proteins were decreased relative to baseline. The upregulated proteins included those involved in innate immunity including activation of complement, neutrophils and platelets. At postmortem examination, calves receiving higher doses of lysate had areas of lobular consolidation and interlobular edema. Histologically, neutrophils were present within bronchioles and to a lesser extent within alveoli. Calves receiving highest doses of lysate had patchy areas of neutrophils, hemorrhage and hyaline membranes within alveoli.

**Conclusions:**

Aerosolization of bacterial lysate stimulated an innate immune response in lungs and airways, with alveolar damage observed at higher doses. Such a stimulus could be of value for investigating the effects of inducible innate immune responses on occurrence of disease, or for evaluating how stress, drugs or genetics affect these dynamic responses of the respiratory tract.

## Background

The respiratory tract has not only static defences but also dynamic cellular and humoral responses to inhaled substances. For the most part, these responses protect the lungs from infection and other threats. Pattern recognition receptors on epithelial cells and leukocytes (including alveolar macrophages), as well as humoral factors such as complement proteins, act as sentinels to detect various pathogen-associated molecular patterns (PAMPs) that are unique to microbes. Once these receptors bind their ligands, signaling cascades are activated that induce the production of various mediators of inflammation and host defense, leading to rapid recruitment of neutrophils and monocytes as well as increased production of host defense proteins such as defensins. This may help eliminate pathogens but excessive inflammation can damage tissue and impair pulmonary function [1]. Thus, inflammatory responses should be inducible under circumstances where they are needed, but tightly controlled and quickly downregulated when the threat has passed.

In mice, administering PAMPs by aerosol to the respiratory tract has been shown to stimulate innate immune responses that protect against various pathogen challenges. For example, aerosolization of a lysate of nontypeable *Haemophilus influenzae* (NTHi) bacteria induced pulmonary inflammation characterized by increased concentrations of IL-6 and tumor necrosis factor (TNF)-α as well as increased neutrophils in BALF [2]. In pulmonary tissue collected 2 h following administration of NTHi, numerous genes related to host defense were upregulated including chemokines, other cytokines, pattern recognition receptors, antimicrobial peptides and oxygen radicals [3]. Similarly, administration of aerosolized Toll-like receptor (TLR) ligands to mice increased IL-6, TNF, and CXCL2 concentrations in BALF with no observed signs of illness or behavioural changes [4]. In these mice, neutrophils in BALF increased by 4 h, peaked at 48 h and returned to baseline by 7 days [4]. Aerosolization of NTHi prior to challenge with influenza A virus, *Streptococcus pneumoniae*, *Bacillus anthracis, Pseudomonas aeruginosa, Klebsiella pneumoniae, Yersinia pestis, Francisella tularensis,* or *Aspergillus fumigatus* protected against mortality [2, 3, 5]. Thus, upregulation of respiratory innate immune responses prior to pathogen challenge can be protective against bacterial and viral pathogens in mice.

Induction of innate immune defenses has also been demonstrated in cattle in response to various innate immune agonists and inflammatory cytokines. In primary cultures of bovine tracheal epithelial cells, administration of lipopolysaccharide (LPS, a TLR4 agonist), Pam3CSK4 (a TLR1/2 agonist), flagellin (a TLR5 agonist), IL-1β, TNF-α or IL-17 resulted in the induction of innate defenses such as tracheal antimicrobial peptide (TAP), lingual antimicrobial peptide (LAP), and lactoferrin [6–11]. Similarly, adenosine-5′-triphosphate, which has been shown to have antimicrobial effects [12], was released from cultured pulmonary epithelial cells in response to LPS, heat-killed bacteria and IL-1β [13]. Lipopolysaccharide induced expression of inflammatory cytokines (IL-1α, IL-1β, TNF-α, and IL-8) and the release of histamine and leukotriene B4 in cultured pulmonary epithelial cells [14, 15]. Similarly, isolated bovine alveolar macrophages increased expression of TNF-α, IL-1β and IL-8 in response to LPS [16, 17]. Following instillation of LPS into the lungs of cattle, neutrophils and macrophages were identified histologically within alveoli [18], and cell counts and the proportion of neutrophils in BALF increased, although TAP expression in bronchial mucosal biopsies did not change [8].

Thus, our hypothesis was that delivery of an aerosolized bacterial lysate to cattle would trigger innate immune responses without significant adverse effects. This was initially investigated using cell culture experiments to determine whether a lysate of killed *Escherichia coli* and *Staphylococcus aureus* upregulated TAP and LAP expression in cultured bovine tracheal epithelial cells. In vivo studies evaluated the clinical, hematologic and BALF changes and postmortem findings in calves following aerosol delivery of the bacterial lysate. The purpose was to establish a dose of bacterial lysate that induced an innate immune response without causing adverse effects, for use in further experiments. Thus, relatively low numbers of calves were used in the study, and comparisons were made between pre-treatment and post-treatment data in the same calf. We identified an inflammatory stimulus that could be tested as a novel method to prevent respiratory disease at times when immune defenses are compromised, such as in stressed beef calves at the time of arrival to feedlots. Furthermore, the use of a standardized stimulus could be useful to measure how stress or pharmacologic interventions affect the inducible responses of the respiratory tract, and to investigate the contribution of genetics or prior life events to these dynamic respiratory innate immune responses.

## Results

### Induction of TAP and LAP expression in tracheal epithelial cells

**Fig. 1:**
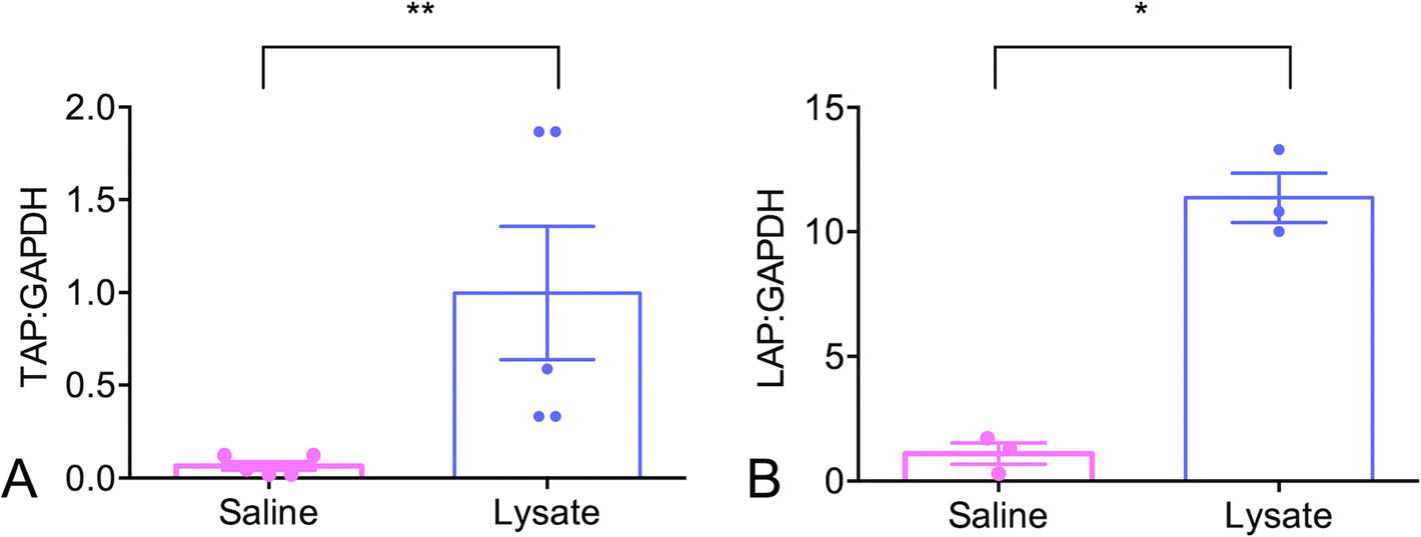
Effect of bacterial lysate on relative tracheal antimicrobial peptide (TAP) and lingual antimicrobial peptide (LAP) gene expression in tracheal epithelial cells.Primary cultures of bovine tracheal epithelial cells were exposed for 16 h to medium treated with saline (negative control) or lysate of *Staphylococcus aureus* and *Escherichia coli* (10^7^ CFU-equivalents of each). Following exposure, TAP, LAP and glyceraldehyde 3-phosphate dehydrogenase (GAPDH) gene expression were measured using RT-qPCR. Data are presented as the mean normalized TAP: GAPDH or LAP:GAPDH ratios plus or minus the standard error of the mean. Treatment with bacterial lysate resulted in an average of 15-fold greater TAP expression (*P* = 0.002; Fig. 1**a**) and 13-fold greater LAP expression (*P* = 0.018; Fig. 1**b**) compared to saline-treated cells. The data represent tracheal epithelial cells from 5 calves (TAP) and 3 calves (LAP)

Treatment of primary cultures of bovine tracheal epithelial cells with a lysate of heat-killed *E. coli* and *S. aureus* significantly increased relative TAP gene expression compared to that of cells treated with medium alone (*P =* 0.002; Fig. 1a). Similarly, relative LAP gene expression was upregulated (*P =* 0.018; Fig. 1b) in tracheal epithelial cells following administration of bacterial lysate. Treatment with bacterial lysate did not affect expression of the reference gene glyceraldehyde-3-phosphate dehydrogenase (GAPDH) based on analysis of the crossing points (*P > 0.05*).

### Clinical and clinical pathologic findings

**Fig. 2:**
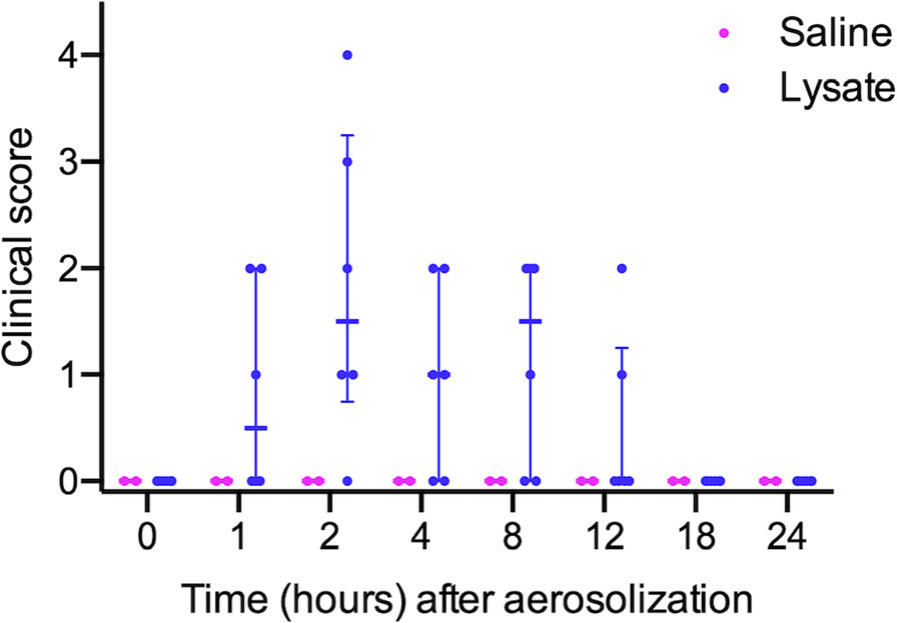
Clinical scores in conventional calves after aerosol administration of bacterial lysate. Six 3-month-old Holstein calves received aerosolized *Staphylococcus aureus* and *Escherichia coli* lysate at doses ranging from 10^8^ to 10^12^ CFU-equivalents and 2 calves received aerosolized saline (control). The graph shows individual data points, median and interquartile ranges

Six 3-month-old Holstein bull calves received aerosolized lysate of heat-killed *E. coli* and *S. aureus* (calves 1–6). All calves had clinical scores of zero prior to the administration of aerosol. Between 1 and 12 h following lysate administration, all 6 calves that received bacterial lysate developed subtle clinical signs consisting of mildly increased respiratory effort and mild depression that resolved by 18 h (Fig. 2; Additional file 1). All 6 calves coughed at one or more time points following administration of the lysate. Two calves (5 and 6) were reluctant to rise at 2 and 4 h after administration of the lysate, respectively. One calf (1) had a reduced appetite at 2 h following lysate administration. There was no association between the severity or duration of clinical signs and the dose of bacterial lysate. Two control calves (calves C1 and C2) received only aerosolized phosphate-buffered saline (PBS) to ensure that the aerosolization procedure did not affect the measured parameters; these 2 calves did not cough or show any clinical signs following aerosolization of PBS.

**Fig. 3:**
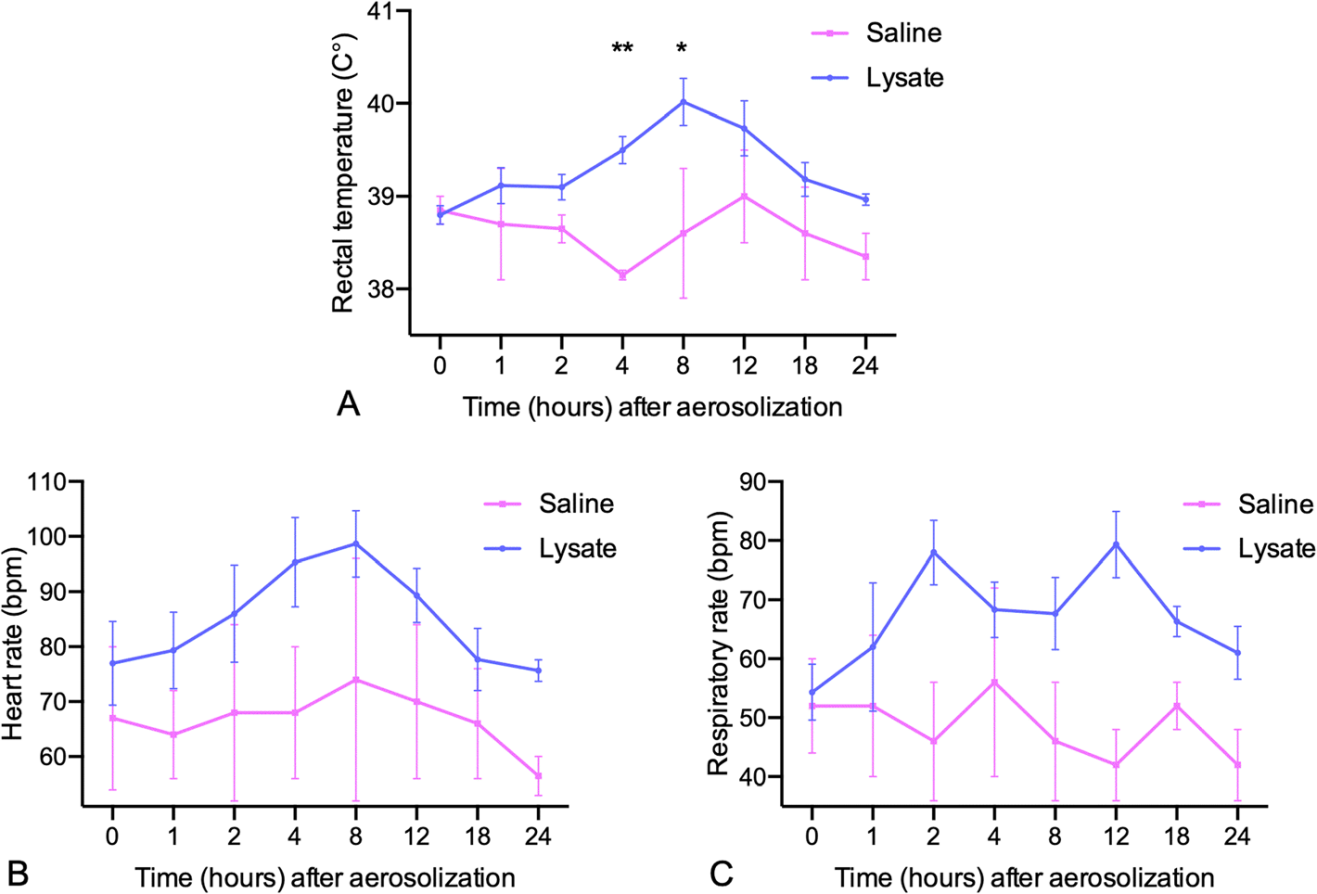
Clinical parameters in conventional calves after aerosol administration of bacterial lysate. Six 3-month-old Holstein calves received aerosolized *Staphylococcus aureus* and *Escherichia coli* lysate at doses ranging from 10^8^ to 10^12^ CFU equivalents (*n* = 6) or saline (*n* = 2). The data are mean ± standard error of the mean. **a** rectal temperature, **b** heart rate, and **c** respiratory rate over time. Individual time points were compared to baseline (time = 0) using repeated measures one-way ANOVA with Dunnett’s multiple comparison test. * *P* < 0.05, ** *P* < 0.01

Rectal temperature was increased relative to baseline in calves that received aerosolized bacterial lysate (*P =* 0.02), with significant elevations at 4 and 8 h (Fig. 3a; Additional file 1). Heart rate increased following administration of bacterial lysate (one-way analysis of variance [ANOVA]*, P* < 0.05); however, upon adjustment for multiple comparisons (Dunnett’s post hoc test), there were no significant differences in heart rate between baseline and any single post-aerosolization time point (Fig. 3b). Respiratory rate was not affected by the administration of aerosolized lysate (*P =* 0.18; Fig. 3c). In calves that received PBS, rectal temperature, heart rate, and respiratory rate did not change.

**Fig. 4:**
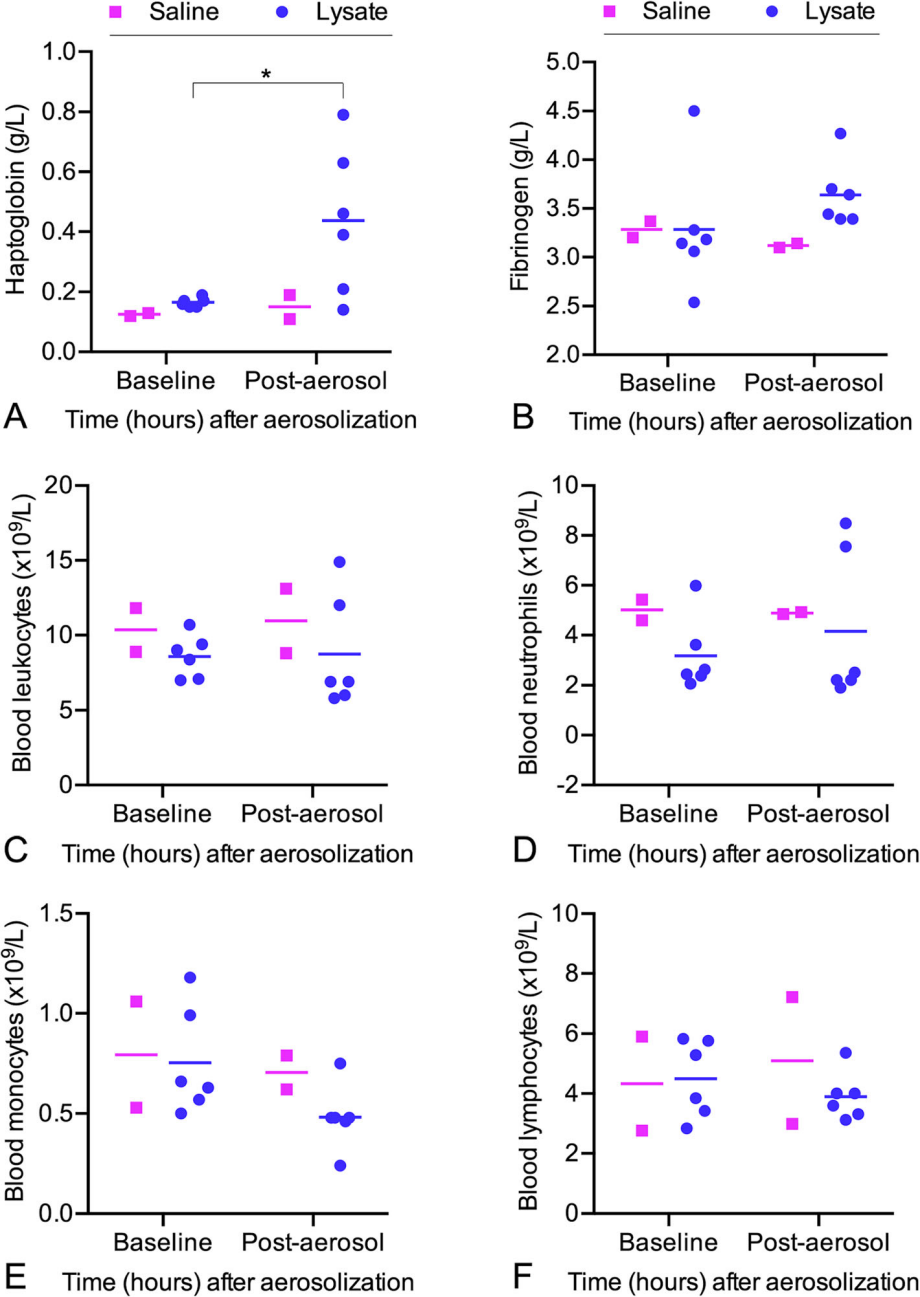
Hematologic changes in calves after aerosol administration of bacterial lysate**.** Blood samples were collected at baseline and 24 h after aerosolization of *Staphylococcus aureus* and *Escherichia coli* lysate (*n* = 6) or saline (*n* = 2). Horizontal bars indicate the mean. **a** Serum haptoglobin, **b** plasma fibrinogen, **c** total blood leukocytes, **d** blood neutrophils, **e** blood monocytes, and **f** blood lymphocytes. * *P* < 0.05

In calves treated with bacterial lysate, serum haptoglobin concentration significantly increased between baseline (mean: 0.17 g/L; 95% CI: 0.15, 0.18) and 24 h following aerosolization of lysate (mean: 0.27 g/L; 95% CI: 0.19, 0.70; paired t-test; *P* = 0.044) (Fig. 4a). Plasma fibrinogen concentration increased numerically between baseline (mean: 3.28 g/L; 95% CI: 2.60, 3.97) and 24 h following aerosolization of bacterial lysate (mean: 3.64 g/L; 95% CI = 3.29, 3.99) but the difference was not significant (*P* = 0.058; paired t-test of log-transformed values) (Fig. 4b). Administration of aerosolized bacterial lysate did not change hematologic values from baseline including total blood leukocytes (*P =* 0.92), neutrophils (*P =* 0.88)*,* monocytes (*P =* 0.08) and lymphocytes (*P =* 0.19) (Fig. 4d-f).

### Changes in bronchoalveolar lavage fluid

**Fig. 5:**
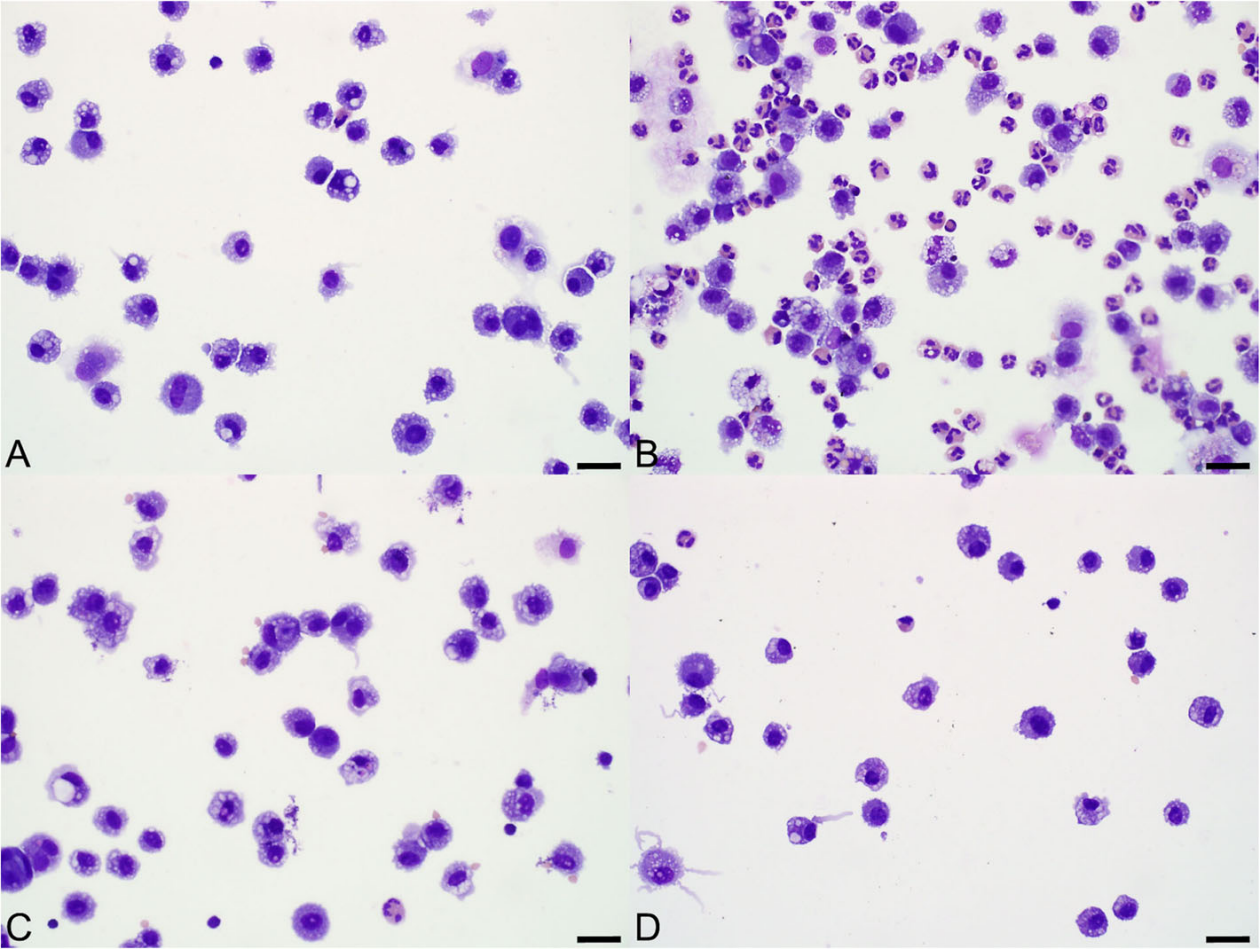
Cytology of bronchoalveolar lavage fluid from 2 calves, sampled before (**a** and **c**) and 24 h after aerosolization of either 10^12^ CFU-equivalents of *Staphylococcus aureus* and *Escherichia coli* lysate (**b**) or phosphate-buffered saline (**d**). The sample obtained after administration of lysate (**b**) contains many neutrophils whereas the other samples (**a**, **c**, **d**) contain mainly macrophages and lymphocytes. Cytocentrifuge preparation, Wright’s stain. Bar = 25 μm

**Fig. 6:**
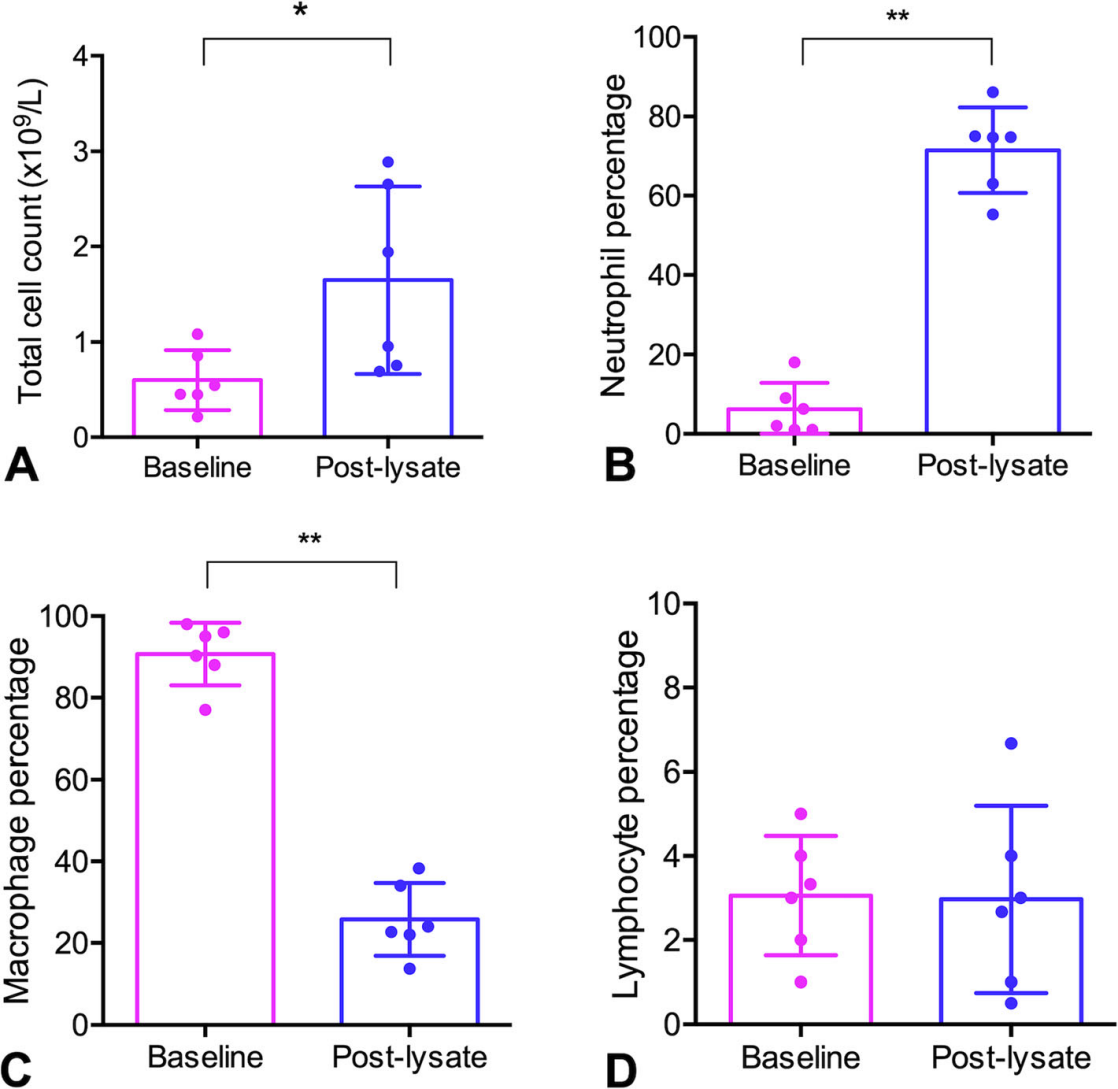
Bronchoalveolar lavage fluid cells (mean ± SEM) before and after aerosol administration of bacterial lysate to 6 Holstein bull calves. **a** Total nucleated cell count. **b** Percentage of neutrophils. **c** Percentage of macrophages. **d** Percentage of lymphocytes (y-axis scale is different than **b** and **c**). * P < 0.05, ** P < 0.01; (paired t-test)

**Fig. 7:**
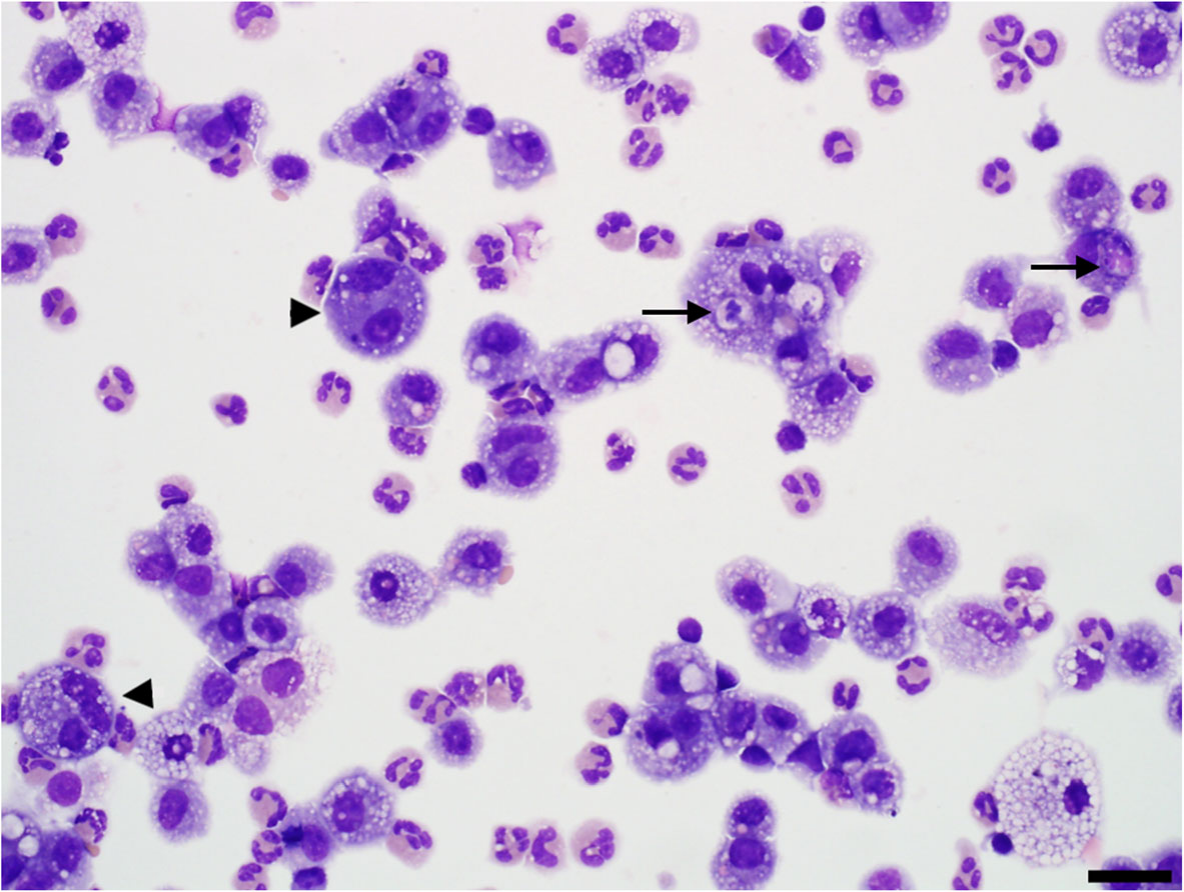
Cytocentrifuge preparation of bronchoalveolar lavage fluid from a calf that received 10^11^ CFU-equivalents of *Escherichia coli* and *Staphylococcus aureus* lysate. There are large numbers of macrophages and neutrophils. Macrophages are large, with foamy cytoplasm and frequent binucleation (arrowheads). Occasional phagocytosed neutrophils are present within the cytoplasm of macrophages (arrows). Wright stain. Bar = 25 μm

The cellular composition of BALF changed significantly following administration of bacterial lysate (Figs. 5 and 6; Additional files 2 and 3). Administration of bacterial lysate significantly increased the number of nucleated cells (*P =* 0.036*)* and the proportion of neutrophils (*P <* 0.0001) relative to baseline, while the proportion of macrophages was reduced (*P <* 0.0001) and the proportion of lymphocytes was unchanged (*P =* 0.95). Microscopically, cytocentrifuge preparations of the fluid from calves administered bacterial lysate contained large numbers of non-degenerate neutrophils as well as large macrophages with foamy cytoplasm containing occasional phagocytosed neutrophils (Fig. 7). Macrophages were significantly larger in post-lysate samples compared to baseline, based on morphometric analysis of the area of these cells in the stained cytocentrifuge preparation (*P <* 0.0001; Additional file 4).

Protein concentrations in BALF (as determined by BCA protein assay) were higher at 24 h after delivery of aerosolized bacterial lysate (mean: 122 μg/mL; 95% CI: 58, 186) compared to baseline (mean: 55 μg/mL; 95% CI: 33, 77) (*P* = 0.016, Additional file 5). In this analysis, data from one calf was excluded because the post-aerosol protein concentration was an outlier (870.8 μg/mL) as determined by Grubb’s test.

**Fig. 8:**
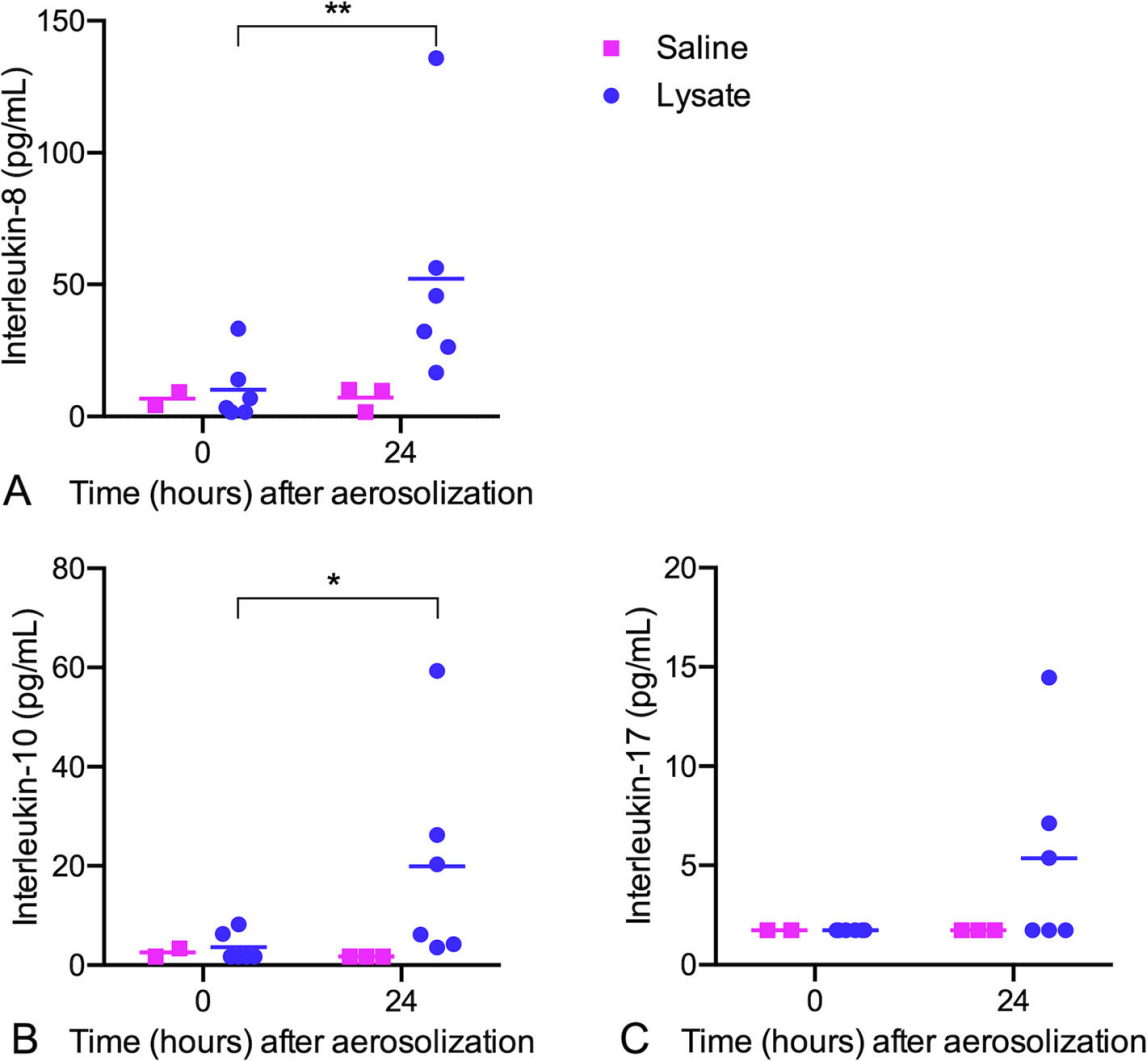
Cytokine concentrations in bronchoalveolar lavage fluid prior to and 24 h following aerosolization of *Staphylococcus aureus* and *Escherichia coli* lysate (*n* = 6) or saline (*n* = 2). Horizontal bars indicate the mean. **a** Interleukin-8. **b** Interleukin-10. **c**) Interleukin-17. * *P* < 0.05, ** *P* < 0.01; (paired t-test)

Cytokine concentrations in BALF were measured before and 24 h after aerosolization of bacterial lysate (Fig. 8). Following aerosolization, there was increased concentration of IL-8 (*P =* 0.005) and IL-10 (*P =* 0.048) compared to baseline. Following log transformation, the post-lysate concentration of IL-8 was positively correlated with the dose of bacterial lysate (R^2^ = 0.68, *P =* 0.012). Interleukin 17 concentration increased numerically following aerosolization of bacterial lysate, but this change was not statistically significant (*P =* 0.25). The following cytokines were generally below the lower limit of the assay and were not statistically analyzed: interferon (IFN) α (< 9.8 pg/mL), IL-1β (< 9.8 pg/mL), IL-2 (< 39.1 pg/mL), IL-6 (< 156.3 pg/mL), TNF-α (< 39.1 pg/mL), and IFN-γ (< 2.4 pg/mL).

BALF samples collected before and 24 h after administration of aerosolized bacterial lysate were analyzed by tandem mass tag (TMT)-labelled quantitative mass spectrometry. There were 965 different proteins identified in the BALF (Additional file 6). Individual paired t-tests identified 19 unique proteins that had increased abundance (Table 1) and 26 proteins that had reduced abundance (Table 2) following aerosolization of bacterial lysate. The 19 proteins with increased abundance in post-lysate BALF included the following protein classes (as determined by PANTHER analysis): transfer/carrier proteins (PC00219; *n* = 3), signaling molecules (PC00207; *n* = 2), receptors (PC00197; *n* = 2), defense/immunity proteins (PC00090; *n* = 2), hydrolases (PC00121; *n* = 4), and 1 of each of enzyme modulator (PC00095), oxidoreductase (PC00176), and extracellular matrix protein (PC00102) (Additional file 7). The 26 proteins that were decreased in post-lysate BALF included the following protein classes: membrane traffic proteins (PC00150; *n* = 3), hydrolase (PC00121; *n* = 3), enzyme modulator (PC00095; *n* = 3), nucleic acid binding (PC00171; *n* = 6), and cytoskeletal proteins (PC00085; *n* = 2). The remaining protein classes had only a single protein in each category (Additional file 7). Reactome analysis recognized 13 of the 19 significantly upregulated proteins and identified overrepresentation of pathways related to complement cascade, platelet activation and degranulation, neutrophil degranulation, innate immune system, and noncanonical NF-κB signaling (Additional file 8).

**Table 1:** Bronchoalveolar lavage fluid proteins that were significantly elevated following aerosolization of bacterial lysate relative to baseline

Accession	Description	Ratio of post-lysate to basline	*P*-value
**G3MYZ3**	Afamin	2.06	0.04
**Q7SIH1**	Alpha-2-macroglobulin	3.05	0.04
**P01888**	Beta-2-microglobulin	2.25	0.00
**Q0V8R6**	Beta-hexosaminidase subunit alpha	1.67	0.01
**F1N076**	Ceruloplasmin	2.92	0.04
**P81187**	Complement factor B	2.22	0.00
**Q28085**	Complement factor H	3.39	0.04
**A5PJT7**	ECM1 protein	2.78	0.02
**Q29437**	Primary amine oxidase, liver isozyme	3.23	0.01
**P82943**	Regakine-1	2.50	0.04
**Q29443**	Serotransferrin	1.92	0.01
**G3N1U4**	Serpin A3–3	2.46	0.05
**Q2KIS7**	Tetranectin	3.02	0.04
**F1MMR5**	Tetratricopeptide repeat domain 38	2.30	0.03
**Q3ZBS7**	Vitronectin	2.24	0.02
**E1BH06**	Uncharacterized protein^a^	2.46	0.05
**F1MCF8**	Uncharacterized protein	1.40	0.02
**F1MJK3**	Uncharacterized protein	4.34	0.04
**F1MVK1**	Uncharacterized protein	4.19	0.05

The table shows bronchoalveolar lavage fluid proteins that were significantly elevated (*n* = 19) following aerosolization of bacterial lysate relative to baseline, as determined by a paired t-test. Tandem mass-tag mass spectrometry was performed on bronchoalveolar lavage fluid from 4 calves obtained at baseline and 24 h after aerosol administration of bacterial lysate, with data analysis and protein normalization in Proteome Discoverer 2.2 against Uniprot Bovine Database

^a^Four uncharacterized proteins were increased following treatment

**Table 2:** Bronchoalveolar lavage fluid proteins that were significantly reduced following aerosolization of bacterial lysate relative to baseline

Accession	Description	Ratio of post-lysate to baseline	*P*-value
**A6QLI0**	Mammalian ependymin-related protein 1	0.30	0.03
**P31404**	V-type proton ATPase catalytic subunit A	0.33	0.02
**Q5E9J1**	Heterogeneous nuclear ribonucleoprotein F	0.35	0.04
**Q3T0D0**	Heterogeneous nuclear ribonucleoprotein K	0.35	0.03
**Q5E9E2**	Myosin regulatory light polypeptide 9	0.37	0.03
**G3MYX8**	Tyrosine-protein kinase receptor	0.41	< 0.001
**P04272**	Annexin A2	0.41	0.01
**Q3T0F7**	Myotrophin	0.42	0.04
**Q3T0M0**	Vacuolar protein sorting-associated protein	0.44	0.03
**Q3SZA6**	Syndecan binding protein (Syntenin)	0.52	0.02
**Q2HJH1**	Aspartyl aminopeptidase	0.54	0.05
**F1MC48**	IQ motif containing GTPase activating protein 1	0.56	< 0.001
**Q3T0S6**	60S ribosomal protein L8	0.56	0.02
**A0A140T843**	Beta-2-glycoprotein 1	0.57	0.05
**Q2YDE4**	Proteasome subunit alpha type-6	0.57	0.05
**F1MG05**	Elongation factor 1-gamma	0.58	0.02
**F1MSE7**	Sushi domain containing 2	0.60	0.05
**P11116**	Galectin-1	0.62	0.04
**Q32PH8**	Elongation factor 1-alpha 2	0.65	0.05
**F1MM32**	Sulfhydryl oxidase	0.68	0.01
**G3MXB5**	Uncharacterized protein	0.69	0.00
**A6QLG5**	40S ribosomal protein S9	0.69	0.01
**Q0IIM3**	Heat shock protein 105 kDa	0.71	0.05
**Q3ZC44**	Heterogeneous nuclear ribonucleoprotein A/B	0.72	0.03
**Q1RMR9**	Protein kinase C and casein kinase substrate in neurons 2	0.72	0.03
**A5PKK0**	FAM151B protein	0.81	0.05

### Pathologic findings

**Fig. 9:**
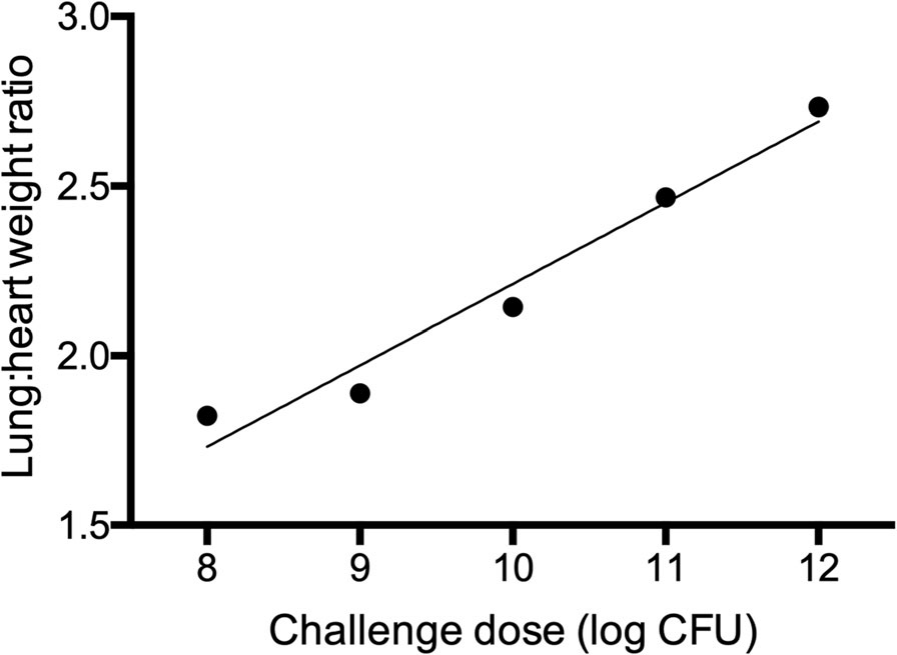
Relationship of challenge dose of *Staphylococcus aureus* and *Escherichia coli* lysate with lung: heart weight ratios. The data show the ratio of lung and heart weights measured at the time of postmortem examination, 24 h after 4 calves were administered 10^8^, 10^9^, 10^10^ or 10^11^ CFU-equivalents of *Staphylococcus aureus* and *Escherichia coli* lysate by aerosol. The ratio of lung to heart weight was correlated with the dose of bacterial lysate (R^2^ = 0.96, *P* = 0.003)

At postmortem examination, the calculated lung to heart weight ratio was positively associated with the dose of aerosolized bacterial lysate (R^2^ = 0.96, *P =* 0.003; Fig. 9). Grossly, lesions were predominately in the cranioventral areas of lung and included red mottling of the lung tissue, interlobular edema, and scattered discrete angular red-purple foci of lobular consolidation (Additional file 9). In the calves that received 10^12^ colony forming unit (CFU)-equivalents of bacterial lysate, lungs were mottled red with mild interlobular edema. The calves that received 10^10^ and 10^11^ CFU-equivalents had small, multifocal areas of lobular consolidation in the cranioventral lung lobes, affecting less than 5% of the total lung parenchyma. The calf that received 10^8^ CFU-equivalents had no gross lesions. The calf that received 10^9^ CFU-equivalents had an area of consolidation of a cranial lung lobe that was determined by histopathology to have been present for greater than 3 days prior to euthanasia (that is, preceding aerosolization of bacterial lysate).

**Fig. 10:**
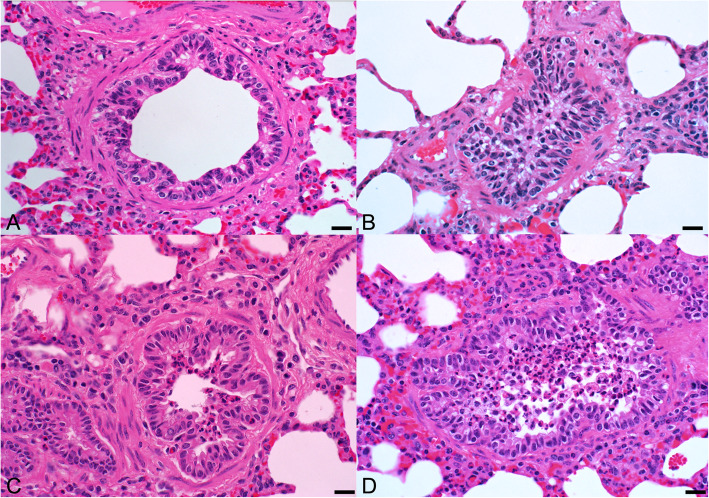
Histologic sections of bronchioles from calves that received aerosolized bacterial lysate 24 h prior to euthanasia. At lower doses of bacterial lysate (**a**, calf 1, 10^8^ CFU-equivalents; and **b**, calf 2, 10^9^ CFU equivalents), the bronchioles have no or few neutrophils within their lumens. At higher doses of bacterial lysate (**c**, calf 3, 10^10^ CFU-equivalents; and **d**, calf 4, 10^11^ CFU-equivalents) there are neutrophils within bronchioles. Bar = 40 μm

**Fig. 11:**
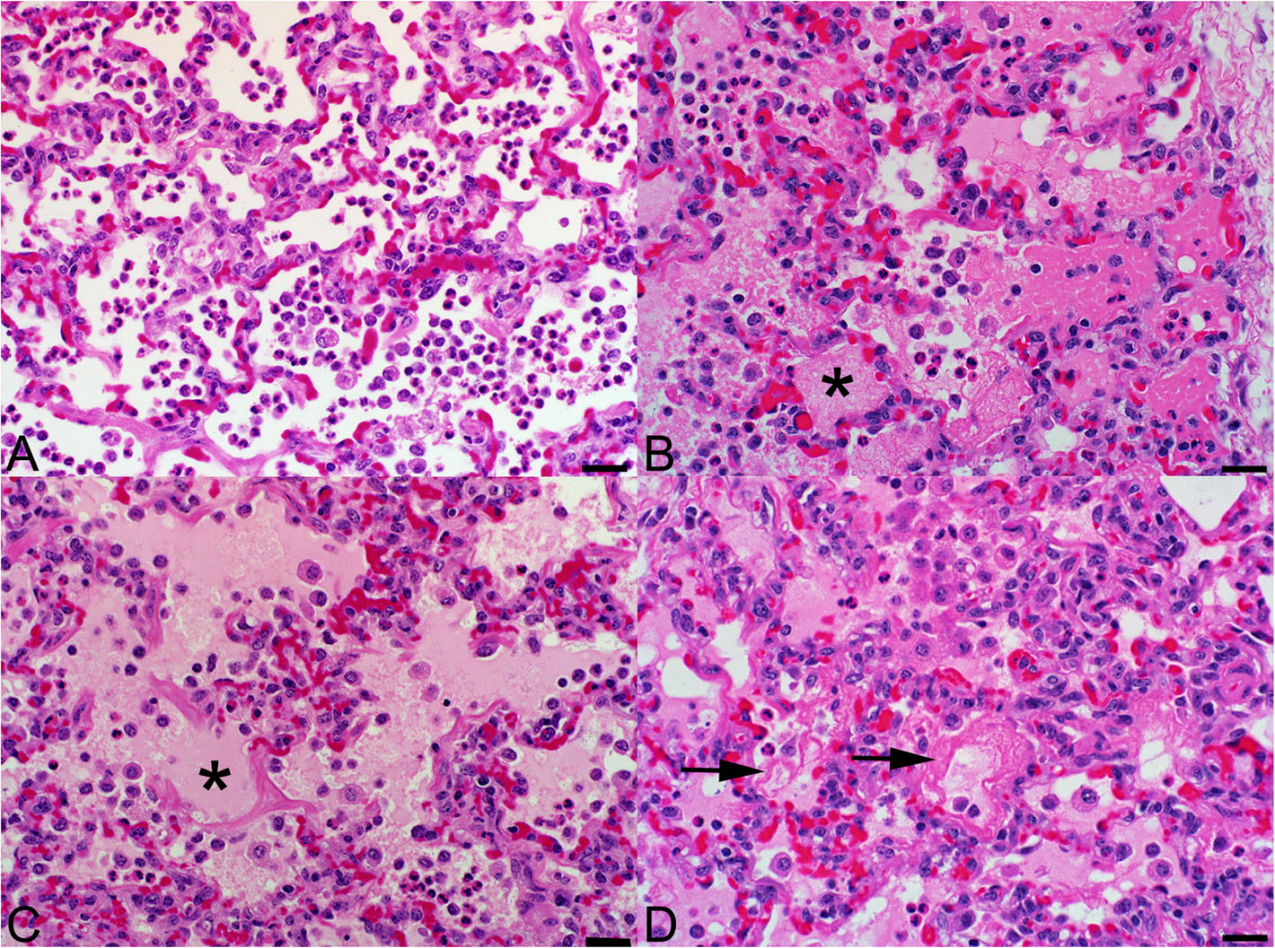
Histologic sections of lungs from calves receiving 10^10^ (**a**), 10^11^ (**b**), 10^12^ (**c**, **d**) and CFU-equivalents of *Escherichia coli* and *Staphylococcus aureus* lysate. There are neutrophils and macrophages in alveoli (**a**), fibrin (**b**) or eosinophilic fluid (**c**) in alveoli (asterisks), thickened hypercellular alveolar septa (**c**), and hyaline membranes (**d**, arrows) indicating diffuse alveolar damage

Histologically, bronchioles and to a lesser extent alveoli contained neutrophils in airspaces and occasionally in the bronchiolar lamina propria and alveolar septa. Small numbers of neutrophils were visible in bronchiolar lumens and rarely in alveoli of the calves that received the lower doses of bacterial lysate (10^8^ and 10^9^ CFU-equivalents). In the calves that received the higher doses (10^10^ to 10^12^ CFU-equivalents), neutrophils were more numerous in bronchiolar lumens and alveoli (Fig. 10; Additional file 10). In the calves that received the highest doses (10^10^ to 10^12^ CFU-equivalents), there were areas of acute alveolar damage evidenced by hyaline membranes and intra-alveolar neutrophils, macrophages and brightly eosinophilic (protein-rich) edema fluid (Fig. 11). The histologic lung lesions were ranked, based on severity of inflammation by 2 independent observers, without knowledge of the treatment status. The severity of inflammatory lesions in the lung corresponded to increasing doses of bacterial lysate.

## Discussion

The pulmonary innate immune system is critical for detecting and responding to inhaled pathogens, yet there has been relatively little investigation into ways to stimulate or increase the effectiveness of these responses. In situations where a pathogen reaches the lung, the course of disease depends on many factors including host immunity and pathogen virulence. There is considerable variation among individuals in both the magnitude of inducible innate immune defenses [19, 20] and the severity of disease following bacterial pathogen challenge [21]. Thus, administration of aerosolized bacterial lysate in the current study could provide a standardized stimulus to investigate the genetic or acquired basis for this variability.

Evaluating the response to bacterial challenge is complicated by survival or host-mediated killing of the bacteria, and by the effects of bacterial virulence factors that may modulate the host innate immune response. By using killed bacterial lysate, we were able to characterize pulmonary and systemic responses to an inflammatory stimulus delivered to the lung, independent of pathogen survival. Gaining an understanding of these responses to bacterial lysate can allow us to compare innate immune responsiveness between individuals, examine the effects of various external influences, and investigate the impacts of immunomodulatory drugs on these responses.

Initial in vitro experiments confirmed that bacterial lysate upregulated the host defense peptides LAP and TAP in cultured tracheal epithelial cells. This is consistent with previous studies that demonstrated upregulation of TAP and LAP in response to the purified TLR agonists LPS and Pam3CSK4 [8, 10, 11]. Similarly, administration of non-typeable *Haemophilus influenzae* lysate to cultured murine epithelial cells resulted in upregulation of 18 of the 30 antimicrobial peptides examined and enhanced bacterial killing [3]. As the focus of this investigation was on the in vivo effects of aerosolization of bacterial lysate, we did not investigate additional genes in cultured epithelial cells or the in vitro responses of other cell types.

We used a bacterial lysate rather than purified agonists to stimulate innate immune responses. It has been shown that combinations of agonists can induce synergistic or complementary innate immune responses [22, 23]. In the bovine lung, it is likely that the simultaneous stimulation of multiple pattern recognition receptors by lysed bacteria, similar to that in natural pathogen exposure, could similarly induce a more robust response than individual purified agonists, although this was not specifically investigated in these experiments.

The aerosol treatments were well-tolerated by the calves. Although mild clinical signs including elevated respiratory effort, cough, and nasal discharge developed shortly after aerosolization of bacterial lysate, these resolved by 24 h. Similarly, in mice that received a sub-lethal dose of *Pseudomonas aeruginosa*, lung function and rectal temperature were most significantly decreased and behavioural signs of illness were highest between 6 and 12 h post-inoculation [24].

Neutrophils were markedly increased in BALF following aerosolization of bacterial lysate. BALF was sampled from caudodorsal areas of lungs to minimize any effects of pre-existing lung inflammation. As gross and histologic lesions were more numerous in the cranioventral than caudodorsal lung lobes, the analysis of BALF from the caudodorsal region may have underestimated the magnitude of the response to the aerosolized bacterial lysate. Experiments in mice demonstrated that neutrophils appeared in lung lavage fluid at 4 h after treatment with the TLR agonists oligodeoxynucleotide and Pam2CSK4 [4]. In that study, the number of neutrophils in BALF peaked at 48 h and returned to baseline by 7 days. In the current study, there was no apparent relationship between the dose of lysate and the magnitude of the increase in neutrophils in BALF. This suggests that each of the tested lysate doses were above the threshold for a maximal response. In support of this, neutrophil recruitment in response to oligodeoxynucleotide and Pam2CSK4 in mice is reported to follow a sigmoidal dose-response curve, with a plateau at higher doses [4]. In cattle, aerosolization of *Mannheimia* (*Pasteurella) haemolytica* significantly increased the neutrophil:macrophage ratio in BALF, beginning at 30 min and increasing up to 4 h after challenge [25, 26]. Conversely, although inhalation of endotoxin from *Salmonella typhimurium* induced increases in the percentage of neutrophils within the BALF, inhalation of *Staphylococcus epidermidis* did not alter the cell populations [25]. This is consistent with a pilot experiment we conducted (data not shown) where aerosolization of heat-killed *S. aureus* did not induce clinical effects or significantly increased neutrophil numbers in BALF. Thus, the inclusion of *E. coli* lysate augmented the pulmonary inflammatory response compared to *S. aureus* alone. Similarly, killed Gram-negative bacteria were found to contribute to robust induction of inflammatory responses [25]. In the present study, at 24 h after administering bacterial lysate, macrophages in BALF demonstrated evidence of activation with a larger cell size than at baseline, vacuolated cytoplasm, and frequent binucleation. The phagocytosis of apoptotic neutrophils (efferocytosis) observed in these calves is thought to be important in the resolution of inflammation [27].

The most consistent histologic observations were neutrophils within bronchiolar walls and lumens, whereas neutrophils were more variably observed within alveoli. Neutrophils were more numerous in calves that received higher doses of bacterial lysate. The dearth of neutrophils in baseline BALF and their abundance after stimulation confirmed that neutrophil infiltration occurred after and in response to the bacterial lysate. Neutrophils, lymphocytes and plasma cells were also present within bronchial and tracheal mucosa but these can be present in conventionally reared calves [28].

At higher doses, the aerosolized bacterial lysate induced histologically evident lung injury, including intra-alveolar neutrophils, hyaline membranes, and hypercellularity of alveolar septa. In addition, the gross lung:heart weight ratios increased with increasing dose, presumably due to edema as a result of increased vascular permeability. However, the presence of edema did not affect respiratory rates at the time of euthanasia. In mice, lung weights were also increased following aerosolization of oligodeoxynucleotide and Pam2CSK4 [4]. Aerosolization of LPS has been used to model septic lung injury, similar to what was seen in the calves receiving a high dose of lysate [29–31]. Based on these findings, doses of 10^9^ to 10^10^ CFU-equivalents of bacterial lysate were chosen for future studies.

Aerosolization of bacterial lysate in the present study significantly increased BALF protein concentration and inflammatory cytokines, consistent with other studies that delivered aerosolized lysates or innate immune agonists [30]. Interleukin 8 and IL-10 concentrations were elevated in BALF after stimulation. Interleukin 8 has been shown to recruit neutrophils to the bovine lung [32]. Interleukin 10 functions to downregulate inflammatory responses and inhibits release of pro-inflammatory cytokines from macrophages and other leukocytes; its induced expression in response to bacterial infection or inflammatory cytokines is thought to represent a feedback loop to protect against excessive inflammation and tissue damage [33]. Other cytokines were below the limit of quantification (TNF-α, IFN-α, IL-1β, IL-2, IL-6). This may reflect the fact that BALF was sampled from caudodorsal areas of lung whereas lesions were more severe in cranioventral areas. Further, this may be due to the timing of the sample collection, as IL-1β and TNF-α were shown to peak and return to baseline within 8 h of exposure to *M. haemolytica* [34]. Mouse studies found that the cytokines IL-6, CCL2 and TNF peaked within 8 h and returned almost to normal following aerosol administration of CpG oligodeoxynucleotide and Pam2CSK4 [4]. Similarly, following non-lethal administration of *Pseudomonas aeruginosa*, TNF-α levels peaked at 4 h and returned to baseline by 24 h [24]. It is therefore possible that changes in cytokine concentrations occurred at an earlier time point.

Mass spectrometry identified numerous proteins within bovine BALF, and there were significant increases in the levels of several proteins related to innate immunity and the acute phase response, including complement components, metal-scavenging proteins, protease inhibitors, and chemokines. Many of the upregulated proteins identified by our analysis were involved in innate immune pathways including complement activation and neutrophil degranulation. Activation of neutrophil-related pathways corresponds to the increased number of these cells in bronchoalveolar lavage fluid and in histological sections. The overrepresentation of pathways related to platelet activation and degranulation are consistent with their increasingly recognized role in pulmonary innate immunity. It should be noted that this analysis does not determine the cellular source of the detected proteins, nor whether the increased levels result from greater synthesis of the protein, secretion from infiltrating cells such as leukocytes, exudation of proteins from plasma, or reduced degradation or clearance.

These experiments investigated a range of concentrations of bacterial lysate from 10^8^ to 10^12^ CFU-equivalents. The lung: heart weight ratios, concentrations of IL-8 in BALF, and histologic lesions of inflammation and alveolar damage were greater in calves receiving the higher doses of bacterial lysate. We did not determine a minimum dose to elicit pulmonary neutrophil recruitment, and it is possible that all tested doses were above a response threshold.

## Conclusions

In vitro studies demonstrated that the bacterial lysate induced gene expression of host defense peptides. In vivo aerosol administration of bacterial lysate stimulated a transient systemic inflammatory response with short-term elevations in rectal temperature and heart rate, mild increases in blood biomarkers of inflammation, but no differences in blood leukocytes. Between baseline and 24 h after administering lysate, the BALF had a significant increase in neutrophil numbers, enlargement of macrophages with foamy cytoplasm, and increased concentrations of total protein, IL-8 and IL-10. Mass spectrometric analysis of BALF proteins identified 965 proteins, with altered levels of several proteins related to complement activation, neutrophils and platelets after administration of the lysate. Understanding and characterizing the pulmonary inflammatory responses could provide a platform to investigate innate immune responses, measure the effect of host or environmental influences on these responses, and evaluate immunomodulatory therapeutics. Further studies will investigate the effects of this immune stimulant on development of respiratory disease.

## Methods

### Preparation of bacterial lysate

An isolate of *Staphylococcus aureus* from a case of bovine mastitis and an isolate of *Escherichia coli* from a case of bovine diarrhea were kindly provided by the Animal Health Laboratory (AHL), University of Guelph. The *E. coli* isolate was considered enterotoxigenic based on identification of F41, F5/K99 and STa genes but was not enteropathogenic or verotoxigenic based on absence of eaeA, hlyA, or Shiga toxins 1 or 2 genes). A stock suspension of each isolate was streaked onto a blood agar plate and cultured at 37 °C for 24 h. A single colony of *Staphylococcus aureus* and of *E. coli* were each inoculated into separate flasks containing 250 mL tryptose soy broth and incubated at 37 °C with shaking for 16 h (stationary phase). The concentration of viable bacteria (colony forming units (CFU)/mL) was determined from aliquots of the bacterial preparations, by plating serial dilutions onto Columbia agar with 5% sheep blood (Oxoid Canada, Nepean, Ontario, Canada), incubating overnight at 37 °C, and counting the number of bacterial colonies. Other aliquots of the bacterial preparations were killed by incubation in a 65 °C water bath for 90 min. Bacterial killing was confirmed by lack of growth on blood agar. The suspension was centrifuged at 4 °C at 10000 x *g* for 15 min and washed twice with cold PBS. The bacterial pellets were re-suspended in PBS to create a final stock solution containing 1 × 10^11^ CFU-equivalents/mL. The heat-killed bacteria were sonicated using a dismembranator (Model 120, Fisher Scientific) at 90% amplitude alternating 20 s pulses with 30 s rest for a total of 5 min. Aliquots of bacterial lysates were stored at − 20 °C. Concentrations of the bacterial lysate are reported as CFU-equivalents.

### Cell culture experiments

Primary cultures of bovine tracheal epithelial cells were established as previously described [11, 20]. Once the cells reached 80% confluency, triplicate wells were treated with medium only (negative control) or a combination of 10^7^ CFU-equivalents each of *S. aureus and E. coli* lysate. Cells treated with 0.1 μg/mL LPS (Sigma Aldrich, MO, USA, L9143) were used as a calibrator. Following treatment, the cells were incubated for 16 h before being harvested for RNA extraction.

RNA extraction and cDNA synthesis were carried out as previously described [20]. Real-time reverse transcription quantitative PCR was used to evaluate the effect of bacterial lysate on TAP and LAP expression relative to expression of the reference gene GAPDH as previously described [10, 11, 20]. Stability of expression of GAPDH across treatments was assessed using ANOVA. Template-negative wells were included in each run, and data of technical triplicate samples were averaged. The specificity of the reaction was confirmed by examining melting curves and identification of a single peak at approximately 82.8 °C (TAP), 86.9 °C (LAP) and 85.4 °C (GAPDH).

### Aerosolization of bacterial lysate to calves

Use of animals in these studies was approved by the Animal Care Committee of the University of Guelph (AUP #3286) according to Canadian Council on Animal Care guidelines. Calves were obtained from the Elora Dairy Research Centre (Ontario Ministry of Agriculture, Food and Rural Affairs). Eight 3-month-old Holstein bull calves were housed in pairs within climate-controlled, biosecurity level 2 pens in an isolation facility. Shavings were used as bedding, and hay, water and calf starter pellets (Sharpe Farms Supplies Limited, Guelph, Ontario) were offered *ad libidum*.

An aerosol of *S. aureus* and *E. coli* lysate was administered to 6 calves. Calves were randomly assigned to receive either 10^8^ (calf 1), 10^9^ (calf 2), 10^10^ (calf 3), 10^11^ (calf 4) or 10^12^ (calves 5 and 6) CFU-equivalents suspended in a total volume of 10 mL PBS. The bacterial lysates or PBS were delivered by aerosol using a compressor (Precision Medical, Northampton, PA) that produced approximately 25 psi of air pressure and was attached to a Whisper Jet nebulizer (Marquest, Englewood, Colo) connected to a small Equine Aeromask (Trudell Medical International, London, ON) placed over the calf’s muzzle. A prior study showed that this system delivered aerosol to the upper respiratory tract as well as the bronchioles and alveoli (Bassel et al., 2019). Personnel wore powered air-purifying respirators (PAPRs) and N95 respirators were placed over the one-way exhalation valves on the calf masks to reduce room air contamination. Two control calves (calves C1 and C2) were randomly assigned to receive only PBS, to ensure that the aerosolization procedure did not affect the measured parameters.

### Clinical and hematologic responses

Calves were assessed prior to and at 1, 2, 4, 6, 12, 18 and 24 h following administration of aerosolized bacterial lysate or PBS without knowledge of treatment group. At these times, rectal temperature, respiratory rate and effort, heart rate, and mentation were assessed. Additional clinical signs including lack of appetite, coughing or nasal discharge were noted when present. Clinical scores were assigned as described in Additional file 11. Blood was collected into sodium citrate anticoagulant for measuring plasma fibrinogen and without anticoagulant for evaluation of serum haptoglobin (AHL, University of Guelph) prior to and 24 h following aerosolization of bacterial lysate or PBS.

### Bronchoalveolar lavage fluid analysis

Bronchoalveolar lavage fluid was collected from all calves prior to and 24 h following administration of the bacterial lysate. Calves were sedated with xylazine and BALF was collected from the right caudodorsal lung for the baseline sample collection and the left caudodorsal lung for the post-aerosol sample collection, by infusing 120 mL sterile saline (120 mL, 0.9%) through an endoscope and retrieving the lavage fluid by manual suction through a 60 mL syringe. The sample was placed on ice until further processing (within 2 h). Samples were filtered through gauze prior to determination of total nucleated cell counts using electrical impedance (Z2 Coulter counter, Beckman Coulter, Mississauga, ON) and cytocentrifuge preparations were prepared using 200 μL of fluid. Differential cell counts were performed on 200 cells on the Wright-stained slide preparations. Olympus CellSens software was used to trace the outline of individual cells and measure the area of 15 macrophages in a single central 400x field.

### Mass spectrometry analysis

For mass spectrometry, the remaining BALF was centrifuged (500×*g*, 5 min, 4 °C), then desalted and concentrated by centrifugal filtration (Amicon® Ultra-15, Millipore, Bedford, MA, USA). Protein concentrations were measured using a Bradford assay [35]. Tandem mass tag (TMT) mass spectrometry (SPARC BioCentre, The Hospital for Sick Children, Toronto, Canada) was conducted on four pre- and post-aerosolization samples (calves 1–4). The normalized protein concentrations were reported. Samples were reduced, alkylated, digested, and TMT labelled according to manufacturer’s directions (Thermo Fisher TMT 10 Plex, Product 90,110). Labelled peptides from all samples were combined and lyophilized. Peptides were cleaned up using a C18 ZipTip (Millipore) and then lyophilized.

Samples were analyzed on a Thermo Scientific Orbitrap Fusion-Lumos Tribid Mass Spectrometer (ThermoFisher, San Jose, CA) outfitted with a nanospray source and EASY-nLC 1200 nano-LC system (ThermoFisher, San Jose, CA) and equipped with ETD mode as described in Additional file 12. Data analysis was performed using Proteome Discoverer 2.2 against a Uniprot Bovine Database (6002 sequences). TMT modifications of lysine and the peptide N-termini as well as carbamidomethyl of cysteine were considered fixed modifications while oxidation of methionine and protein N-terminal acetylation were considered variable. Parent mass tolerance was set to 10 ppm, fragment mass tolerance was set to 0.6 Da. Reporter ion quantification for the 8 TMT channels was done on the MS3, and lot-specific correction factors were used. Proteins that were significantly increased or decreased following lysate treatment were analysed in PANTHER v14.1 (www.pantherdb.org/) to analyze function. The pathways overrepresented among the upregulated and down regulated proteins were analyzed (Reactome version 68, www.Reactome.org; [37]) using a Fisher’s exact test to produce a probability score that was corrected for false discovery rate using the Benjamani-Hochberg method.

### Cytokine analysis

The following cytokines were measured in BALF: IFN-α, IL-8, IL-2, IL-6, tumour necrosis factor alpha (TNF-α), IFN-γ, IL-10, and IL-17 using a multiplexed electrochemiluminescent ligand-binding assay with the U-plex assay platform (MSD, Rockville, MD). The assay was developed using two separate Meso Scale Diagnostics (MSD) U-Plex panels, following the MSD U-Plex protocol guide. Cytokines were quantified using the U-plex assay platform (MSD, Rockville, MD) assembled according to manufacturer’s instructions using a chemiluminescent readout. Panel 1 included biotinylated capture antibodies: IL-1β (Biorad, Hercules, CA), IL-2 (R&D Systems Minneapolis, MN), IL-6 (R&D Systems), IFN-α (Kingfisher Biotech, Minneapolis, MN), and TNF-α (R&D Systems). Panel 2 included: IL-8 (Mabtech, Cincinnati, OH), IL-10 (Biorad), IL-17A (Mabtech), and IFN-γ (Mabtech). The IL-1β antibody was biotinylated using EZ-Link Sulfo-NHS-Biotin (Thermo Scientific) at a challenge ratio of 1:20 according to manufacturer instructions.

All calibrators used in the assay were purchased from Kingfisher Biotech. The panel 1 calibrators were diluted to a concentration of 40,000 pg/mL in 1X Dulbecco’s PBS without calcium or magnesium, followed by 4-fold serial dilutions into 1X DPBS. The panel 2 calibrators were diluted to a concentration of 10,000 pg/mL in 1X DPBS followed by 4-fold serial dilutions.

The detection antibodies were sulfo-tagged following the MSD quick guide conjugation protocol using a challenge ratio of 1:20. Panel 1 detection antibodies were used at the following concentrations: IL-1β at 1 μg/mL (Biorad), IL-2 at 0.5 μg/mL (R&D Systems), IL-6 at 2 μg/mL (R&D Systems), IFN-α at 1 μg/mL (Kingfisher Biotech) and TNF-α at 0.5 μg/mL (R&D systems). Panel 2 detection antibodies were used at the following concentrations: IL-8 at 0.5 μg/mL (Mabtech), IL-10 at 1 μg/mL (Biorad), IL-17A at 0.2 μg/mL (Kingfisher Biotech) and IFN-γ at 1 μg/mL (Mabtech).

Cytokines were quantified using the U-plex assay platform (MSD, Rockville, MD) assembled according to manufacturer’s instructions using a chemiluminescent readout (Additional file 12). For soluble protein levels, a BCA protein kit (Thermofisher, Rockford, IL) was used. The cytokine concentrations were normalized to the total protein levels for each sample.

### Post-mortem examination

All but one of the lysate-treated calves were euthanized at 24 h following administration of aerosols, by intravenous injection of pentobarbital. Gross postmortem examination was performed within 2 h of death including visual inspection and palpation of respiratory tissues. The lungs (trachea removed) and heart were weighed and the lung: heart weight ratio was calculated for each calf. Samples of nasal mucosa, trachea and cranioventral, caudodorsal and caudoventral regions of lung were placed in 10% formalin, and histologic sections were routinely prepared and stained with hematoxylin and eosin.

### Statistical analysis

Data were compared between samples obtained before and after aerosolization of bacterial lysate within the same animal. Descriptive statistics, t-tests and ANOVA were performed (Graphpad Prism v8.0, San Diego, CA, USA) and 2-sided tests were considered significant when *P <* 0.05. Outcome variables were evaluated for normality using a D’Agostino-Pearson omnibus K2 test, Shapiro-Wilk test and Kolmogorov-Smirnov test and transformed as indicated. Grubb’s test, with alpha set at 1%, was used to detect outliers, which were subsequently removed from further analysis.

ANOVA compared the effects of bacterial lysate on normalized ratios of TAP: GAPDH and LAP: GAPDH expression in cultured tracheal epithelial cells. Repeated measures of clinical parameters including rectal temperatures, respiratory rate, and heart rate were evaluated using repeated measures one-way ANOVA with a Geisser-Greenhouse correction to adjust for unequal variability of differences. Multiple comparisons of post-aerosolization values against baseline (time 0) values were evaluated using a Dunnett’s test. Residuals were evaluated to determine whether ANOVA assumptions were met. For clinical scores, a repeated measure non-parametric Friedman test was conducted, with post hoc Dunn’s tests to compare post-aerosolization clinical scores with baseline.

Paired t-tests were used to compare baseline and post-stimulation concentrations of serum haptoglobin, plasma fibrinogen, hematology values and BALF parameters (cell counts and cytokines). For the cytokine assays, analytes that were below the limit of detection were assigned a value that was equal to the lower limit of detection divided by the square root of 2 [36]. Outcome variables that were not normally distributed and were not normalized following transformation were assessed non-parametrically using a Wilcoxon matched-pairs sign rank test.

Two-sided tests with α < 0.05 were considered significant. Data from the study is provided as Additional file 13.

## Supplementary information

**Additional file 1.** Clinical parameters over time following aerosolization in 6 Holstein calves following aerosolization of bacterial lysate.

**Additional file 2.** Bronchoalveolar lavage fluid cells pre- and post- administration of differing doses of bacterial lysate or saline in Holstein bull calves.

**Additional file 3.** Bronchoalveolar lavage fluid cytology before and 24 h after aerosolization of bacterial lysate.

**Additional file 4.** Surface area of macrophages before and after aerosolization of bacterial lysate.

**Additional file 5.** Bronchoalveolar lavage fluid protein concentrations in calves prior to and 24 and 96 h post-aerosolization of bacterial lysate or saline.

**Additional file 6. **Mass spectrometry data for proteins in bronchoalveolar lavage fluid in 4 calves (A4, A3, A5, A6) before and 24 h after aerosolization with heat-killed lysate of *Escherichia coli* and *Staphylococcus aureus*.

**Additional file 7.** PANTHER classification of 19 upregulated proteins and 26 downregulated proteins in bronchoalveolar lavage fluid following aerosolization of bacterial lysate in 4 calves.

**Additional file 8.** Reactome pathway analysis for differently expressed proteins in bronchoalveolar lavage fluid in 4 calves.

**Additional file 9. **Gross lung lesions at 24 h after aerosol administration of *Staphylococcus aureus* and *Escherichia coli* lysate.

**Additional file 10. **Histologic lesions in respiratory tissues from 4 calves treated with aerosolized *Staphylococcus aureus* and *Escherichia coli* lysate.

**Additional file 11.** Clinical scoring system.

**Additional File 12.** Additional methods for cytokine analysis and mass spectrometry.

**Additional file 13.** Data.

## Data Availability

All data generated or analysed during this study are included in this published article and its supplementary information files.

## Abbreviations

AHL: Animal Health Laboratory
ANOVA: One-way analysis of variance
BALF: Bronchoalveolar lavage fluid
BCA: Bicinchoninic acid
CFU: Colony forming units
GAPDH: Glyceraldehyde-3-phosphate dehydrogenase
IFN: Interferon
IL: Interleukin
LAP: Lingual antimicrobial peptide
LPS: Lipopolysaccharide
NTHi: Nontypeable *Haemophilus influenzae*
PAMPs: Pathogen-associated molecular patterns
PBS: Phosphate-buffered saline
TAP: Tracheal antimicrobial peptide
TLR: Toll-like receptor
TMT: Tandem mass tag
TNF: Tumor necrosis factor
