# Supplementary material for: Pulmonary and systemic responses to aerosolized lysate of Staphylococcus aureus and Escherichia coli in calves
Source: BMC Vet Res. 2020 May 29;16:168. doi: 10.1186/s12917-020-02383-7 (PMC7260748; doi:10.1186/s12917-020-02383-7)
Supplement: Supplementary file 1 — Additional file 1. Clinical parameters over time following aerosolization in 6 Holstein calves following aerosolization of bacterial lysate. [file 12917_2020_2383_MOESM1_ESM.docx]

Additional File 1. Clinical parameters over time in 6 Holstein calves following aerosolization of bacterial lysate.


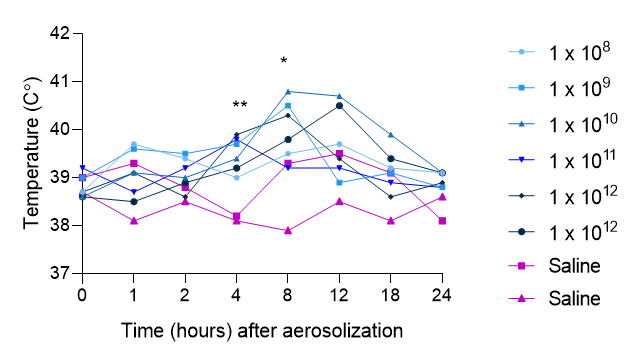


Data were compared to pre-challenge (time=0) using repeated measures one-way analysis of variance (ANOVA) with post hoc Dunnett’s multiple comparison test. **p* < 0.05, ***p* < 0.01. The mean, standard error of the mean and lower and upper confidence intervals (CI) are presented.

| Outcome variable | ANOVA results | P- value | Time (hours) | Mean | SEM | Lower 95% CI | Upper 95% CI |
| --- | --- | --- | --- | --- | --- | --- | --- |
| Rectal temperature | F (2.65, 13.27) = 4.95 | 0.019* | 0 | 38.8 | 0.24 | 38.5 | 39.1 |
|  |  |  | 1 | 39.1 | 0.48 | 38.6 | 39.6 |
|  |  |  | 2 | 39.1 | 0.33 | 38.8 | 39.5 |
|  |  |  | 4 | 39.5** | 0.36 | 39.1 | 39.9 |
|  |  |  | 8 | 40.0* | 0.62 | 39.4 | 40.7 |
|  |  |  | 12 | 39.7 | 0.72 | 39.0 | 40.5 |
|  |  |  | 18 | 39.2 | 0.44 | 38.7 | 39.7 |
|  |  |  | 24 | 39.0 | 0.15 | 38.8 | 39.1 |
| Heart rate | F (3.52, 17.60) = 3.05) | <0.050* | 0 | 77.0 | 7.6 | 57.4 | 96.6 |
|  |  |  | 1 | 79.3 | 7.0 | 61.4 | 97.2 |
|  |  |  | 2 | 86.0 | 8.8 | 63.4 | 108.6 |
|  |  |  | 4 | 95.3 | 8.1 | 74.5 | 116.1 |
|  |  |  | 8 | 98.7 | 6.0 | 83.3 | 114.1 |
|  |  |  | 12 | 89.3 | 4.9 | 76.7 | 102.0 |
|  |  |  | 18 | 77.7 | 5.7 | 63.1 | 92.2 |
|  |  |  | 24 | 75.7 | 2.0 | 70.6 | 80.7 |
| Respiratory rate | F (2.25, 11.24) = 1.89 | 0.195 | 0 | 54.3 | 4.7 | 42.1 | 66.5 |
|  |  |  | 1 | 62.0 | 10.9 | 34.1 | 89.9 |
|  |  |  | 2 | 78.0 | 5.4 | 64.0 | 92.0 |
|  |  |  | 4 | 68.3 | 4.7 | 56.3 | 80.4 |
|  |  |  | 8 | 67.7 | 6.1 | 51.9 | 83.4 |
|  |  |  | 12 | 79.3 | 5.6 | 64.9 | 93.7 |
|  |  |  | 18 | 66.3 | 2.6 | 59.8 | 72.9 |
|  |  |  | 24 | 61.0 | 4.5 | 49.5 | 72.6 |

­
