# Supplementary material for: Pulmonary and systemic responses to aerosolized lysate of Staphylococcus aureus and Escherichia coli in calves
Source: BMC Vet Res. 2020 May 29;16:168. doi: 10.1186/s12917-020-02383-7 (PMC7260748; doi:10.1186/s12917-020-02383-7)
Supplement: Supplementary file 2 — Additional file 2. Bronchoalveolar lavage fluid cells pre- and post- administration of differing doses of bacterial lysate or saline in Holstein bull calves. [file 12917_2020_2383_MOESM2_ESM.docx]

Additional File 2. **
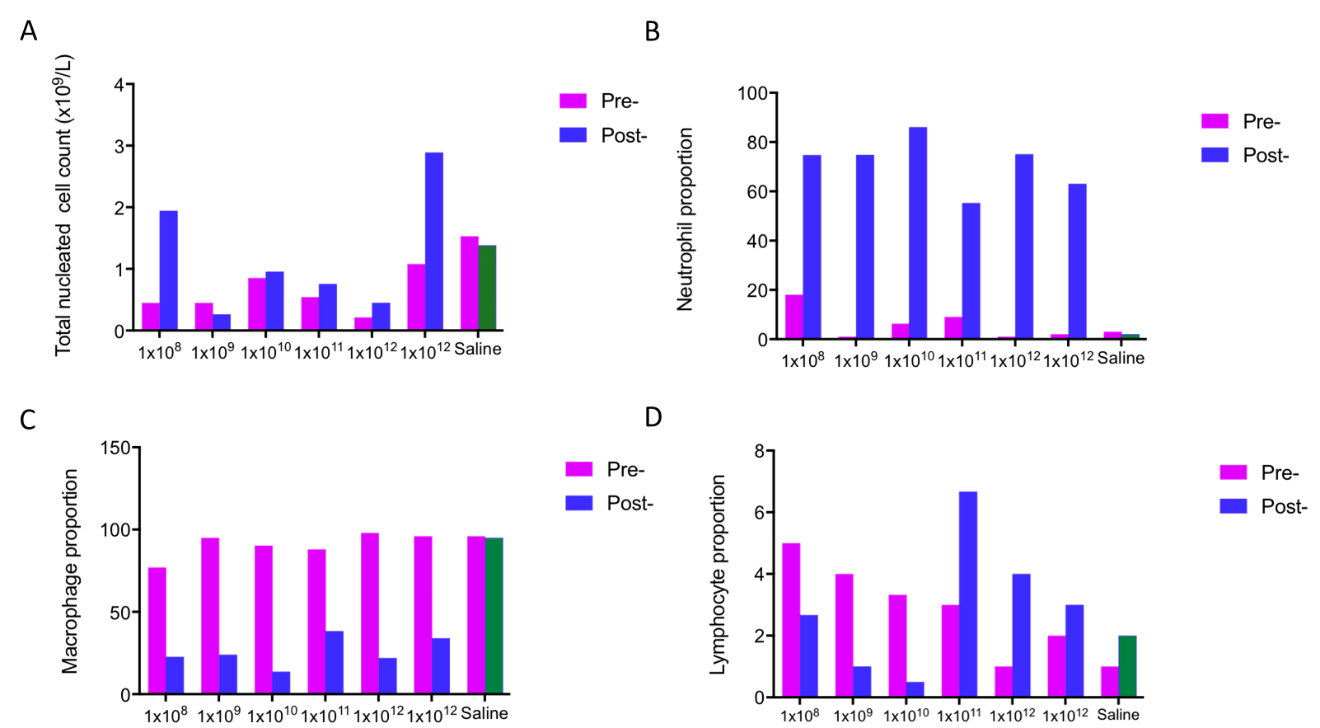
**Bronchoalveolar lavage fluid cells pre- and post- administration of differing doses of bacterial lysate or saline in Holstein bull calves. A) Total nucleated cell count. B) Percentage of neutrophils. C) Percentage of macrophages. D) Percentage of lymphocytes.
