# Supplementary material for: Pulmonary and systemic responses to aerosolized lysate of Staphylococcus aureus and Escherichia coli in calves
Source: BMC Vet Res. 2020 May 29;16:168. doi: 10.1186/s12917-020-02383-7 (PMC7260748; doi:10.1186/s12917-020-02383-7)
Supplement: Supplementary file 3 — Additional file 3. Bronchoalveolar lavage fluid cytology before and 24 h after aerosolization of bacterial lysate. [file 12917_2020_2383_MOESM3_ESM.docx]

Additional File 3. Bronchoalveolar lavage fluid cytology before (left: A, C, E, G) and 24 hours after (right: B, D, F, H) aerosolization of bacterial lysate. The lysate doses were 10^8^ (B), 10^9^ (D), 10^10^ (F), and 10^11^ (H) colony forming unit equivalents. Wright stain.

**
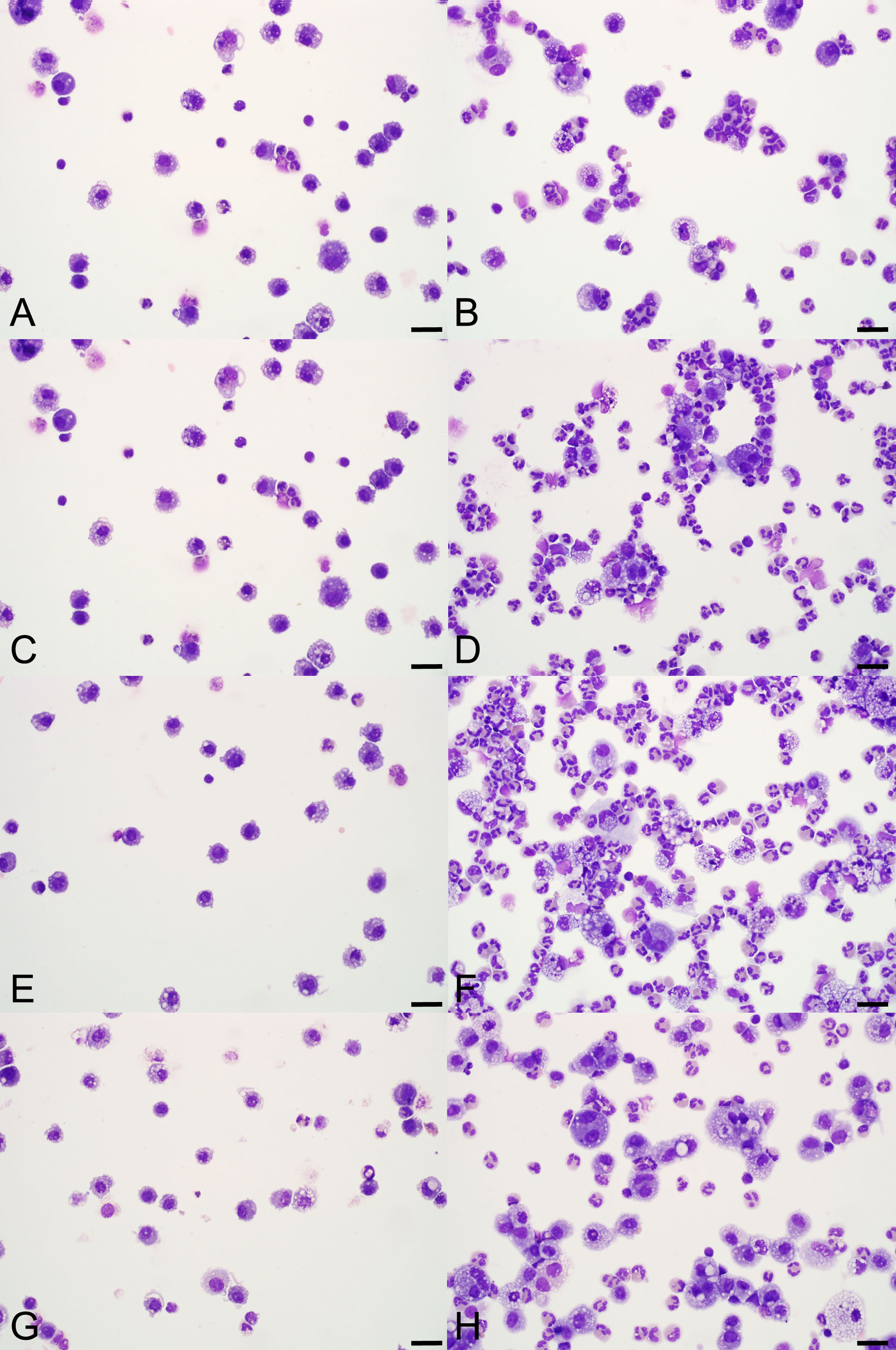
**
