# Supplementary material for: Pulmonary and systemic responses to aerosolized lysate of Staphylococcus aureus and Escherichia coli in calves
Source: BMC Vet Res. 2020 May 29;16:168. doi: 10.1186/s12917-020-02383-7 (PMC7260748; doi:10.1186/s12917-020-02383-7)

Additional File 4. Surface area of macrophages before and after aerosolization of bacterial lysate.

Cytocentrifuge preparations of bronchoalveolar lavage fluid taken before (A) and 24 hours post after (B) aerosol aerosol administration of 10^10^ colony forming unit (cfu)-equivalents of bacterial lysate. Wright stain. C) Macrophage surface area as determined by image analysis. The bars show mean and standard error of the mean of 15 cells prior to and 24-hours after aerosolization of bacterial lysate at dosages ranging from 10^8^ to 10^11^ cfu-equivalents. Cell size increased following lysate treatment (<0.0001, 2-way ANOVA).


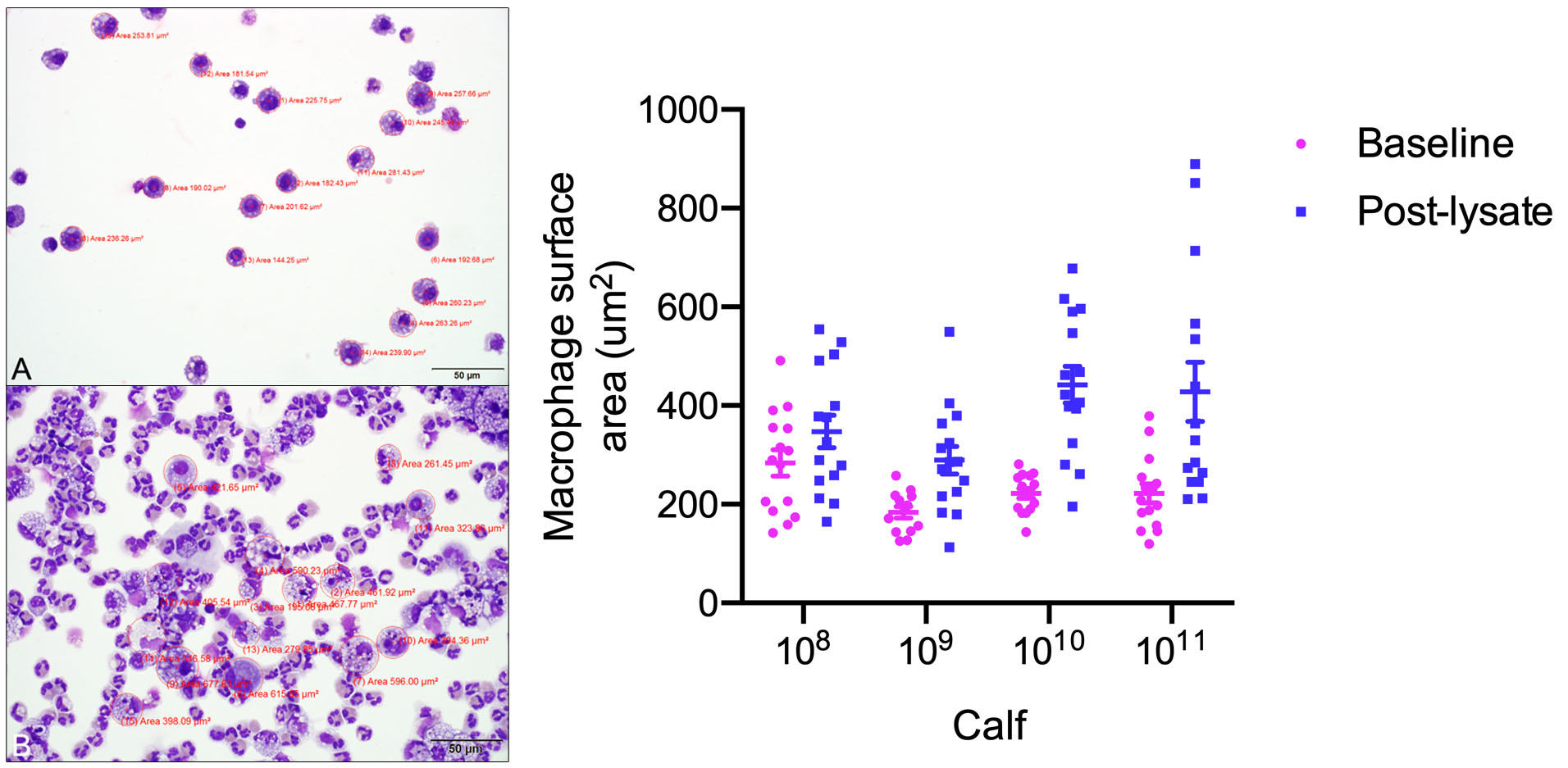

Supplement: Supplementary file 4 — Additional file 4. Surface area of macrophages before and after aerosolization of bacterial lysate. [file 12917_2020_2383_MOESM4_ESM.docx]
