# Supplementary material for: Pulmonary and systemic responses to aerosolized lysate of Staphylococcus aureus and Escherichia coli in calves
Source: BMC Vet Res. 2020 May 29;16:168. doi: 10.1186/s12917-020-02383-7 (PMC7260748; doi:10.1186/s12917-020-02383-7)
Supplement: Supplementary file 5 — Additional file 5. Bronchoalveolar lavage fluid protein concentrations in calves prior to and 24 and 96 h post-aerosolization of bacterial lysate or saline. [file 12917_2020_2383_MOESM5_ESM.docx]

Additional File 5. Bronchoalveolar lavage fluid protein concentrations (g/L) in calves prior to and 24 and 96 hours post aerosolization of bacterial lysate or saline.

| Treatment | Protein concentration (g/L) | |
| --- | --- | --- |
|  | **Baseline** | **After aerosolization** |
| Lysate (1x10^12^) | 77.65 | 195.37 |
| Lysate (1x10^12^) | 42.23 | 119.5 |
| Lysate (1x10^8^) | 69.89 | 144.58 |
| Lysate (1x10^9^) | 69.81 | 870.81 |
| Lysate (1x10^10^) | 40.00 | 91.46 |
| Lysate (1x10^11^) | 45.11 | 60.56 |
| Control | 73.08 | 65.66 |
| Control | 81.57 | 42.67 |

Lysate—aerosolized lysate of killed *S. aureus* and *E. coli*, doses are shown as cfu-equivalents; Control—aerosolized phosphate-buffered saline.
