# Supplementary material for: Pulmonary and systemic responses to aerosolized lysate of Staphylococcus aureus and Escherichia coli in calves
Source: BMC Vet Res. 2020 May 29;16:168. doi: 10.1186/s12917-020-02383-7 (PMC7260748; doi:10.1186/s12917-020-02383-7)
Supplement: Supplementary file 6 — Additional file 6. Mass spectrometry data for proteins in bronchoalveolar lavage fluid in 4 calves (A4, A3, A5, A6) before and 24 h after aerosolization with heat-killed lysate of Escherichia coli and Staphylococcus aureus. [file 12917_2020_2383_MOESM6_ESM.docx]

Additional File 6. Mass spectrometry data for proteins in bronchoalveolar lavage fluid in 4 calves (A4, A3, A5, A6) before and 24 hours after aerosolization with heat-killed lysate of *Escherichia coli* and *Staphylococcus aureus*. The mean ratio between pre and post-treatment values and the uncorrected *p-*value from a paired t test are also shown.

| ID | Description | Baseline | | | | Post-aerosolization | | | |  | |
| --- | --- | --- | --- | --- | --- | --- | --- | --- | --- | --- | --- |
|  |  | 2 | 1 | 3 | 4 | 2 | 1 | 3 | 4 | Mean ratio:  Pre: post | *P-*value |
| A0A140T897 | Serum albumin | 38 | 114 | 56 | 66 | 184 | 108 | 113 | 122 | 1.74 | 0.14 |
| Q29443 | Serotransferrin | 41 | 101 | 51 | 81 | 118 | 128 | 131 | 148 | 1.94 | 0.01 |
| G3X6N3 | Serotransferrin | 36 | 103 | 41 | 84 | 162 | 103 | 135 | 137 | 1.87 | 0.09 |
| G3X7A5 | Complement C3 | 69 | 119 | 70 | 95 | 79 | 113 | 125 | 132 | 1.25 | 0.17 |
| P48644 | Retinal dehydrogenase 1 | 159 | 83 | 129 | 53 | 64 | 108 | 100 | 105 | 0.80 | 0.75 |
| P24627 | Lactotransferrin | 17 | 50 | 27 | 299 | 21 | 157 | 115 | 114 | 0.99 | 0.97 |
| P81265 | Polymeric immunoglobulin receptor | 48 | 116 | 68 | 182 | 26 | 83 | 73 | 205 | 0.79 | 0.64 |
| Q7SIH1 | Alpha-2-macroglobulin | 58 | 45 | 53 | 40 | 232 | 149 | 150 | 71 | 2.70 | 0.04 |
| Q2KJ32 | Methanethiol oxidase | 290 | 75 | 166 | 87 | 39 | 53 | 44 | 45 | 0.28 | 0.13 |
| G3X6I0 | Uncharacterized protein | 75 | 212 | 67 | 114 | 14 | 98 | 33 | 186 | 0.40 | 0.44 |
| Q76LV2 | Heat shock protein HSP 90-alpha | 93 | 92 | 136 | 94 | 71 | 112 | 87 | 116 | 0.89 | 0.70 |
| F1N076 | Ceruloplasmin OS=Bos taurus CP PE=1 SV=2 | 46 | 100 | 25 | 34 | 207 | 132 | 156 | 101 | 2.72 | 0.04 |
| F1N650 | Annexin OS=Bos taurus ANXA1 PE=3 SV=1 | 138 | 60 | 118 | 105 | 56 | 160 | 111 | 52 | 0.68 | 0.82 |
| P19120 | Heat shock cognate 71 kDa protein OS=Bos taurus HSPA8 PE=1 SV=2 | 119 | 88 | 114 | 117 | 48 | 83 | 109 | 124 | 0.73 | 0.38 |
| P81187 | Complement factor B OS=Bos taurus CFB PE=1 SV=2 | 37 | 82 | 58 | 72 | 123 | 130 | 136 | 163 | 2.22 | 0.00 |
| E1B8H8 | SEC14 like lipid binding 3 OS=Bos taurus SEC14L3 PE=4 SV=1 | 155 | 86 | 211 | 44 | 26 | 83 | 62 | 133 | 0.37 | 0.45 |
| O77834 | Peroxiredoxin-6 OS=Bos taurus PRDX6 PE=1 SV=3 | 244 | 80 | 196 | 50 | 21 | 82 | 68 | 59 | 0.24 | 0.22 |
| F1N5M2 | Vitamin D-binding protein OS=Bos taurus GC PE=3 SV=2 | 31 | 86 | 42 | 59 | 248 | 98 | 119 | 118 | 2.16 | 0.13 |
| P60712 | Actin, cytoplasmic 1 OS=Bos taurus ACTB PE=1 SV=1 | 117 | 82 | 154 | 129 | 65 | 93 | 95 | 66 | 0.64 | 0.10 |
| A2I7M9 | Serpin A3-2 OS=Bos taurus SERPINA3-2 PE=3 SV=1 | 54 | 157 | 103 | 118 | 48 | 118 | 114 | 87 | 0.85 | 0.25 |
| Q3MHM5 | Tubulin beta-4B chain OS=Bos taurus TUBB4B PE=2 SV=1 | 57 | 168 | 55 | 161 | 218 | 63 | 36 | 41 | 0.48 | 0.77 |
| Q3SZV7 | Hemopexin OS=Bos taurus HPX PE=2 SV=1 | 38 | 111 | 64 | 114 | 75 | 105 | 148 | 145 | 1.44 | 0.14 |
| P15497 | Apolipoprotein A-I OS=Bos taurus APOA1 PE=1 SV=3 | 33 | 32 | 7 | 27 | 398 | 98 | 153 | 52 | 4.15 | 0.14 |
| F1MB08 | Alpha-enolase OS=Bos taurus ENO1 PE=3 SV=1 | 108 | 73 | 118 | 135 | 63 | 95 | 114 | 94 | 0.80 | 0.35 |
| G3X6K8 | Haptoglobin OS=Bos taurus HP PE=3 SV=1 | 10 | 64 | 13 | 217 | 71 | 216 | 142 | 67 | 1.06 | 0.53 |
| P00735 | Prothrombin OS=Bos taurus F2 PE=1 SV=2 | 38 | 86 | 83 | 174 | 96 | 105 | 126 | 93 | 1.07 | 0.77 |
| F1MYX5 | Lymphocyte cytosolic protein 1 OS=Bos taurus LCP1 PE=1 SV=1 | 65 | 104 | 62 | 184 | 47 | 117 | 121 | 101 | 0.87 | 0.83 |
| P81947 | Tubulin alpha-1B chain OS=Bos taurus OX=9913 PE=1 SV=2 | 164 | 95 | 170 | 84 | 55 | 74 | 84 | 75 | 0.54 | 0.10 |
| P02070 | Hemoglobin subunit beta OS=Bos taurus HBB PE=1 SV=1 | 87 | 23 | 9 | 72 | 60 | 148 | 362 | 38 | 1.14 | 0.33 |
| A0A140T8C8 | Kininogen-1 OS=Bos taurus KNG1 PE=4 SV=1 | 45 | 86 | 32 | 70 | 193 | 111 | 157 | 106 | 2.14 | 0.07 |
| Q76LV1 | Heat shock protein HSP 90-beta OS=Bos taurus HSP90AB1 PE=2 SV=3 | 96 | 102 | 90 | 102 | 109 | 112 | 108 | 81 | 1.03 | 0.60 |
| A0A140T8A5 | Isocitrate dehydrogenase [NADP] OS=Bos taurus IDH1 PE=1 SV=1 | 201 | 71 | 245 | 45 | 53 | 82 | 48 | 56 | 0.38 | 0.23 |
| P81287 | Annexin A5 OS=Bos taurus ANXA5 PE=1 SV=3 | 165 | 35 | 437 | 55 | 20 | 12 | 39 | 38 | 0.17 | 0.20 |
| F6QVC9 | Annexin OS=Bos taurus ANXA5 PE=1 SV=1 | 317 | 68 | 230 | 41 | 21 | 51 | 38 | 36 | 0.17 | 0.17 |
| E1BI82 | Inhibitor of carbonic anhydrase precursor OS=Bos taurus LOC525947 PE=1 SV=2 | 43 | 192 | 91 | 98 | 33 | 119 | 72 | 152 | 0.83 | 0.68 |
| P28801 | Glutathione S-transferase P OS=Bos taurus GSTP1 PE=1 SV=2 | 239 | 89 | 237 | 52 | 15 | 55 | 50 | 62 | 0.17 | 0.15 |
| A5D984 | Pyruvate kinase OS=Bos taurus PKM2 PE=1 SV=1 | 118 | 66 | 155 | 144 | 57 | 95 | 95 | 70 | 0.62 | 0.18 |
| Q27965 | Heat shock 70 kDa protein 1B OS=Bos taurus HSPA1B PE=2 SV=1 | 98 | 91 | 124 | 86 | 66 | 138 | 95 | 101 | 0.93 | 0.99 |
| Q29437 | Primary amine oxidase, liver isozyme OS=Bos taurus OX=9913 PE=1 SV=1 | 36 | 75 | 28 | 50 | 179 | 145 | 163 | 124 | 3.09 | 0.01 |
| E1BH06 | Uncharacterized protein OS=Bos taurus C4A PE=1 SV=2 | 53 | 78 | 36 | 64 | 131 | 103 | 193 | 142 | 2.23 | 0.05 |
| F1MSZ6 | Antithrombin-III OS=Bos taurus SERPINC1 PE=3 SV=1 | 40 | 101 | 68 | 74 | 206 | 95 | 111 | 105 | 1.55 | 0.21 |
| F1N1I6 | Gelsolin OS=Bos taurus GSN PE=1 SV=1 | 173 | 83 | 112 | 63 | 118 | 82 | 84 | 85 | 0.88 | 0.43 |
| Q5E9F5 | Transgelin-2 OS=Bos taurus TAGLN2 PE=2 SV=3 | 176 | 81 | 143 | 73 | 41 | 102 | 87 | 98 | 0.53 | 0.41 |
| P48034 | Aldehyde oxidase 1 OS=Bos taurus AOX1 PE=1 SV=2 | 233 | 68 | 198 | 62 | 59 | 69 | 59 | 52 | 0.42 | 0.17 |
| G3N1U4 | Serpin A3-3 OS=Bos taurus SERPINA3-3 PE=3 SV=1 | 27 | 70 | 56 | 79 | 161 | 95 | 178 | 137 | 2.23 | 0.05 |
| Q3SZH7 | Leukotriene A-4 hydrolase OS=Bos taurus LTA4H PE=2 SV=3 | 119 | 82 | 115 | 62 | 82 | 142 | 132 | 66 | 1.05 | 0.61 |
| Q58CQ9 | Pantetheinase OS=Bos taurus VNN1 PE=1 SV=1 | 50 | 103 | 68 | 178 | 31 | 82 | 74 | 213 | 0.86 | 0.99 |
| F1MMD7 | Inter-alpha-trypsin inhibitor heavy chain H4 OS=Bos taurus ITIH4 PE=4 SV=2 | 30 | 48 | 24 | 46 | 202 | 137 | 242 | 71 | 3.21 | 0.06 |
| Q3ZEJ6 | Serpin A3-3 OS=Bos taurus SERPINA3-3 PE=1 SV=2 | 34 | 108 | 97 | 119 | 130 | 106 | 121 | 86 | 1.16 | 0.49 |
| G8JKW7 | Uncharacterized protein OS=Bos taurus SERPINA3 PE=3 SV=1 | 107 | 103 | 156 | 92 | 87 | 65 | 81 | 110 | 0.72 | 0.24 |
| A2VE41 | EGF-containing fibulin-like extracellular matrix protein 1 OS=Bos taurus EFEMP1 PE=1 SV=1 | 72 | 198 | 87 | 115 | 26 | 80 | 42 | 181 | 0.50 | 0.42 |
| Q2HJB8 | Tubulin alpha-8 chain OS=Bos taurus TUBA8 PE=2 SV=1 | 147 | 101 | 215 | 70 | . | 48 | 81 | 139 | 0.76 | 0.57 |
| Q3SZR3 | Alpha-1-acid glycoprotein OS=Bos taurus ORM1 PE=2 SV=1 | 65 | 112 | 60 | 137 | 112 | 107 | 134 | 73 | 1.01 | 0.70 |
| P13696 | Phosphatidylethanolamine-binding protein 1 OS=Bos taurus PEBP1 PE=1 SV=2 | 312 | 66 | 235 | 37 | 23 | 34 | 45 | 48 | 0.19 | 0.17 |
| F1MCF8 | Uncharacterized protein OS=Bos taurus LOC100297192 PE=1 SV=2 | 33 | 129 | 54 | 118 | 84 | 157 | 90 | 136 | 1.50 | 0.02 |
| F1MD73 | Uncharacterized protein OS=Bos taurus OX=9913 PE=1 SV=2 | 32 | 168 | 69 | 302 | 4 | 54 | 34 | 138 | 0.26 | 0.08 |
| P02081 | Hemoglobin fetal subunit beta OS=Bos taurus OX=9913 PE=1 SV=1 | 23 | 11 | . | 56 | 7 | 140 | 531 | 32 | 0.79 | 0.61 |
| P62261 | 14-3-3 protein epsilon OS=Bos taurus YWHAE PE=2 SV=1 | 184 | 92 | 139 | 90 | 14 | 69 | 77 | 135 | 0.23 | 0.33 |
| Q95L54 | Annexin A8 OS=Bos taurus ANXA8 PE=2 SV=1 | 112 | 99 | 116 | 79 | 29 | 114 | 118 | 133 | 0.63 | 0.93 |
| F1MN84 | Aminopeptidase OS=Bos taurus ANPEP PE=3 SV=2 | 81 | 149 | 74 | 186 | 25 | 62 | 54 | 170 | 0.50 | 0.08 |
| F1MSB7 | Plastin-3 OS=Bos taurus PLS3 PE=1 SV=1 | 264 | 79 | 233 | 36 | 46 | 64 | 42 | 37 | 0.30 | 0.16 |
| Q2KJF1 | Alpha-1B-glycoprotein OS=Bos taurus A1BG PE=1 SV=1 | 55 | 95 | 33 | 88 | 116 | 104 | 176 | 134 | 1.79 | 0.11 |
| F1MJK3 | Uncharacterized protein OS=Bos taurus OX=9913 PE=4 SV=2 | 32 | 57 | 12 | 48 | 215 | 142 | 201 | 92 | 3.52 | 0.04 |
| P52898 | Dihydrodiol dehydrogenase 3 OS=Bos taurus OX=9913 PE=2 SV=1 | 191 | 86 | 117 | 63 | 53 | 107 | 113 | 71 | 0.64 | 0.50 |
| F1MWQ2 | Annexin OS=Bos taurus ANXA3 PE=3 SV=1 | 323 | 60 | 262 | 48 | 15 | 38 | 30 | 25 | 0.12 | 0.14 |
| A0A0N4STN1 | Uncharacterized protein OS=Bos taurus OX=9913 PE=3 SV=1 | 8 | 61 | 26 | 477 | 10 | 106 | 69 | 43 | 0.31 | 0.51 |
| Q2HJ49 | Moesin OS=Bos taurus MSN PE=2 SV=3 | 113 | 72 | 125 | 159 | 42 | 106 | 100 | 83 | 0.61 | 0.27 |
| F1MAV0 | Fibrinogen beta chain OS=Bos taurus FGB PE=4 SV=2 | 106 | 57 | 59 | 62 | 133 | 172 | 142 | 69 | 1.63 | 0.10 |
| F1MQ37 | Myosin heavy chain 9 OS=Bos taurus MYH9 PE=1 SV=2 | 91 | 92 | 128 | 153 | 158 | 70 | 66 | 43 | 0.54 | 0.46 |
| P15781 | Pulmonary surfactant-associated protein B OS=Bos taurus SFTPB PE=1 SV=3 | 80 | 257 | 79 | 102 | 15 | 80 | 36 | 152 | 0.35 | 0.30 |
| P06868 | Plasminogen OS=Bos taurus PLG PE=1 SV=2 | 45 | 116 | 90 | 95 | 143 | 116 | 112 | 85 | 1.23 | 0.34 |
| P01045 | Kininogen-2 OS=Bos taurus KNG2 PE=1 SV=1 | 38 | 138 | 64 | 10 | 219 | 159 | 10 | 162 | 0.54 | 0.27 |
| A5PJE3 | Fibrinogen alpha chain OS=Bos taurus FGA PE=2 SV=1 | 87 | 48 | 82 | 53 | 246 | 138 | 96 | 52 | 1.56 | 0.17 |
| E1BBX5 | Uncharacterized protein OS=Bos taurus OX=9913 PE=4 SV=2 | 278 | 56 | 108 | 90 | 52 | 32 | 78 | 106 | 0.43 | 0.31 |
| G5E5T5 | Uncharacterized protein OS=Bos taurus OX=9913 PE=1 SV=1 | 41 | 227 | 32 | 307 | 27 | 57 | 41 | 68 | 0.37 | 0.19 |
| P17697 | Clusterin OS=Bos taurus CLU PE=1 SV=1 | 47 | 167 | 64 | 128 | 53 | 102 | 57 | 182 | 0.92 | 0.91 |
| P68138 | Actin, alpha skeletal muscle OS=Bos taurus ACTA1 PE=1 SV=1 | 127 | 81 | 182 | 113 | 54 | 84 | 92 | 67 | 0.57 | 0.09 |
| O97680 | Thioredoxin OS=Bos taurus TXN PE=3 SV=3 | 169 | 88 | 167 | 88 | 41 | 67 | 84 | 96 | 0.48 | 0.17 |
| Q9BGI1 | Peroxiredoxin-5, mitochondrial OS=Bos taurus PRDX5 PE=2 SV=2 | 231 | 61 | 233 | 63 | 28 | 67 | 62 | 57 | 0.28 | 0.18 |
| P34955 | Alpha-1-antiproteinase OS=Bos taurus SERPINA1 PE=1 SV=1 | 39 | 51 | 51 | 18 | 495 | 59 | 55 | 32 | 1.65 | 0.36 |
| P01966 | Hemoglobin subunit alpha OS=Bos taurus HBA PE=1 SV=2 | 112 | 26 | 19 | 67 | 23 | 137 | 380 | 36 | 0.57 | 0.44 |
| Q5E956 | Triosephosphate isomerase OS=Bos taurus TPI1 PE=2 SV=3 | 126 | 80 | 145 | 150 | 41 | 91 | 93 | 75 | 0.53 | 0.10 |
| F1MMK9 | Protein AMBP OS=Bos taurus AMBP PE=4 SV=2 | 53 | 90 | 27 | 65 | 164 | 112 | 178 | 112 | 2.15 | 0.07 |
| A0A0A0MP92 | Serpin A3-7 OS=Bos taurus SERPINA3-7 PE=1 SV=1 | 32 | 66 | 128 | 62 | 245 | 115 | 94 | 59 | 1.28 | 0.38 |
| Q0VCC0 | Calcyphosin OS=Bos taurus CAPS PE=2 SV=1 | 122 | 79 | 144 | 77 | 21 | 111 | 112 | 135 | 0.48 | 0.78 |
| E1B6Z6 | Lipocalin 2 OS=Bos taurus LCN2 PE=1 SV=2 | 14 | 64 | 23 | 273 | 18 | 218 | 99 | 92 | 0.95 | 0.86 |
| Q6RXL1 | Pulmonary surfactant-associated protein A OS=Bos taurus SFTPA1 PE=2 SV=2 | 98 | 90 | 209 | 100 | 32 | 46 | 39 | 186 | 0.36 | 0.42 |
| Q3T0P6 | Phosphoglycerate kinase 1 OS=Bos taurus PGK1 PE=2 SV=3 | 174 | 76 | 129 | 89 | 64 | 91 | 103 | 75 | 0.66 | 0.30 |
| P63103 | 14-3-3 protein zeta/delta OS=Bos taurus YWHAZ PE=1 SV=1 | 150 | 103 | 132 | 126 | 27 | 87 | 75 | 99 | 0.41 | 0.10 |
| A3KMV5 | Ubiquitin-like modifier-activating enzyme 1 OS=Bos taurus UBA1 PE=2 SV=1 | 152 | 86 | 134 | 117 | 46 | 81 | 78 | 106 | 0.56 | 0.15 |
| O18879 | Glutathione S-transferase A2 OS=Bos taurus GSTA2 PE=2 SV=4 | 381 | 52 | 210 | 27 | 37 | 17 | 32 | 43 | 0.20 | 0.19 |
| P04272 | Annexin A2 OS=Bos taurus ANXA2 PE=1 SV=2 | 147 | 170 | 165 | 84 | 39 | 71 | 77 | 47 | 0.40 | 0.01 |
| P35246 | Pulmonary surfactant-associated protein D OS=Bos taurus SFTPD PE=1 SV=2 | 30 | 375 | 63 | 70 | 14 | 135 | 40 | 74 | 0.54 | 0.32 |
| Q1JPB0 | Leukocyte elastase inhibitor OS=Bos taurus SERPINB1 PE=2 SV=2 | 55 | 94 | 52 | 127 | 72 | 157 | 172 | 71 | 1.16 | 0.40 |
| P21856 | Rab GDP dissociation inhibitor alpha OS=Bos taurus GDI1 PE=1 SV=1 | 145 | 81 | 152 | 101 | 45 | 103 | 84 | 90 | 0.58 | 0.25 |
| Q5E9F7 | Cofilin-1 OS=Bos taurus CFL1 PE=2 SV=3 | 166 | 63 | 137 | 103 | 36 | 84 | 112 | 99 | 0.53 | 0.38 |
| Q95121 | Pigment epithelium-derived factor OS=Bos taurus SERPINF1 PE=1 SV=1 | 44 | 89 | 120 | 52 | 222 | 69 | 67 | 137 | 1.09 | 0.43 |
| P49259 | Secretory phospholipase A2 receptor OS=Bos taurus PLA2R1 PE=1 SV=1 | 40 | 223 | 71 | 100 | 22 | 92 | 36 | 216 | 0.60 | 0.76 |
| Q8SPP7 | Peptidoglycan recognition protein 1 OS=Bos taurus PGLYRP1 PE=1 SV=1 | 8 | 80 | 28 | 332 | 19 | 218 | 75 | 41 | 0.44 | 0.82 |
| P79345 | NPC intracellular cholesterol transporter 2 OS=Bos taurus NPC2 PE=1 SV=1 | 53 | 226 | 68 | 142 | 22 | 71 | 55 | 163 | 0.52 | 0.33 |
| G3MXB5 | Uncharacterized protein OS=Bos taurus OX=9913 PE=1 SV=1 | 42 | 101 | 89 | 242 | 13 | 59 | 44 | 211 | 0.50 | 0.00 |
| P12763 | Alpha-2-HS-glycoprotein OS=Bos taurus AHSG PE=1 SV=2 | 43 | 127 | 42 | 52 | 201 | 104 | 114 | 118 | 1.78 | 0.16 |
| G1K122 | Retinol-binding protein OS=Bos taurus RBP4 PE=3 SV=1 | 60 | 141 | 60 | 74 | 68 | 109 | 99 | 189 | 1.26 | 0.38 |
| Q2KIF2 | Leucine-rich alpha-2-glycoprotein 1 OS=Bos taurus LRG1 PE=1 SV=1 | 25 | 78 | 34 | 55 | 257 | 91 | 144 | 117 | 2.40 | 0.11 |
| F1MY85 | Complement C5a anaphylatoxin OS=Bos taurus C5 PE=1 SV=2 | 65 | 152 | 59 | 76 | 114 | 85 | 103 | 146 | 1.16 | 0.49 |
| Q3ZCC8 | Tubulin polymerization-promoting protein family member 3 OS=Bos taurus TPPP3 PE=1 SV=1 | 150 | 63 | 230 | 54 | 29 | 93 | 58 | 122 | 0.39 | 0.46 |
| Q5E9B7 | Chloride intracellular channel protein 1 OS=Bos taurus CLIC1 PE=2 SV=3 | 114 | 97 | 114 | 125 | 60 | 100 | 95 | 95 | 0.74 | 0.13 |
| G3X757 | Transitional endoplasmic reticulum ATPase OS=Bos taurus VCP PE=3 SV=1 | 154 | 111 | 141 | 80 | 105 | 73 | 64 | 72 | 0.64 | 0.06 |
| F1MLW8 | Uncharacterized protein OS=Bos taurus OX=9913 PE=1 SV=2 | 55 | 68 | 34 | 221 | 102 | 98 | 163 | 59 | 0.77 | 0.87 |
| Q9N0V4 | Glutathione S-transferase Mu 1 OS=Bos taurus GSTM1 PE=1 SV=3 | 146 | 84 | 186 | 56 | 65 | 62 | 86 | 115 | 0.64 | 0.39 |
| F1MVJ8 | Olfactomedin 4 OS=Bos taurus OLFM4 PE=4 SV=1 | 5 | 36 | 22 | 328 | 32 | 164 | 145 | 68 | 0.75 | 0.96 |
| P10096 | Glyceraldehyde-3-phosphate dehydrogenase OS=Bos taurus GAPDH PE=1 SV=4 | 96 | 113 | 133 | 163 | 96 | 87 | 72 | 42 | 0.50 | 0.14 |
| F1MH40 | Uncharacterized protein OS=Bos taurus OX=9913 PE=1 SV=2 | 93 | 132 | 70 | 212 | . | 118 | 100 | 76 | 0.87 | 0.51 |
| F6Q751 | Uncharacterized protein OS=Bos taurus GSTM2 PE=1 SV=1 | 170 | 71 | 262 | 48 | 69 | 54 | 61 | 65 | 0.45 | 0.22 |
| G5E513 | Uncharacterized protein OS=Bos taurus OX=9913 PE=1 SV=1 | 69 | 264 | 57 | 156 | 8 | 85 | 64 | 96 | 0.28 | 0.15 |
| A6QPK0 | Mammaglobin-A precursor OS=Bos taurus SCGB2A2 PE=2 SV=1 | 89 | 190 | 59 | 160 | 19 | 69 | 41 | 174 | 0.40 | 0.20 |
| A6QLL8 | Fructose-bisphosphate aldolase OS=Bos taurus ALDOA PE=1 SV=1 | 121 | 73 | 104 | 104 | 124 | 91 | 76 | 108 | 0.97 | 0.92 |
| E1BHJ0 | Profilin OS=Bos taurus OX=9913 PE=3 SV=1 | 140 | 75 | 91 | 129 | 22 | 83 | 128 | 132 | 0.44 | 0.64 |
| E1BEL7 | Heat shock protein beta-1 OS=Bos taurus HSPB1 PE=3 SV=2 | 187 | 60 | 157 | 81 | 73 | 120 | 83 | 39 | 0.57 | 0.34 |
| Q3ZCI4 | 6-phosphogluconate dehydrogenase, decarboxylating OS=Bos taurus PGD PE=1 SV=2 | 112 | 95 | 142 | 106 | 41 | 92 | 127 | 84 | 0.65 | 0.16 |
| P33046 | Cathelicidin-4 OS=Bos taurus CATHL4 PE=1 SV=1 | 9 | 60 | 27 | 433 | 15 | 131 | 68 | 56 | 0.44 | 0.58 |
| Q3T0I2 | Pro-cathepsin H OS=Bos taurus CTSH PE=2 SV=1 | 48 | 252 | 82 | 116 | 42 | 107 | 44 | 110 | 0.62 | 0.24 |
| F1MVK1 | Uncharacterized protein OS=Bos taurus OX=9913 PE=4 SV=2 | 39 | 73 | . | 43 | 229 | 130 | 191 | 97 | 3.41 | 0.16 |
| F1MZ96 | Uncharacterized protein OS=Bos taurus OX=9913 PE=1 SV=2 | 43 | 69 | 40 | 93 | 47 | 87 | 135 | 287 | 1.71 | 0.17 |
| Q5E9B1 | L-lactate dehydrogenase B chain OS=Bos taurus LDHB PE=2 SV=4 | 218 | 84 | 134 | 105 | 51 | 84 | 80 | 44 | 0.43 | 0.14 |
| Q3ZBZ8 | Stress-induced-phosphoprotein 1 OS=Bos taurus STIP1 PE=2 SV=1 | 114 | 71 | 102 | 59 | 118 | 112 | 106 | 117 | 1.30 | 0.15 |
| G3X7D2 | Chitinase-3-like protein 1 OS=Bos taurus CHI3L1 PE=4 SV=1 | 34 | 122 | 55 | 104 | 47 | 136 | 80 | 222 | 1.44 | 0.19 |
| P50397 | Rab GDP dissociation inhibitor beta OS=Bos taurus GDI2 PE=2 SV=3 | 181 | 75 | 107 | 102 | 68 | 92 | 105 | 69 | 0.67 | 0.33 |
| H9GW43 | Ribonuclease inhibitor OS=Bos taurus RNH1 PE=1 SV=1 | 165 | 110 | 178 | 79 | 16 | 84 | 89 | 81 | 0.28 | 0.15 |
| Q3ZBS7 | Vitronectin OS=Bos taurus VTN PE=1 SV=1 | 63 | 81 | 59 | 44 | 114 | 134 | 175 | 129 | 2.19 | 0.02 |
| Q2KIT0 | Protein HP-20 homolog OS=Bos taurus OX=9913 PE=2 SV=1 | 29 | 92 | 24 | 55 | 262 | 133 | 126 | 80 | 2.40 | 0.12 |
| G3N0V0 | Uncharacterized protein OS=Bos taurus OX=9913 PE=1 SV=1 | 54 | 103 | 59 | 98 | 62 | 145 | 177 | 103 | 1.39 | 0.20 |
| F1MLW2 | BPI fold-containing family B member 1 OS=Bos taurus BPIFB1 PE=4 SV=1 | 48 | 101 | 59 | 264 | 42 | 68 | 86 | 131 | 0.75 | 0.37 |
| Q58D62 | Fetuin-B OS=Bos taurus FETUB PE=1 SV=1 | 46 | 88 | 30 | 62 | 305 | 101 | 109 | 59 | 1.71 | 0.24 |
| G3N2D7 | Uncharacterized protein OS=Bos taurus LOC100297192 PE=4 SV=1 | 38 | 96 | 35 | 152 | 87 | 127 | 118 | 146 | 1.58 | 0.13 |
| F1MUX6 | Glutathione S-transferase OS=Bos taurus GSTM3 PE=1 SV=2 | 78 | 117 | 137 | 118 | 21 | 68 | 95 | 167 | 0.52 | 0.39 |
| F1N3Q7 | Apolipoprotein A-IV OS=Bos taurus APOA4 PE=3 SV=1 | 30 | 43 | 15 | 19 | 449 | 89 | 125 | 30 | 3.07 | 0.21 |
| Q3SYU2 | Elongation factor 2 OS=Bos taurus EEF2 PE=2 SV=3 | 103 | 84 | 147 | 86 | 75 | 97 | 91 | 116 | 0.87 | 0.64 |
| Q3MHL4 | Adenosylhomocysteinase OS=Bos taurus AHCY PE=2 SV=3 | 111 | 90 | 154 | 111 | 44 | 93 | 122 | 75 | 0.65 | 0.11 |
| Q3T114 | 2-iminobutanoate/2-iminopropanoate deaminase OS=Bos taurus RIDA PE=2 SV=3 | 61 | 100 | 65 | 271 | 26 | 104 | 106 | 68 | 0.51 | 0.44 |
| F1MGU7 | Fibrinogen gamma-B chain OS=Bos taurus FGG PE=4 SV=1 | 100 | 51 | 30 | 51 | 134 | 162 | 196 | 76 | 2.12 | 0.09 |
| Q0VCU1 | Cytoplasmic aconitate hydratase OS=Bos taurus ACO1 PE=2 SV=1 | 156 | 80 | 149 | 61 | 31 | 112 | 125 | 86 | 0.52 | 0.57 |
| Q3SYR8 | Immunoglobulin J chain OS=Bos taurus JCHAIN PE=1 SV=1 | 32 | 113 | 122 | 296 | 12 | 79 | 30 | 115 | 0.37 | 0.11 |
| Q5E946 | Protein/nucleic acid deglycase DJ-1 OS=Bos taurus PARK7 PE=2 SV=1 | 206 | 75 | 169 | 67 | 46 | 83 | 84 | 70 | 0.48 | 0.24 |
| F6QS88 | Epoxide hydrolase 2 OS=Bos taurus EPHX2 PE=1 SV=1 | 103 | 120 | 151 | 64 | 44 | 104 | 90 | 125 | 0.71 | 0.56 |
| A0A140T854 | Glutathione S-transferase OS=Bos taurus GSTA3 PE=3 SV=1 | 57 | 173 | 47 | 99 | 39 | 183 | 142 | 61 | 0.92 | 0.70 |
| F1MM32 | Sulfhydryl oxidase OS=Bos taurus QSOX1 PE=4 SV=2 | 66 | 147 | 77 | 186 | 38 | 94 | 49 | 143 | 0.65 | 0.01 |
| E1BKZ9 | Sortilin OS=Bos taurus SORT1 PE=4 SV=1 | 49 | 220 | 72 | 106 | 9 | 78 | 35 | 231 | 0.36 | 0.70 |
| A6QPZ4 | SERPINB4 protein OS=Bos taurus SERPINB4 PE=2 SV=1 | 10 | 29 | 12 | 330 | 63 | 110 | 207 | 39 | 0.45 | 0.93 |
| Q3T145 | Malate dehydrogenase, cytoplasmic OS=Bos taurus MDH1 PE=2 SV=3 | 186 | 78 | 206 | 64 | 53 | 68 | 61 | 84 | 0.45 | 0.21 |
| A5D7D1 | Alpha-actinin-4 OS=Bos taurus ACTN4 PE=2 SV=1 | 85 | 77 | 122 | 153 | 122 | 87 | 96 | 58 | 0.73 | 0.56 |
| E1BBM1 | Glyoxalase domain containing 4 OS=Bos taurus GLOD4 PE=1 SV=2 | 137 | 79 | 128 | 63 | 82 | 114 | 100 | 97 | 0.93 | 0.89 |
| E1BD43 | Amine oxidase OS=Bos taurus AOC3 PE=3 SV=1 | 52 | 76 | 18 | 76 | 115 | 132 | 233 | 100 | 2.15 | 0.13 |
| P48616 | Vimentin OS=Bos taurus VIM PE=1 SV=3 | 29 | 93 | 58 | 199 | 67 | 112 | 148 | 94 | 1.06 | 0.81 |
| Q3SZ62 | Phosphoglycerate mutase 1 OS=Bos taurus PGAM1 PE=2 SV=3 | 124 | 107 | 150 | 110 | 36 | 99 | 85 | 89 | 0.53 | 0.09 |
| P31976 | Ezrin OS=Bos taurus EZR PE=1 SV=2 | 152 | 69 | 144 | 136 | 31 | 73 | 75 | 121 | 0.45 | 0.17 |
| Q0VCI3 | Carboxylic ester hydrolase OS=Bos taurus CES1 PE=1 SV=1 | 128 | 88 | 114 | 107 | 42 | 84 | 148 | 88 | 0.66 | 0.51 |
| Q0VCS8 | Glutathione S-transferase, theta 3 OS=Bos taurus GSTT3 PE=1 SV=1 | 358 | 65 | 221 | 35 | 22 | 37 | 41 | 21 | 0.16 | 0.16 |
| F6R3I5 | Cysteine-rich secretory protein 3 precursor OS=Bos taurus CRISP3 PE=3 SV=1 | 68 | 95 | 70 | 210 | 12 | 88 | 68 | 189 | 0.46 | 0.18 |
| P62935 | Peptidyl-prolyl cis-trans isomerase A OS=Bos taurus PPIA PE=1 SV=2 | 200 | 68 | 132 | 80 | 63 | 76 | 94 | 87 | 0.62 | 0.32 |
| A6QPP2 | SERPIND1 protein OS=Bos taurus SERPIND1 PE=2 SV=1 | 37 | 77 | 35 | 47 | 288 | 130 | 128 | 57 | 2.21 | 0.15 |
| F1MWU9 | Heat shock protein family A (Hsp70) member 6 OS=Bos taurus HSPA6 PE=1 SV=2 | 165 | 95 | 131 | 118 | 36 | 116 | 92 | 48 | 0.43 | 0.18 |
| F1MMR6 | Cathepsin D OS=Bos taurus CTSD PE=3 SV=1 | 51 | 150 | 97 | 136 | 24 | 83 | 83 | 177 | 0.69 | 0.51 |
| G5E604 | Uncharacterized protein OS=Bos taurus OX=9913 PE=1 SV=1 | 14 | 31 | 35 | 373 | 23 | 64 | 203 | 57 | 0.51 | 0.81 |
| F1N455 | Dipeptidyl peptidase 1 OS=Bos taurus CTSC PE=3 SV=1 | 45 | 187 | 32 | 129 | 18 | 160 | 134 | 96 | 0.76 | 0.92 |
| F1N2J8 | Chromosome 16 open reading frame 89 OS=Bos taurus C16orf89 PE=4 SV=2 | 82 | 112 | 55 | 121 | 29 | 105 | 98 | 199 | 0.78 | 0.64 |
| F1N1R0 | SEC14-like protein 4 OS=Bos taurus SEC14L4 PE=4 SV=2 | 200 | 67 | 154 | 32 | 100 | 80 | 90 | 78 | 0.80 | 0.49 |
| G3MWU1 | Thioredoxin reductase 1, cytoplasmic OS=Bos taurus TXNRD1 PE=3 SV=1 | 98 | 100 | 138 | 107 | 39 | 133 | 82 | 103 | 0.67 | 0.40 |
| Q28065 | C4b-binding protein alpha chain OS=Bos taurus C4BPA PE=2 SV=1 | 95 | 94 | 42 | 97 | 45 | 123 | 105 | 200 | 1.06 | 0.35 |
| Q08DP0 | Phosphoglucomutase-1 OS=Bos taurus PGM1 PE=2 SV=1 | 135 | 88 | 90 | 116 | 97 | 98 | 103 | 73 | 0.84 | 0.40 |
| Q0VCM4 | Glycogen phosphorylase, liver form OS=Bos taurus PYGL PE=2 SV=1 | 63 | 101 | 82 | 132 | 75 | 176 | 131 | 41 | 0.76 | 0.78 |
| A7E3W2 | Galectin-3-binding protein OS=Bos taurus LGALS3BP PE=1 SV=1 | 34 | 109 | 107 | 133 | 39 | 89 | 54 | 234 | 0.86 | 0.82 |
| F1MKS5 | Histidine-rich glycoprotein OS=Bos taurus HRG PE=4 SV=2 | 63 | 60 | 2 | 141 | 132 | 113 | 230 | 59 | 1.18 | 0.37 |
| P13214 | Annexin A4 OS=Bos taurus ANXA4 PE=1 SV=2 | 157 | 78 | 137 | 58 | 36 | 133 | 122 | 78 | 0.59 | 0.71 |
| F1N6C0 | Calmodulin OS=Bos taurus CALM2 PE=4 SV=2 | 153 | 102 | 161 | 117 | 8 | 80 | 81 | 97 | 0.17 | 0.11 |
| G5E5H2 | Uncharacterized protein OS=Bos taurus OX=9913 PE=4 SV=1 | 29 | 133 | 42 | 182 | . | 142 | 137 | 136 | 1.55 | 0.69 |
| F1MNQ4 | Superoxide dismutase [Cu-Zn] OS=Bos taurus OX=9913 PE=3 SV=2 | 175 | 82 | 246 | 85 | 9 | 52 | 64 | 87 | 0.16 | 0.14 |
| A5D7A0 | EF-hand domain-containing protein D2 OS=Bos taurus EFHD2 PE=2 SV=1 | 84 | 93 | 66 | 253 | 24 | 82 | 100 | 99 | 0.51 | 0.32 |
| F1MTP5 | WD repeat-containing protein 1 OS=Bos taurus WDR1 PE=4 SV=2 | 77 | 88 | 82 | 96 | 86 | 134 | 138 | 99 | 1.29 | 0.12 |
| Q5E947 | Peroxiredoxin-1 OS=Bos taurus PRDX1 PE=2 SV=1 | 161 | 103 | 148 | 108 | 37 | 73 | 74 | 96 | 0.45 | 0.10 |
| Q95132 | Intercellular adhesion molecule 1 OS=Bos taurus ICAM1 PE=2 SV=1 | 41 | 229 | 61 | 135 | 43 | 73 | 34 | 185 | 0.60 | 0.51 |
| Q9BGI3 | Peroxiredoxin-2 OS=Bos taurus PRDX2 PE=2 SV=1 | 176 | 58 | 165 | 67 | 31 | 93 | 142 | 68 | 0.47 | 0.46 |
| G5E5A9 | Fibronectin OS=Bos taurus FN1 PE=4 SV=1 | 48 | 107 | 58 | 70 | 287 | 90 | 69 | 71 | 1.26 | 0.40 |
| Q3SZX4 | Carbonic anhydrase 3 OS=Bos taurus CA3 PE=2 SV=3 | 103 | 34 | 547 | 22 | 60 | 10 | 6 | 19 | 0.04 | 0.32 |
| F1N789 | Vinculin OS=Bos taurus VCL PE=1 SV=1 | 160 | 79 | 115 | 83 | 99 | 80 | 93 | 92 | 0.84 | 0.34 |
| Q08E20 | S-formylglutathione hydrolase OS=Bos taurus ESD PE=2 SV=1 | 268 | 70 | 159 | 78 | 33 | 57 | 69 | 67 | 0.31 | 0.19 |
| H7BWW2 | Beta-hexosaminidase OS=Bos taurus HEXB PE=3 SV=1 | 30 | 106 | 72 | 237 | 20 | 123 | 81 | 131 | 0.79 | 0.49 |
| P09487 | Alkaline phosphatase, tissue-nonspecific isozyme OS=Bos taurus ALPL PE=1 SV=2 | 36 | 70 | 45 | 283 | 35 | 81 | 135 | 115 | 0.85 | 0.77 |
| Q28133 | Allergen Bos d 2 OS=Bos taurus OX=9913 PE=1 SV=1 | 107 | 167 | 254 | 75 | 15 | 50 | 45 | 87 | 0.24 | 0.11 |
| Q3SZI4 | 14-3-3 protein theta OS=Bos taurus YWHAQ PE=2 SV=1 | 191 | 78 | 161 | 72 | . | 91 | 92 | 115 | 1.23 | 0.90 |
| Q3ZBF7 | Prostaglandin E synthase 3 OS=Bos taurus PTGES3 PE=1 SV=1 | 162 | 83 | 131 | 86 | 33 | 107 | 88 | 110 | 0.50 | 0.45 |
| P55859 | Purine nucleoside phosphorylase OS=Bos taurus PNP PE=1 SV=3 | 214 | 72 | 89 | 148 | 29 | 79 | 127 | 44 | 0.32 | 0.32 |
| G8JKX0 | Spermadhesin-1 OS=Bos taurus SPADH1 PE=4 SV=1 | 83 | 270 | 58 | 178 | 17 | 56 | 22 | 115 | 0.29 | 0.10 |
| A7Z014 | TKT protein OS=Bos taurus TKT PE=1 SV=1 | 89 | 80 | 155 | 131 | 91 | 87 | 96 | 72 | 0.75 | 0.24 |
| P26452 | 40S ribosomal protein SA OS=Bos taurus RPSA PE=2 SV=4 | 134 | 106 | 74 | 112 | 24 | 78 | 118 | 155 | 0.48 | 0.75 |
| Q32PH8 | Elongation factor 1-alpha 2 OS=Bos taurus EEF1A2 PE=2 SV=1 | 164 | 89 | 124 | 109 | 101 | 87 | 75 | 51 | 0.62 | 0.05 |
| Q0VCN1 | NmrA-like family domain-containing protein 1 OS=Bos taurus NMRAL1 PE=2 SV=1 | 122 | 64 | 165 | 90 | 68 | 82 | 106 | 103 | 0.80 | 0.40 |
| G3X894 | Uncharacterized protein OS=Bos taurus OX=9913 PE=3 SV=1 | . | 42 | . | 391 | . | 122 | 225 | 21 | . | 0.64 |
| Q3SZH5 | Angiotensinogen (Serpin peptidase inhibitor, clade A, member 8) OS=Bos taurus AGT PE=1 SV=1 | 93 | 115 | 102 | 68 | 100 | 116 | 120 | 86 | 1.12 | 0.08 |
| A0A140T894 | 14-3-3 protein beta/alpha OS=Bos taurus YWHAB PE=3 SV=1 | 165 | 85 | 161 | 95 | 43 | 77 | 75 | 100 | 0.49 | 0.18 |
| Q6R8F2 | Cadherin-1 OS=Bos taurus CDH1 PE=2 SV=1 | 46 | 251 | 103 | 142 | 12 | 87 | 20 | 142 | 0.31 | 0.14 |
| E1BBP7 | Histone H4 OS=Bos taurus OX=9913 PE=3 SV=2 | 9 | 52 | 22 | 365 | 41 | 116 | 155 | 41 | 0.41 | 0.83 |
| A3KLR9 | Superoxide dismutase [Cu-Zn] OS=Bos taurus SOD3 PE=1 SV=1 | 19 | 158 | 63 | 315 | 24 | 137 | 21 | 63 | 0.40 | 0.28 |
| P07224 | Vitamin K-dependent protein S OS=Bos taurus PROS1 PE=1 SV=1 | 31 | 158 | 50 | 75 | 160 | 91 | 87 | 148 | 1.33 | 0.37 |
| F1N4M7 | Complement factor I OS=Bos taurus CFI PE=1 SV=2 | 42 | 81 | 52 | 97 | 154 | 117 | 139 | 119 | 1.86 | 0.06 |
| E1BF48 | CD177 molecule OS=Bos taurus CD177 PE=1 SV=2 | . | 43 | 12 | 408 | 5 | 126 | 153 | 54 | 0.50 | 0.81 |
| F1MSE7 | Sushi domain containing 2 OS=Bos taurus SUSD2 PE=4 SV=1 | 49 | 164 | 114 | 173 | 33 | 134 | 29 | 105 | 0.48 | 0.05 |
| Q3MHN2 | Complement component C9 OS=Bos taurus C9 PE=2 SV=1 | 67 | 57 | 124 | 54 | 129 | 149 | 131 | 89 | 1.64 | 0.07 |
| A0A140T8C6 | Prosaposin OS=Bos taurus PSAP PE=4 SV=1 | 50 | 224 | 100 | 181 | 38 | 88 | 41 | 79 | 0.46 | 0.06 |
| E1B8B5 | Uncharacterized protein OS=Bos taurus OX=9913 PE=3 SV=2 | . | 21 | . | 362 | . | 106 | 262 | 49 | . | 0.67 |
| Q32LE5 | Isoaspartyl peptidase/L-asparaginase OS=Bos taurus ASRGL1 PE=2 SV=1 | 137 | 84 | 86 | 130 | 76 | 104 | 89 | 94 | 0.81 | 0.39 |
| Q3SYV4 | Adenylyl cyclase-associated protein 1 OS=Bos taurus CAP1 PE=2 SV=3 | 68 | 88 | 129 | 167 | 43 | 158 | 83 | 64 | 0.64 | 0.52 |
| A0A0A0MP89 | Serpin A3-8 OS=Bos taurus SERPINA3-8 PE=3 SV=1 | 28 | 55 | 21 | 113 | 275 | 88 | 148 | 72 | 1.64 | 0.24 |
| P56425 | Cathelicidin-7 OS=Bos taurus CATHL7 PE=2 SV=1 | 4 | 73 | 19 | 484 | 15 | 109 | 58 | 39 | 0.29 | 0.50 |
| F1MNW4 | Inter-alpha-trypsin inhibitor heavy chain H2 OS=Bos taurus ITIH2 PE=1 SV=2 | 26 | 49 | 14 | 32 | 399 | 89 | 120 | 71 | 3.35 | 0.18 |
| Q0VCK0 | Bifunctional purine biosynthesis protein PURH OS=Bos taurus ATIC PE=2 SV=1 | 153 | 75 | 140 | 75 | 108 | 88 | 87 | 76 | 0.82 | 0.29 |
| P33433 | Histidine-rich glycoprotein (Fragments) OS=Bos taurus HRG PE=1 SV=1 | 51 | 105 | 52 | 140 | 55 | 157 | 147 | 93 | 1.16 | 0.45 |
| Q27970 | Calpain-1 catalytic subunit OS=Bos taurus CAPN1 PE=1 SV=3 | 104 | 101 | 89 | 103 | 135 | 103 | 87 | 79 | 0.98 | 0.88 |
| P52556 | Flavin reductase (NADPH) OS=Bos taurus BLVRB PE=1 SV=2 | 248 | 61 | 239 | 44 | 19 | 63 | 84 | 42 | 0.23 | 0.19 |
| E1BCS3 | Hexokinase 3 OS=Bos taurus HK3 PE=1 SV=1 | 39 | 74 | 230 | 119 | 69 | 108 | 57 | 105 | 0.62 | 0.57 |
| A5D7K0 | Biliverdin reductase A OS=Bos taurus BLVRA PE=2 SV=1 | 250 | 71 | 213 | 60 | 40 | 61 | 50 | 55 | 0.31 | 0.16 |
| Q2KIW9 | UMP-CMP kinase OS=Bos taurus CMPK1 PE=2 SV=2 | 179 | 102 | 97 | 82 | 56 | 74 | 107 | 105 | 0.64 | 0.44 |
| G3MXD9 | Uncharacterized protein OS=Bos taurus OX=9913 PE=4 SV=1 | 16 | 100 | 46 | 242 | 105 | 66 | 78 | 146 | 1.03 | 0.96 |
| G3N3Q3 | Uncharacterized protein OS=Bos taurus OX=9913 PE=4 SV=1 | 34 | 106 | 15 | 202 | 90 | 174 | 136 | 44 | 0.70 | 0.75 |
| Q6EWQ7 | Eukaryotic translation initiation factor 5A-1 OS=Bos taurus EIF5A PE=2 SV=3 | 180 | 51 | 161 | 87 | 119 | 36 | 48 | 119 | 0.57 | 0.30 |
| A6QP39 | MSLN protein OS=Bos taurus MSLN PE=2 SV=1 | 31 | 36 | 47 | 388 | 33 | 50 | 129 | 87 | 0.62 | 0.59 |
| E1BMJ0 | Factor XIIa inhibitor precursor OS=Bos taurus SERPING1 PE=3 SV=2 | 25 | 106 | 93 | 111 | 98 | 215 | 82 | 70 | 1.15 | 0.43 |
| G3X6M2 | Uncharacterized protein OS=Bos taurus CD55 PE=4 SV=1 | 70 | 175 | 53 | 148 | 51 | 82 | 48 | 173 | 0.73 | 0.43 |
| Q3ZC44 | Heterogeneous nuclear ribonucleoprotein A/B OS=Bos taurus HNRNPAB PE=1 SV=1 | 86 | 105 | 135 | 140 | 58 | 94 | 95 | 87 | 0.71 | 0.03 |
| Q2KIU3 | Protein HP-25 homolog 2 OS=Bos taurus OX=9913 PE=2 SV=1 | 41 | 128 | 42 | 103 | 119 | 173 | 110 | 84 | 1.49 | 0.14 |
| F1MF86 | Latent-transforming growth factor beta-binding protein 2 OS=Bos taurus LTBP2 PE=4 SV=1 | 112 | 172 | 65 | 167 | . | 113 | 39 | 131 | 0.62 | 0.05 |
| A7YY28 | Protein ABHD14B OS=Bos taurus ABHD14B PE=2 SV=1 | 181 | 105 | 226 | 63 | 17 | 97 | 62 | 49 | 0.24 | 0.14 |
| P79136 | F-actin-capping protein subunit beta OS=Bos taurus CAPZB PE=1 SV=1 | 90 | 86 | 65 | 146 | 59 | 91 | 122 | 141 | 0.99 | 0.75 |
| F1N0R8 | L-serine dehydratase/L-threonine deaminase OS=Bos taurus SDS PE=4 SV=1 | 4 | 55 | 37 | 135 | 158 | 315 | 74 | 22 | 0.58 | 0.37 |
| P85521 | Scavenger receptor cysteine-rich type 1 protein M130 OS=Bos taurus CD163 PE=1 SV=2 | 36 | 184 | 87 | 189 | 27 | 96 | 48 | 134 | 0.61 | 0.06 |
| Q3T054 | GTP-binding nuclear protein Ran OS=Bos taurus RAN PE=2 SV=3 | 205 | 66 | 142 | 61 | 102 | 91 | 87 | 45 | 0.70 | 0.26 |
| Q3SZ54 | Eukaryotic initiation factor 4A-I OS=Bos taurus EIF4A1 PE=2 SV=1 | 95 | 71 | 118 | 121 | 62 | 118 | 144 | 70 | 0.86 | 0.92 |
| A4IFC3 | Polyadenylate-binding protein OS=Bos taurus PABPC4 PE=2 SV=1 | 80 | 112 | 119 | 109 | 73 | 170 | 79 | 59 | 0.78 | 0.72 |
| P61286 | Polyadenylate-binding protein 1 OS=Bos taurus PABPC1 PE=2 SV=1 | 34 | 108 | 97 | 92 | 105 | 172 | 96 | 96 | 1.38 | 0.16 |
| Q28046 | Adseverin OS=Bos taurus SCIN PE=1 SV=1 | 163 | 87 | 55 | 83 | 74 | 133 | 133 | 71 | 0.90 | 0.88 |
| A8YXX7 | Trefoil factor 3 OS=Bos taurus TFF3 PE=3 SV=1 | 56 | 151 | 55 | 202 | . | 60 | 57 | 218 | 0.90 | 0.54 |
| P11116 | Galectin-1 OS=Bos taurus LGALS1 PE=1 SV=2 | 92 | 111 | 132 | 160 | 10 | 92 | 88 | 115 | 0.29 | 0.04 |
| F1MU79 | Peptidylprolyl isomerase OS=Bos taurus FKBP4 PE=4 SV=2 | 170 | 89 | 127 | 70 | 44 | 123 | 96 | 81 | 0.59 | 0.49 |
| E1BE76 | Uncharacterized protein OS=Bos taurus TPD52 PE=4 SV=2 | 194 | 75 | 210 | 70 | 72 | 86 | 53 | 40 | 0.43 | 0.15 |
| Q2KIW1 | Paraoxonase 1 OS=Bos taurus PON1 PE=1 SV=1 | 69 | 104 | 200 | 59 | 123 | 87 | 62 | 95 | 0.72 | 0.74 |
| E1BAI4 | Chloride intracellular channel protein OS=Bos taurus CLIC6 PE=3 SV=1 | 113 | 92 | 89 | 89 | 96 | 55 | 63 | 204 | 0.85 | 0.82 |
| F1N3V0 | Malic enzyme OS=Bos taurus ME1 PE=3 SV=2 | 224 | 77 | 235 | 37 | 63 | 85 | 39 | 39 | 0.35 | 0.20 |
| Q32L99 | Prostaglandin reductase 2 OS=Bos taurus PTGR2 PE=2 SV=1 | 210 | 78 | 265 | 39 | 41 | 62 | 50 | 55 | 0.32 | 0.19 |
| Q6QRN6 | NAD(P)H-hydrate epimerase OS=Bos taurus NAXE PE=2 SV=1 | 134 | 79 | 162 | 67 | 58 | 82 | 117 | 100 | 0.75 | 0.45 |
| F1MMP5 | Inter-alpha-trypsin inhibitor heavy chain H1 OS=Bos taurus ITIH1 PE=4 SV=1 | 41 | 71 | 28 | 91 | 309 | 90 | 91 | 79 | 1.69 | 0.27 |
| G5E537 | Uncharacterized protein OS=Bos taurus OX=9913 PE=3 SV=1 |  | 19 | . | 424 | 55 | 105 | 184 | 13 | . | 0.63 |
| Q2YDE4 | Proteasome subunit alpha type-6 OS=Bos taurus PSMA6 PE=1 SV=1 | 153 | 105 | 153 | 99 | 64 | 69 | 74 | 83 | 0.56 | 0.05 |
| F1MJQ3 | Alpha-amylase OS=Bos taurus AMY2B PE=3 SV=1 | 53 | 129 | 25 | 76 | 139 | 135 | 167 | 76 | 1.61 | 0.18 |
| E1BP91 | Aminopeptidase OS=Bos taurus NPEPPS PE=1 SV=2 | 139 | 84 | 198 | 70 | 60 | 74 | 86 | 89 | 0.61 | 0.23 |
| P00794 | Chymosin OS=Bos taurus CYM PE=1 SV=3 | 21 | 154 | 111 | 146 | 105 | 69 | 43 | 151 | 0.67 | 0.71 |
| P61585 | Transforming protein RhoA OS=Bos taurus RHOA PE=1 SV=1 | 228 | 64 | 172 | 118 | 22 | 73 | 67 | 57 | 0.25 | 0.14 |
| Q8SPU5 | BPI fold-containing family A member 1 OS=Bos taurus BPIFA1 PE=2 SV=1 | 20 | 82 | 58 | 301 | 9 | 111 | 65 | 154 | 0.69 | 0.49 |
| G3MWP1 | Elastase, neutrophil expressed OS=Bos taurus ELANE PE=1 SV=1 | 6 | 52 | 23 | 401 | 22 | 129 | 133 | 34 | 0.32 | 0.73 |
| F1N2A2 | Serpin B6 OS=Bos taurus SERPINB6 PE=3 SV=2 | 140 | 121 | 150 | 88 | 58 | 115 | 58 | 70 | 0.55 | 0.11 |
| Q5KR47 | Tropomyosin alpha-3 chain OS=Bos taurus TPM3 PE=2 SV=1 | 115 | 49 | 90 | 112 | 254 | 55 | 68 | 58 | 0.87 | 0.71 |
| P60661 | Myosin light polypeptide 6 OS=Bos taurus MYL6 PE=2 SV=2 | 198 | 79 | 137 | 76 | 60 | 87 | 85 | 78 | 0.59 | 0.28 |
| G3MXK8 | Proteinase 3 OS=Bos taurus PRTN3 PE=1 SV=1 | 4 | 42 | 24 | 450 | 10 | 164 | 86 | 21 | 0.18 | 0.67 |
| A6H7J6 | Protein disulfide-isomerase OS=Bos taurus P4HB PE=1 SV=1 | 65 | 135 | 99 | 142 | 59 | 83 | 105 | 112 | 0.81 | 0.20 |
| Q9MZ08 | Basal cell adhesion molecule OS=Bos taurus BCAM PE=2 SV=2 | 44 | 174 | 90 | 140 | 101 | 86 | 45 | 119 | 0.71 | 0.48 |
| Q92176 | Coronin-1A OS=Bos taurus CORO1A PE=1 SV=3 | 15 | 54 | 60 | 251 | 44 | 126 | 144 | 106 | 1.12 | 0.87 |
| Q9GMB8 | Serine--tRNA ligase, cytoplasmic OS=Bos taurus SARS PE=2 SV=3 | 105 | 84 | 134 | 126 | 31 | 101 | 109 | 110 | 0.60 | 0.28 |
| A0A140T8C5 | Uteroglobin OS=Bos taurus SCGB1A1 PE=4 SV=1 | 89 | 54 | 52 | 262 | 8 | 24 | 38 | 274 | 0.25 | 0.24 |
| P19803 | Rho GDP-dissociation inhibitor 1 OS=Bos taurus ARHGDIA PE=1 SV=3 | 265 | 61 | 160 | 89 | 28 | 58 | 72 | 68 | 0.28 | 0.20 |
| P68509 | 14-3-3 protein eta OS=Bos taurus YWHAH PE=1 SV=2 | 207 | 80 | 173 | 70 | 13 | 61 | 87 | 110 | 0.20 | 0.29 |
| P13135 | Calpain small subunit 1 OS=Bos taurus CAPNS1 PE=2 SV=1 | 245 | 121 | 153 | 82 | 31 | 47 | 54 | 67 | 0.27 | 0.10 |
| P07107 | Acyl-CoA-binding protein OS=Bos taurus DBI PE=1 SV=2 | 204 | 94 | 194 | 60 | 44 | 59 | 61 | 83 | 0.40 | 0.17 |
| G3MX65 | WAP four-disulfide core domain 2 OS=Bos taurus WFDC2 PE=1 SV=1 | 45 | 152 | 96 | 204 | . | 74 | 24 | 205 | 0.56 | 0.19 |
| F1MM13 | Hexose-6-phosphate dehydrogenase/glucose 1-dehydrogenase OS=Bos taurus H6PD PE=1 SV=2 | 39 | 195 | 55 | 123 | 99 | 97 | 67 | 124 | 0.95 | 0.86 |
| Q28034 | Glucosidase 2 subunit beta OS=Bos taurus PRKCSH PE=2 SV=1 | 56 | 174 | 94 | 145 | 45 | 84 | 56 | 146 | 0.67 | 0.18 |
| F1MGZ5 | Chloride channel accessory 1 OS=Bos taurus CLCA1 PE=1 SV=2 | 46 | 40 | 153 | 288 | 14 | 17 | 32 | 211 | 0.34 | 0.07 |
| F1MKH8 | 72 kDa type IV collagenase OS=Bos taurus MMP2 PE=3 SV=1 | 35 | 162 | 160 | 81 | 81 | 97 | 37 | 148 | 0.57 | 0.71 |
| G5E5C8 | Transaldolase OS=Bos taurus TALDO1 PE=1 SV=1 | 108 | 86 | 127 | 143 | 24 | 93 | 118 | 103 | 0.50 | 0.22 |
| Q0P5K3 | Ubiquitin-conjugating enzyme E2 N OS=Bos taurus UBE2N PE=2 SV=1 | 142 | 98 | 135 | 94 | 57 | 85 | 101 | 88 | 0.66 | 0.15 |
| F1N4T5 | Glutathione S-transferase A4 OS=Bos taurus GSTA3 PE=4 SV=2 | 37 | 31 | 194 | 86 | 9 | 32 | 92 | 319 | 0.53 | 0.75 |
| Q0P569 | Nucleobindin-1 OS=Bos taurus NUCB1 PE=2 SV=1 | 64 | 97 | 67 | 180 | 92 | 87 | 75 | 138 | 1.00 | 0.81 |
| A5PKH3 | Fumarylacetoacetase OS=Bos taurus FAH PE=2 SV=1 | 77 | 117 | 74 | 110 | 77 | 106 | 107 | 132 | 1.10 | 0.37 |
| P00921 | Carbonic anhydrase 2 OS=Bos taurus CA2 PE=1 SV=3 | 113 | 38 | 22 | 72 | . | 124 | 371 | 61 | 2.57 | 0.32 |
| P00435 | Glutathione peroxidase 1 OS=Bos taurus GPX1 PE=1 SV=3 | 168 | 71 | 121 | 109 | 61 | 83 | 108 | 80 | 0.66 | 0.27 |
| Q3B7M5 | LIM and SH3 domain protein 1 OS=Bos taurus LASP1 PE=2 SV=1 | 185 | 64 | 188 | 84 | 66 | 83 | 61 | 71 | 0.51 | 0.21 |
| F1MZ33 | Deoxyribonuclease-2-alpha OS=Bos taurus DNASE2 PE=4 SV=2 | 40 | 129 | 66 | 113 | 104 | 91 | 105 | 153 | 1.26 | 0.32 |
| P33097 | Aspartate aminotransferase, cytoplasmic OS=Bos taurus GOT1 PE=1 SV=3 | 223 | 79 | 221 | 57 | 9 | 59 | 75 | 77 | 0.14 | 0.19 |
| A5D7E8 | Protein disulfide-isomerase OS=Bos taurus PDIA3 PE=2 SV=1 | 109 | 98 | 222 | 84 | 11 | 52 | 76 | 148 | 0.27 | 0.30 |
| Q3Y5Z3 | Adiponectin OS=Bos taurus ADIPOQ PE=1 SV=1 | 48 | 28 | 12 | 200 | 155 | 143 | 134 | 81 | 1.31 | 0.41 |
| F1MX83 | Protein S100 OS=Bos taurus S100A11 PE=1 SV=2 | 126 | 67 | 72 | 131 | 21 | 91 | 162 | 130 | 0.48 | 0.96 |
| Q5E9D5 | Destrin OS=Bos taurus DSTN PE=2 SV=3 | 225 | 71 | 196 | 35 | 53 | 74 | 73 | 72 | 0.48 | 0.29 |
| Q9TU25 | Ras-related C3 botulinum toxin substrate 2 OS=Bos taurus RAC2 PE=2 SV=1 | 182 | 57 | 129 | 126 | 40 | 79 | 122 | 65 | 0.48 | 0.29 |
| Q3ZCJ2 | Alcohol dehydrogenase [NADP(+)] OS=Bos taurus AKR1A1 PE=2 SV=1 | 114 | 113 | 171 | 97 | 71 | 73 | 67 | 95 | 0.60 | 0.11 |
| P00727 | Cytosol aminopeptidase OS=Bos taurus LAP3 PE=1 SV=3 | 117 | 65 | 112 | 73 | 195 | 98 | 99 | 42 | 0.97 | 0.53 |
| P08169 | Cation-independent mannose-6-phosphate receptor OS=Bos taurus IGF2R PE=1 SV=2 | 44 | 131 | 35 | 61 | 144 | 81 | 107 | 199 | 1.57 | 0.21 |
| E1BLA8 | Golgi membrane protein 1 OS=Bos taurus GOLM1 PE=4 SV=1 | 33 | 227 | 65 | 85 | 41 | 190 | 55 | 105 | 1.00 | 0.72 |
| Q762I5 | Resistin OS=Bos taurus RETN PE=3 SV=1 | 5 | 40 | 35 | 369 | 41 | 124 | 106 | 80 | 0.74 | 0.80 |
| P28800 | Alpha-2-antiplasmin OS=Bos taurus SERPINF2 PE=1 SV=2 | 23 | 40 | 18 | 34 | 392 | 79 | 146 | 68 | 3.39 | 0.17 |
| E1BEG2 | Heterogeneous nuclear ribonucleoprotein A3 OS=Bos taurus HNRNPA3 PE=1 SV=2 | 97 | 93 | 105 | 172 | 107 | 42 | 65 | 119 | 0.65 | 0.11 |
| P46162 | Beta-defensin 4 OS=Bos taurus DEFB4 PE=1 SV=3 | 25 | 75 | 42 | 216 | 62 | 138 | 150 | 91 | 1.11 | 0.71 |
| A0A140T881 | Apolipoprotein E OS=Bos taurus APOE PE=3 SV=1 | 44 | 115 | 46 | 58 | 236 | 86 | 126 | 90 | 1.58 | 0.24 |
| E1BFG0 | Alpha-aminoadipic semialdehyde dehydrogenase OS=Bos taurus ALDH7A1 PE=3 SV=2 | 117 | 68 | 141 | 106 | 25 | 151 | 76 | 117 | 0.51 | 0.72 |
| F1N1Z8 | Uncharacterized protein OS=Bos taurus OX=9913 PE=4 SV=2 | 20 | 96 | 49 | 495 | 27 | 33 | 33 | 47 | 0.26 | 0.31 |
| F1N2L9 | 4-trimethylaminobutyraldehyde dehydrogenase OS=Bos taurus ALDH9A1 PE=3 SV=2 | 111 | 81 | 124 | 188 | 44 | 71 | 86 | 96 | 0.57 | 0.06 |
| F1MG05 | Elongation factor 1-gamma OS=Bos taurus EEF1G PE=4 SV=1 | 99 | 120 | 138 | 148 | 28 | 102 | 84 | 82 | 0.49 | 0.02 |
| O97764 | Zeta-crystallin OS=Bos taurus CRYZ PE=2 SV=2 | 217 | 85 | 173 | 59 | 63 | 81 | 63 | 61 | 0.49 | 0.18 |
| M0QVY0 | Keratin, type II cytoskeletal 6A OS=Bos taurus KRT6C PE=3 SV=1 | 150 | . | 117 | 382 | . | 29 | 80 | 43 | . | 0.43 |
| A5D7J6 | CALR protein OS=Bos taurus CALR PE=2 SV=1 | 71 | 151 | 109 | 113 | 39 | 63 | 93 | 162 | 0.66 | 0.50 |
| Q2VPS3 | Uteroglobin OS=Bos taurus SCGB1A1 PE=3 SV=1 | 30 | 380 | 27 | 147 | 16 | 68 | 29 | 105 | 0.41 | 0.30 |
| G3N1R1 | Uncharacterized protein OS=Bos taurus OX=9913 PE=4 SV=1 | 37 | 113 | 49 | 72 | 44 | 116 | 113 | 256 | 1.58 | 0.22 |
| E1BCL5 | Uncharacterized protein OS=Bos taurus OX=9913 PE=3 SV=1 | 90 | 122 | 106 | 140 | 106 | 99 | 86 | 52 | 0.66 | 0.28 |
| P80189 | Lysozyme C, non-stomach isozyme OS=Bos taurus LYS PE=2 SV=4 | 42 | 142 | 69 | 138 | 68 | 115 | 63 | 164 | 1.05 | 0.75 |
| F1MSI2 | Agrin OS=Bos taurus AGRN PE=1 SV=1 | 58 | 262 | 88 | 117 | . | 112 | 39 | 124 | 0.72 | 0.30 |
| Q3ZCH5 | Zinc-alpha-2-glycoprotein OS=Bos taurus AZGP1 PE=2 SV=1 | 187 | 102 | 133 | 79 | 32 | 75 | 78 | 116 | 0.42 | 0.30 |
| P19858 | L-lactate dehydrogenase A chain OS=Bos taurus LDHA PE=2 SV=2 | 57 | 120 | 88 | 123 | 129 | 94 | 106 | 83 | 1.00 | 0.82 |
| Q3T010 | Phosphatidylethanolamine-binding protein 4 OS=Bos taurus PEBP4 PE=2 SV=1 | 48 | 229 | 85 | 164 | 7 | 81 | 31 | 155 | 0.30 | 0.13 |
| F1N3P2 | Ubiquitin carboxyl-terminal hydrolase OS=Bos taurus USP5 PE=1 SV=2 | 141 | 101 | 93 | 148 | 29 | 101 | 107 | 80 | 0.47 | 0.26 |
| F1MHQ4 | Fatty acid-binding protein, adipocyte OS=Bos taurus FABP4 PE=3 SV=2 | 30 | 219 | 101 | 94 | . | 94 | 24 | 238 | 0.58 | 0.84 |
| F1MIH4 | Chloride intracellular channel protein OS=Bos taurus CLIC5 PE=3 SV=1 | 35 | 105 | 34 | 89 | 100 | 215 | 143 | 79 | 1.83 | 0.09 |
| A6QLZ0 | Galectin OS=Bos taurus LGALS3 PE=1 SV=1 | 154 | 104 | 149 | 85 | 65 | 118 | 57 | 69 | 0.56 | 0.19 |
| Q3SYT9 | Poly(RC) binding protein 2 OS=Bos taurus PCBP2 PE=1 SV=1 | 154 | 81 | 121 | 100 | 74 | 121 | 84 | 66 | 0.70 | 0.35 |
| E1BCU6 | Transcobalamin 1 OS=Bos taurus TCN1 PE=1 SV=1 | 6 | 32 | 5 | 186 | 47 | 306 | 142 | 75 | 1.46 | 0.37 |
| Q58DU5 | Proteasome subunit alpha type-3 OS=Bos taurus PSMA3 PE=1 SV=3 | 124 | 100 | 180 | 129 | 40 | 83 | 61 | 84 | 0.45 | 0.06 |
| Q3ZCK3 | 3'(2'),5'-bisphosphate nucleotidase 1 OS=Bos taurus BPNT1 PE=2 SV=1 | 127 | 110 | 140 | 48 | 48 | 119 | 108 | 101 | 0.75 | 0.68 |
| E1B773 | Uncharacterized protein OS=Bos taurus OX=9913 PE=4 SV=2 | 195 | 67 | 145 | 71 | 54 | 63 | 98 | 107 | 0.59 | 0.39 |
| A0A0A0MP90 | Histone H2A OS=Bos taurus HIST1H2AC PE=3 SV=1 | 27 | 88 | 56 | 337 | 56 | 103 | 97 | 36 | 0.35 | 0.56 |
| Q2KJ93 | Cell division control protein 42 homolog OS=Bos taurus CDC42 PE=1 SV=1 | 191 | 67 | 141 | 88 | 56 | 103 | 94 | 59 | 0.57 | 0.31 |
| A7MAZ5 | Histone H1.3 OS=Bos taurus HIST1H1D PE=1 SV=1 | 35 | 85 | 147 | 247 | 54 | 125 | 63 | 44 | 0.43 | 0.38 |
| Q3MHY1 | Cysteine and glycine-rich protein 1 OS=Bos taurus CSRP1 PE=2 SV=3 | 281 | 59 | 300 | 25 | 71 | 31 | 15 | 18 | 0.15 | 0.15 |
| Q3MI05 | Lysosomal protective protein OS=Bos taurus CTSA PE=2 SV=1 | 37 | 101 | 72 | 86 | 51 | 184 | 88 | 182 | 1.55 | 0.10 |
| Q0IIM3 | Heat shock protein 105 kDa OS=Bos taurus HSPH1 PE=2 SV=1 | 95 | 119 | 132 | 122 | 32 | 107 | 99 | 95 | 0.59 | 0.05 |
| A6H7E3 | PDZ and LIM domain 1 OS=Bos taurus PDLIM1 PE=1 SV=1 | 167 | 86 | 143 | 75 | 97 | 89 | 74 | 71 | 0.70 | 0.18 |
| P18203 | Peptidyl-prolyl cis-trans isomerase FKBP1A OS=Bos taurus FKBP1A PE=1 SV=2 | 168 | 79 | 256 | 80 | 15 | 96 | 49 | 56 | 0.22 | 0.18 |
| E1B805 | Uncharacterized protein OS=Bos taurus LOC528040 PE=4 SV=2 | 39 | 124 | 36 | 66 | 146 | 147 | 167 | 76 | 1.82 | 0.11 |
| F1MDH3 | Talin 1 OS=Bos taurus TLN1 PE=1 SV=2 | 76 | 76 | 117 | 145 | 141 | 77 | 74 | 94 | 0.86 | 0.81 |
| O02675 | Dihydropyrimidinase-related protein 2 OS=Bos taurus DPYSL2 PE=1 SV=1 | 269 | 92 | 271 | 46 | 29 | 32 | 29 | 31 | 0.17 | 0.10 |
| Q05204 | Lysosome-associated membrane glycoprotein 1 OS=Bos taurus LAMP1 PE=1 SV=2 | 70 | 91 | 72 | 76 | 176 | 120 | 90 | 105 | 1.50 | 0.11 |
| H9KUV2 | S-methyl-5'-thioadenosine phosphorylase OS=Bos taurus MTAP PE=3 SV=1 | 192 | 71 | 121 | 71 | 56 | 86 | 121 | 82 | 0.65 | 0.50 |
| A5PJH7 | LOC788112 protein OS=Bos taurus LOC788112 PE=1 SV=1 | . | 35 | 26 | 244 | 27 | 292 | 125 | 50 | 0.77 | 0.72 |
| Q9TU03 | Rho GDP-dissociation inhibitor 2 OS=Bos taurus ARHGDIB PE=2 SV=3 | 13 | 49 | 35 | 431 | 18 | 80 | 104 | 71 | 0.52 | 0.57 |
| P56965 | N(G),N(G)-dimethylarginine dimethylaminohydrolase 1 OS=Bos taurus DDAH1 PE=1 SV=3 | 81 | 75 | 105 | 76 | 42 | 101 | 220 | 101 | 1.02 | 0.39 |
| E1BJW6 | Napsin A aspartic peptidase OS=Bos taurus NAPSA PE=3 SV=2 | 57 | 254 | 103 | 123 | 18 | 86 | 36 | 123 | 0.40 | 0.15 |
| P55052 | Fatty acid-binding protein 5 OS=Bos taurus FABP5 PE=1 SV=4 | 46 | 128 | 102 | 227 | 7 | 97 | 72 | 120 | 0.36 | 0.07 |
| G3MZJ9 | Uncharacterized protein OS=Bos taurus LOC100850808 PE=4 SV=1 | 23 | 80 | 47 | 473 | 22 | 86 | 33 | 36 | 0.24 | 0.38 |
| Q1JP75 | L-xylulose reductase OS=Bos taurus DCXR PE=2 SV=1 | 122 | 135 | 69 | 131 | . | 149 | 101 | 94 | 1.34 | 0.90 |
| Q3ZBL4 | Leucine zipper transcription factor-like protein 1 OS=Bos taurus LZTFL1 PE=2 SV=1 | 115 | 96 | 106 | 98 | 126 | 75 | 89 | 96 | 0.91 | 0.42 |
| Q32KP9 | Nuclear transport factor 2 OS=Bos taurus NUTF2 PE=2 SV=1 | 224 | 72 | 117 | 65 | 61 | 68 | 105 | 88 | 0.61 | 0.42 |
| F1MQT9 | CD44 antigen OS=Bos taurus CD44 PE=4 SV=2 | 64 | 172 | 62 | 121 | 24 | 79 | 72 | 206 | 0.64 | 0.82 |
| A6QR15 | LOC535277 protein OS=Bos taurus TPM4 PE=1 SV=1 | 89 | 145 | 86 | 78 | 213 | 52 | 30 | 108 | 0.59 | 0.98 |
| G3N0S9 | Uncharacterized protein OS=Bos taurus LOC515150 PE=4 SV=1 | 75 | 99 | 69 | 126 | 76 | 72 | 89 | 195 | 1.05 | 0.50 |
| P10462 | Protein S100-A2 OS=Bos taurus S100A2 PE=1 SV=1 | 282 | 49 | 236 | 56 | 48 | 44 | 42 | 43 | 0.29 | 0.16 |
| F1MCN3 | CD97 antigen precursor OS=Bos taurus ADGRE5 PE=3 SV=1 | 77 | 174 | 28 | 107 | 73 | 107 | 56 | 179 | 1.06 | 0.82 |
| Q5E984 | Translationally-controlled tumor protein OS=Bos taurus TPT1 PE=2 SV=1 | 106 | 71 | 112 | 99 | 57 | 107 | 133 | 115 | 0.95 | 0.77 |
| Q0V7N2 | Complement C2 OS=Bos taurus C2 PE=2 SV=1 | 33 | 174 | 31 | 103 | 214 | 88 | 72 | 85 | 1.06 | 0.64 |
| A6QQN6 | Acid sphingomyelinase-like phosphodiesterase OS=Bos taurus SMPDL3B PE=2 SV=1 | 55 | 203 | 108 | 102 | 34 | 125 | 34 | 141 | 0.56 | 0.31 |
| F1MR63 | Uncharacterized protein OS=Bos taurus ABHD14A PE=1 SV=2 | 200 | 66 | 173 | 66 | 81 | 75 | 75 | 65 | 0.60 | 0.21 |
| G3N3D4 | Potassium channel tetramerization domain containing 12 OS=Bos taurus KCTD12 PE=1 SV=1 | 162 | 110 | 59 | 118 | 32 | 122 | 128 | 69 | 0.49 | 0.60 |
| Q2HJ60 | Heterogeneous nuclear ribonucleoproteins A2/B1 OS=Bos taurus HNRNPA2B1 PE=2 SV=1 | 72 | 64 | 81 | 261 | . | 77 | 123 | 122 | 1.10 | 0.67 |
| P00741 | Coagulation factor IX OS=Bos taurus F9 PE=1 SV=2 | 93 | 83 | 108 | 65 | 70 | 100 | 183 | 98 | 1.17 | 0.30 |
| P07688 | Cathepsin B OS=Bos taurus CTSB PE=1 SV=5 | 32 | 103 | 52 | 120 | 22 | 156 | 149 | 167 | 1.25 | 0.12 |
| A5PK96 | ACP1 protein OS=Bos taurus ACP1 PE=2 SV=1 | 203 | 74 | 168 | 51 | 59 | 71 | 86 | 88 | 0.57 | 0.32 |
| P11019 | V-type proton ATPase subunit E 1 OS=Bos taurus ATP6V1E1 PE=2 SV=1 | 89 | 54 | 115 | 46 | 327 | 72 | 46 | 52 | 0.91 | 0.52 |
| P84080 | ADP-ribosylation factor 1 OS=Bos taurus ARF1 PE=1 SV=2 | 139 | 93 | 254 | 73 | 45 | 101 | 38 | 56 | 0.34 | 0.21 |
| G3MZ19 | HRPE773-like OS=Bos taurus ZG16B PE=4 SV=1 | 22 | 116 | 52 | 518 | 21 | 39 | 14 | 17 | 0.11 | 0.28 |
| E1BFL8 | Interleukin 1 receptor accessory protein OS=Bos taurus IL1RAP PE=4 SV=2 | 42 | 133 | 42 | 96 | 208 | 98 | 82 | 101 | 1.32 | 0.39 |
| Q3T0Q4 | Nucleoside diphosphate kinase B OS=Bos taurus NME2 PE=1 SV=1 | 144 | 74 | 138 | 106 | 54 | 107 | 101 | 76 | 0.65 | 0.30 |
| Q2HJH3 | Ester hydrolase C11orf54 homolog OS=Bos taurus OX=9913 PE=2 SV=1 | 227 | 81 | 201 | 48 | 45 | 60 | 65 | 74 | 0.39 | 0.20 |
| F1N2R1 | Stomatin OS=Bos taurus STOM PE=1 SV=2 | 113 | 73 | 119 | 249 | . | 89 | 74 | 84 | 0.74 | 0.35 |
| F1MK52 | Prominin 1 OS=Bos taurus PROM1 PE=4 SV=2 | 8 | 101 | 73 | 400 | 14 | 46 | 34 | 123 | 0.49 | 0.24 |
| A6QNL0 | Monocyte differentiation antigen CD14 OS=Bos taurus CD14 PE=2 SV=1 | 41 | 138 | 54 | 117 | 47 | 132 | 120 | 150 | 1.28 | 0.21 |
| F1MW44 | Coagulation factor XIII A chain OS=Bos taurus F13A1 PE=4 SV=1 | . | . | . | 71 | 609 | 45 | 60 | 14 | . | #DIV/0! |
| F1MC48 | IQ motif containing GTPase activating protein 1 OS=Bos taurus IQGAP1 PE=1 SV=2 | 150 | 113 | 122 | 128 | 98 | 61 | 63 | 64 | 0.55 | 0.00 |
| P22444 | Bone morphogenetic protein 3 OS=Bos taurus BMP3 PE=1 SV=2 | 47 | 171 | 52 | 158 | 27 | 66 | 52 | 227 | 0.66 | 0.72 |
| F1MW03 | Thiosulfate sulfurtransferase like domain containing 1 OS=Bos taurus TSTD1 PE=1 SV=2 | 115 | 80 | 235 | 58 | 73 | 79 | 43 | 117 | 0.47 | 0.47 |
| F1MHN8 | CD166 antigen OS=Bos taurus ALCAM PE=4 SV=2 | 46 | 233 | 67 | 84 | 27 | 63 | 38 | 243 | 0.53 | 0.84 |
| Q2NL00 | Glutathione S-transferase theta-1 OS=Bos taurus GSTT1 PE=2 SV=3 | 207 | 81 | 188 | 80 | 26 | 72 | 96 | 50 | 0.32 | 0.14 |
| Q3T0Z7 | Dihydropteridine reductase OS=Bos taurus QDPR PE=2 SV=1 | 210 | 72 | 164 | 52 | 45 | 89 | 86 | 82 | 0.50 | 0.36 |
| Q3SYR0 | Serpin peptidase inhibitor, clade A (Alpha-1 antiproteinase, antitrypsin), member 7 OS=Bos taurus SERPINA7 PE=2 SV=1 | 29 | 96 | 37 | 57 | 345 | 94 | 85 | 58 | 1.59 | 0.32 |
| E1BMX5 | Neuropilin OS=Bos taurus NRP1 PE=3 SV=1 | 67 | 236 | 75 | 91 | 31 | 46 | 35 | 219 | 0.41 | 0.63 |
| G3MXL3 | Keratin 3 OS=Bos taurus KRT3 PE=1 SV=1 | . | . | 80 | . | . | . | 169 | 551 | . | #DIV/0! |
| E1BHY6 | Granulin precursor OS=Bos taurus GRN PE=4 SV=2 | 43 | 147 | 207 | 148 | 42 | 133 | 34 | 46 | 0.35 | 0.17 |
| P01888 | Beta-2-microglobulin OS=Bos taurus B2M PE=1 SV=2 | 36 | 88 | 44 | 78 | 110 | 145 | 132 | 168 | 2.30 | 0.00 |
| Q3T0X5 | Proteasome subunit alpha type-1 OS=Bos taurus PSMA1 PE=1 SV=1 | 209 | 107 | 74 | 99 | 119 | 60 | 76 | 56 | 0.64 | 0.10 |
| P81644 | Apolipoprotein A-II OS=Bos taurus APOA2 PE=1 SV=2 | 38 | 25 | 6 | 24 | 403 | 62 | 173 | 70 | 4.59 | 0.14 |
| Q05443 | Lumican OS=Bos taurus LUM PE=1 SV=1 | 66 | 58 | 295 | 32 | 198 | 47 | 57 | 46 | 0.54 | 0.76 |
| A7YWC6 | SUB1 homolog, transcriptional regulator OS=Bos taurus SUB1 PE=1 SV=1 | 88 | 94 | 235 | 175 | . | 73 | 52 | 83 | 0.51 | 0.17 |
| F1N5T0 | Protein CutA OS=Bos taurus CUTA PE=4 SV=1 | 208 | 122 | 148 | 87 | . | 59 | 68 | 109 | 0.79 | 0.33 |
| Q5E9A3 | Poly(rC)-binding protein 1 OS=Bos taurus PCBP1 PE=2 SV=1 | 181 | 71 | 146 | 98 | 33 | 92 | 91 | 89 | 0.45 | 0.28 |
| P20072 | Annexin A7 OS=Bos taurus ANXA7 PE=1 SV=2 | 253 | 56 | 122 | 98 | 50 | 96 | 90 | 37 | 0.42 | 0.30 |
| F1N726 | Glycoprotein 2 OS=Bos taurus GP2 PE=1 SV=1 | 107 | 132 | 159 | 99 | 77 | 80 | 58 | 88 | 0.58 | 0.09 |
| G3N2H4 | Azurocidin 1 OS=Bos taurus AZU1 PE=1 SV=1 | 24 | 50 | 41 | 343 | 23 | 126 | 155 | 39 | 0.38 | 0.78 |
| Q3ZCK9 | Proteasome subunit alpha type-4 OS=Bos taurus PSMA4 PE=1 SV=1 | 145 | 107 | 171 | 110 | . | 107 | 77 | 84 | 0.88 | 0.29 |
| Q2KII3 | Hepatitis A virus cellular receptor 1 N-terminal domain containing protein OS=Bos taurus MGC137099 PE=2 SV=1 | 55 | 107 | 165 | 114 | . | 117 | 139 | 104 | 1.26 | 0.51 |
| Q3T0M7 | Ran-specific GTPase-activating protein OS=Bos taurus RANBP1 PE=2 SV=1 | 111 | 52 | 143 | 91 | 68 | 163 | 94 | 78 | 0.86 | 0.97 |
| E1BL29 | Bleomycin hydrolase OS=Bos taurus BLMH PE=4 SV=1 | 191 | 73 | 99 | 91 | 89 | 82 | 98 | 77 | 0.77 | 0.37 |
| P35466 | Protein S100-A4 OS=Bos taurus S100A4 PE=1 SV=2 | 254 | 61 | 349 | 58 | 6 | 28 | 20 | 25 | 0.06 | 0.12 |
| F1MW68 | Cathepsin Z OS=Bos taurus CTSZ PE=3 SV=1 | 32 | 159 | 79 | 109 | 22 | 135 | 94 | 170 | 0.98 | 0.61 |
| P61157 | Actin-related protein 3 OS=Bos taurus ACTR3 PE=1 SV=3 | 84 | 90 | 80 | 184 | 42 | 81 | 109 | 131 | 0.76 | 0.39 |
| Q58DL9 | Phospholipid transfer protein isoform a OS=Bos taurus PLTP PE=2 SV=1 | 31 | 82 | 61 | 303 | 64 | 124 | 64 | 73 | 0.64 | 0.60 |
| O18789 | 40S ribosomal protein S2 OS=Bos taurus RPS2 PE=2 SV=2 | 102 | 110 | 188 | 133 | 70 | 110 | 59 | 28 | 0.39 | 0.11 |
| Q5EA79 | Aldose 1-epimerase OS=Bos taurus GALM PE=2 SV=1 | 105 | 123 | 177 | 97 | . | 77 | 82 | 139 | 0.90 | 0.49 |
| G8JKY5 | Thymosin beta-4 OS=Bos taurus TMSB4X PE=4 SV=1 | 189 | 62 | 159 | 84 | 55 | 71 | 74 | 105 | 0.55 | 0.29 |
| A1L528 | RAB1A, member RAS oncogene family OS=Bos taurus RAB1A PE=1 SV=1 | 153 | 90 | 132 | 150 | 51 | 87 | 79 | 59 | 0.48 | 0.07 |
| E1BGJ0 | LDL receptor related protein 1 OS=Bos taurus LRP1 PE=1 SV=2 | 39 | 200 | 63 | 124 | 85 | 88 | 46 | 156 | 0.81 | 0.74 |
| E1BCU8 | Uncharacterized protein OS=Bos taurus ICOSLG PE=4 SV=1 | 55 | 177 | 81 | 153 | 81 | 106 | 61 | 86 | 0.73 | 0.25 |
| Q3SYW3 | Hematopoietic cell-specific Lyn substrate 1 OS=Bos taurus HCLS1 PE=1 SV=1 | 37 | 74 | 57 | 254 | 64 | 106 | 118 | 90 | 0.87 | 0.84 |
| F1MJV5 | Ubiquitin carboxyl-terminal hydrolase 14 OS=Bos taurus USP14 PE=3 SV=2 | 108 | 68 | 176 | 51 | 176 | 117 | 65 | 39 | 0.77 | 0.97 |
| P54228 | Cathelicidin-6 OS=Bos taurus CATHL6 PE=1 SV=1 | 8 | 57 | 31 | 505 | . | 83 | 71 | 46 | 0.33 | 0.51 |
| A0JNP2 | Secretoglobin family 1D member OS=Bos taurus SCGB1D PE=3 SV=2 | 104 | 214 | 63 | 132 | 18 | 90 | 34 | 146 | 0.37 | 0.16 |
| A7E3S8 | Heat shock 70kD protein binding protein OS=Bos taurus ST13 PE=1 SV=1 | 185 | 86 | 132 | 76 | 79 | 82 | 76 | 85 | 0.67 | 0.23 |
| Q3ZBV8 | Threonine--tRNA ligase, cytoplasmic OS=Bos taurus TARS PE=2 SV=1 | 107 | 93 | 111 | 108 | 109 | 77 | 105 | 90 | 0.90 | 0.13 |
| F1N306 | Advanced glycosylation end product-specific receptor OS=Bos taurus AGER PE=4 SV=1 | 43 | 45 | 343 | 38 | 234 | 39 | 26 | 31 | 0.26 | 0.76 |
| E1BJ08 | Uncharacterized protein OS=Bos taurus GSTO1 PE=1 SV=2 | 115 | 93 | 219 | 93 | 24 | 82 | 90 | 84 | 0.43 | 0.14 |
| P55906 | Transforming growth factor-beta-induced protein ig-h3 OS=Bos taurus TGFBI PE=1 SV=2 | 69 | 215 | 80 | 82 | 63 | 83 | 51 | 157 | 0.70 | 0.63 |
| F1MUT3 | Xanthine dehydrogenase/oxidase OS=Bos taurus XDH PE=4 SV=1 | 73 | 83 | 126 | 140 | 55 | 105 | 76 | 143 | 0.84 | 0.53 |
| F6Q234 | Peptidase D OS=Bos taurus PEPD PE=3 SV=1 | 42 | 64 | 38 | 85 | 394 | 77 | 57 | 45 | 1.15 | 0.41 |
| Q2KIS7 | Tetranectin OS=Bos taurus CLEC3B PE=2 SV=1 | 46 | 61 | 58 | 34 | 150 | 86 | 218 | 147 | 2.64 | 0.04 |
| G3N0V2 | Keratin 1 OS=Bos taurus KRT1 PE=1 SV=1 | 57 | 53 | 73 | 292 | 87 | 155 | 38 | 46 | 0.43 | 0.65 |
| A5PJT7 | ECM1 protein OS=Bos taurus ECM1 PE=2 SV=1 | 47 | 77 | 45 | 43 | 188 | 151 | 152 | 98 | 2.68 | 0.02 |
| Q3T0W4 | Protein phosphatase 1 regulatory subunit 7 OS=Bos taurus PPP1R7 PE=1 SV=1 | 122 | 78 | 227 | 65 | 59 | 129 | 68 | 52 | 0.55 | 0.38 |
| Q3SZ19 | 26S proteasome non-ATPase regulatory subunit 9 OS=Bos taurus PSMD9 PE=1 SV=1 | 172 | 94 | 159 | 74 | 69 | 100 | 73 | 60 | 0.58 | 0.16 |
| A4FUZ1 | Lactoylglutathione lyase OS=Bos taurus GLO1 PE=1 SV=1 | 291 | 61 | 185 | 44 | 75 | 38 | 53 | 54 | 0.41 | 0.18 |
| A7Z067 | CSF1R protein OS=Bos taurus CSF1R PE=2 SV=1 | 45 | 123 | 13 | 116 | 90 | 166 | 189 | 58 | 1.21 | 0.36 |
| G3N088 | Mimecan OS=Bos taurus OGN PE=4 SV=1 | 52 | 50 | 446 | 27 | 88 | 30 | 55 | 52 | 0.37 | 0.45 |
| Q32LM2 | Small glutamine-rich tetratricopeptide repeat-containing protein alpha OS=Bos taurus SGTA PE=2 SV=1 | 163 | 97 | 138 | 104 | 34 | 122 | 82 | 60 | 0.45 | 0.20 |
| Q3MHG3 | Sulfurtransferase OS=Bos taurus MPST PE=1 SV=1 | 90 | 108 | 190 | 117 | 41 | 84 | 59 | 113 | 0.52 | 0.16 |
| F1N4K1 | Phosphoribosylformylglycinamidine synthase OS=Bos taurus PFAS PE=3 SV=2 | 130 | 75 | 65 | 95 | 81 | 92 | 146 | 116 | 1.08 | 0.56 |
| F1N6H1 | LDL receptor related protein 2 OS=Bos taurus LRP2 PE=1 SV=2 | 37 | 87 | 58 | 119 | 111 | 75 | 103 | 210 | 1.53 | 0.12 |
| F1N045 | Complement component C7 OS=Bos taurus C7 PE=4 SV=1 | 74 | 86 | 79 | 73 | 259 | 61 | 91 | 77 | 1.13 | 0.43 |
| Q76I81 | 40S ribosomal protein S12 OS=Bos taurus RPS12 PE=2 SV=1 | 134 | 56 | 124 | 106 | 16 | 74 | 136 | 155 | 0.37 | 0.81 |
| A0A140T8D4 | Legumain OS=Bos taurus LGMN PE=4 SV=1 | 46 | 147 | 54 | 98 | . | 156 | 107 | 193 | 2.05 | 0.17 |
| Q09430 | Profilin-2 OS=Bos taurus PFN2 PE=1 SV=2 | 279 | 71 | 149 | 39 | 64 | 65 | 73 | 60 | 0.49 | 0.28 |
| E1BBY7 | Heat shock protein family A (Hsp70) member 4 OS=Bos taurus HSPA4 PE=1 SV=2 | 118 | 61 | 72 | 109 | 129 | 94 | 112 | 106 | 1.23 | 0.14 |
| Q2TBW6 | ADP-ribosylation factor-like protein 3 OS=Bos taurus ARL3 PE=2 SV=1 | 246 | 68 | 99 | 66 | 60 | 97 | 108 | 55 | 0.58 | 0.48 |
| F1MXX6 | Lactadherin OS=Bos taurus MFGE8 PE=4 SV=1 | 63 | 381 | 92 | 46 | 18 | 89 | 13 | 99 | 0.26 | 0.30 |
| Q2KIX7 | Protein HP-25 homolog 1 OS=Bos taurus OX=9913 PE=1 SV=1 | 29 | 84 | 29 | 56 | 208 | 113 | 170 | 111 | 2.55 | 0.07 |
| F1MWI1 | Clusterin OS=Bos taurus OX=9913 PE=3 SV=2 | 35 | 100 | 39 | 151 | 55 | 90 | 89 | 242 | 1.42 | 0.18 |
| Q95M18 | Endoplasmin OS=Bos taurus HSP90B1 PE=2 SV=1 | 72 | 132 | 93 | 125 | 91 | 102 | 125 | 60 | 0.81 | 0.65 |
| Q0IID9 | Glycerol kinase OS=Bos taurus GK PE=2 SV=1 | 133 | 70 | 84 | 56 | 137 | 125 | 100 | 96 | 1.36 | 0.09 |
| G3MXG6 | Uncharacterized protein OS=Bos taurus OX=9913 PE=4 SV=1 | 15 | 172 | 25 | 88 | 40 | 221 | 123 | 117 | 1.91 | 0.06 |
| E1BMK2 | Uncharacterized protein OS=Bos taurus OX=9913 PE=4 SV=2 | 41 | 101 | 54 | 55 | 283 | 103 | 86 | 77 | 1.62 | 0.28 |
| P28782 | Protein S100-A8 OS=Bos taurus S100A8 PE=1 SV=2 | 37 | 65 | 49 | 366 | 22 | 80 | 78 | 102 | 0.60 | 0.46 |
| F1N4W5 | Membrane cofactor protein OS=Bos taurus CD46 PE=4 SV=2 | 75 | 170 | 110 | 85 | . | 44 | 23 | 294 | 0.45 | 0.99 |
| A0A140T843 | Beta-2-glycoprotein 1 OS=Bos taurus APOH PE=4 SV=1 | 59 | 240 | 115 | 97 | . | 138 | 92 | 59 | 0.87 | 0.15 |
| Q32PA1 | CD59 molecule, complement regulatory protein OS=Bos taurus CD59 PE=2 SV=1 | 44 | 248 | 105 | 161 | . | 73 | 34 | 135 | 0.52 | 0.18 |
| A6QQ07 | Biotinidase OS=Bos taurus BTD PE=2 SV=1 | 83 | 73 | 119 | 208 | 42 | 189 | 42 | 44 | 0.40 | 0.53 |
| Q9XT56 | Junctional adhesion molecule A OS=Bos taurus F11R PE=2 SV=1 | 64 | 238 | 78 | 147 | . | 55 | 26 | 192 | 0.49 | 0.44 |
| G3MX66 | Vitelline membrane outer layer 1 homolog OS=Bos taurus VMO1 PE=4 SV=1 | 21 | 68 | 67 | 359 | 55 | 59 | 38 | 135 | 0.67 | 0.39 |
| Q2KJE7 | Proteasome (Prosome, macropain) activator subunit 1 (PA28 alpha) OS=Bos taurus PSME1 PE=2 SV=1 | 178 | 91 | 73 | 82 | 62 | 136 | 118 | 62 | 0.73 | 0.78 |
| Q3T0M0 | Vacuolar protein sorting-associated protein 29 OS=Bos taurus VPS29 PE=2 SV=1 | 133 | 119 | 188 | 116 | 30 | 82 | 64 | 68 | 0.38 | 0.03 |
| Q56JZ9 | Glia maturation factor gamma OS=Bos taurus GMFG PE=2 SV=1 | 133 | 77 | 134 | 183 | . | 90 | 94 | 91 | 0.93 | 0.32 |
| F1MNN7 | Lipopolysaccharide-binding protein OS=Bos taurus LBP PE=4 SV=1 | 11 | 13 | . | 51 | 510 | 77 | 106 | 33 | 2.29 | 0.38 |
| F1MH27 | Acidic mammalian chitinase OS=Bos taurus CHIA PE=3 SV=1 | 45 | 124 | 76 | 74 | 192 | 111 | 100 | 79 | 1.32 | 0.34 |
| Q0P5E0 | Lymphocyte-specific protein 1 OS=Bos taurus LSP1 PE=1 SV=1 | 38 | 46 | 66 | 260 | 54 | 88 | 122 | 126 | 1.05 | 0.92 |
| Q29RK4 | UV excision repair protein RAD23 homolog B OS=Bos taurus RAD23B PE=2 SV=1 | 126 | 90 | 89 | 81 | 75 | 88 | 113 | 139 | 0.98 | 0.77 |
| P37141 | Glutathione peroxidase 3 OS=Bos taurus GPX3 PE=2 SV=2 | 36 | 51 | 44 | 78 | 206 | 107 | 186 | 92 | 2.31 | 0.08 |
| Q3SYU6 | Calponin-2 OS=Bos taurus CNN2 PE=2 SV=3 | 163 | 48 | 114 | 108 | 81 | 97 | 105 | 84 | 0.82 | 0.58 |
| Q0VFX8 | Cysteine-rich protein 2 OS=Bos taurus CRIP2 PE=2 SV=1 | 384 | . | 220 | 70 | . | 69 | 27 | 30 | . | 0.37 |
| P40673 | High mobility group protein B2 OS=Bos taurus HMGB2 PE=1 SV=3 | 11 | 43 | 103 | 444 | 49 | 106 | 27 | 17 | 0.13 | 0.44 |
| A7MBJ4 | Receptor-type tyrosine-protein phosphatase F OS=Bos taurus PTPRF PE=2 SV=1 | 49 | 95 | 50 | 84 | 126 | 202 | 112 | 82 | 1.72 | 0.08 |
| Q3T0D0 | Heterogeneous nuclear ribonucleoprotein K OS=Bos taurus HNRNPK PE=2 SV=1 | 99 | 115 | 123 | 254 | . | 41 | 79 | 89 | 0.49 | 0.12 |
| Q3ZBD7 | Glucose-6-phosphate isomerase OS=Bos taurus GPI PE=2 SV=4 | 61 | 87 | 100 | 140 | 33 | 107 | 138 | 134 | 0.90 | 0.70 |
| P19660 | Cathelicidin-2 OS=Bos taurus CATHL2 PE=1 SV=2 | 6 | 33 | 31 | 497 | . | 93 | 88 | 52 | 0.35 | 0.58 |
| G3N303 | Small ubiquitin-related modifier 2 OS=Bos taurus SUMO2 PE=4 SV=1 | 224 | 71 | 172 | 92 | 36 | 46 | 66 | 93 | 0.35 | 0.16 |
| P54229 | Cathelicidin-5 OS=Bos taurus CATHL5 PE=1 SV=1 | 6 | 85 | 20 | 472 | 15 | 167 | 26 | 11 | 0.09 | 0.52 |
| Q8MJ50 | Osteoclast-stimulating factor 1 OS=Bos taurus OSTF1 PE=2 SV=1 | 185 | 79 | 206 | 83 | . | 105 | 67 | 76 | 0.82 | 0.51 |
| Q3ZBH2 | NAD(P)H dehydrogenase, quinone 1 OS=Bos taurus NQO1 PE=2 SV=1 | 259 | 100 | 185 | 53 | 26 | 84 | 56 | 37 | 0.25 | 0.15 |
| Q5E951 | Tubulin-folding cofactor B OS=Bos taurus TBCB PE=2 SV=1 | 209 | 68 | 180 | 70 | 35 | 70 | 75 | 93 | 0.40 | 0.26 |
| G5E589 | Proteasome subunit beta type OS=Bos taurus PSMB1 PE=3 SV=1 | 97 | 126 | 151 | 101 | 125 | 96 | 57 | 46 | 0.58 | 0.23 |
| Q71SP7 | Fatty acid synthase OS=Bos taurus FASN PE=2 SV=1 | 68 | 64 | 66 | 192 | 30 | 91 | 120 | 169 | 0.85 | 0.83 |
| G3N2H5 | Protein S100 OS=Bos taurus S100A12 PE=1 SV=1 | 26 | 30 | 50 | 540 | 8 | 35 | 51 | 60 | 0.28 | 0.38 |
| P82943 | Regakine-1 OS=Bos taurus OX=9913 PE=1 SV=2 | 75 | 48 | 60 | 46 | 125 | 177 | 185 | 84 | 2.30 | 0.04 |
| P17248 | Tryptophan--tRNA ligase, cytoplasmic OS=Bos taurus WARS PE=1 SV=3 | 78 | 99 | 118 | 68 | 58 | 134 | 128 | 117 | 1.11 | 0.32 |
| Q58CQ2 | Actin-related protein 2/3 complex subunit 1B OS=Bos taurus ARPC1B PE=1 SV=4 | 74 | 82 | 80 | 204 | 51 | 97 | 108 | 106 | 0.81 | 0.54 |
| E1BCW3 | ATP-dependent 6-phosphofructokinase OS=Bos taurus PFKP PE=3 SV=2 | 28 | 114 | 45 | 168 | 316 | 69 | 28 | 33 | 0.47 | 0.82 |
| Q0II59 | Pyridoxal kinase OS=Bos taurus PDXK PE=2 SV=1 | 144 | 82 | 179 | 54 | 153 | 89 | 72 | 28 | 0.64 | 0.35 |
| F1N2W0 | Prostaglandin reductase 1 OS=Bos taurus PTGR1 PE=4 SV=1 | 259 | 40 | 303 | 39 | 41 | 34 | 39 | 45 | 0.25 | 0.19 |
| Q3SZA6 | Syndecan binding protein (Syntenin) OS=Bos taurus SDCBP PE=2 SV=1 | 36 | 131 | 104 | 256 | . | 74 | 50 | 150 | 0.72 | 0.05 |
| G3MY87 | Maltase-glucoamylase OS=Bos taurus MGAM PE=4 SV=1 | 32 | 43 | 50 | 76 | 62 | 353 | 152 | 32 | 1.19 | 0.28 |
| A7YY64 | Hexosyltransferase OS=Bos taurus B3GNT2 PE=2 SV=1 | 63 | 193 | 56 | 144 | 43 | 71 | 51 | 179 | 0.66 | 0.47 |
| Q3SX44 | N(G),N(G)-dimethylarginine dimethylaminohydrolase 2 OS=Bos taurus DDAH2 PE=2 SV=1 | 127 | 76 | 167 | 52 | 111 | 119 | 104 | 44 | 0.87 | 0.64 |
| Q865V6 | Macrophage-capping protein OS=Bos taurus CAPG PE=2 SV=1 | 66 | 57 | 70 | 245 | . | 110 | 121 | 131 | 0.80 | 0.95 |
| F1MIQ2 | Very low density lipoprotein receptor OS=Bos taurus VLDLR PE=4 SV=2 | 76 | 174 | 105 | 86 | . | 96 | 68 | 196 | 0.69 | 0.98 |
| F1N431 | Farnesyl pyrophosphate synthase OS=Bos taurus FDPS PE=3 SV=1 | 25 | 72 | 315 | 57 | 70 | 136 | 52 | 73 | 0.52 | 0.69 |
| P42899 | 60S acidic ribosomal protein P2 OS=Bos taurus RPLP2 PE=3 SV=1 | 93 | 105 | 107 | 140 | 65 | 74 | 117 | 99 | 0.77 | 0.13 |
| Q3T035 | Actin-related protein 2/3 complex subunit 3 OS=Bos taurus ARPC3 PE=1 SV=3 | 98 | 42 | 54 | 174 | 54 | 112 | 175 | 91 | 0.91 | 0.76 |
| P39873 | Brain ribonuclease OS=Bos taurus BRN PE=1 SV=3 | 27 | 257 | 91 | 80 | . | 90 | 38 | 218 | 0.53 | 0.79 |
| F1MQF6 | Apoptosis-associated speck-like protein-containing a CARD OS=Bos taurus PYCARD PE=4 SV=1 | 38 | 101 | 41 | 185 | . | 116 | 139 | 181 | 0.96 | 0.37 |
| P26201 | Platelet glycoprotein 4 OS=Bos taurus CD36 PE=1 SV=5 | 29 | 79 | 158 | 143 | 76 | 49 | 61 | 205 | 0.75 | 0.90 |
| Q3ZBL1 | 1,2-dihydroxy-3-keto-5-methylthiopentene dioxygenase OS=Bos taurus ADI1 PE=2 SV=2 | 240 | 71 | 111 | 68 | . | 95 | 141 | 74 | 0.90 | 0.11 |
| Q5E9G3 | Proteasome activator complex subunit 2 OS=Bos taurus PSME2 PE=1 SV=3 | 35 | 181 | 49 | 86 | 263 | 76 | 55 | 55 | 0.81 | 0.75 |
| Q9GLX9 | Spondin-1 OS=Bos taurus SPON1 PE=1 SV=1 | 64 | 96 | 269 | 47 | 47 | 76 | 91 | 109 | 0.66 | 0.51 |
| E1BKM4 | Programmed cell death 6 interacting protein OS=Bos taurus PDCD6IP PE=1 SV=2 | 63 | 102 | 66 | 82 | 165 | 127 | 82 | 112 | 1.47 | 0.12 |
| Q3MHR0 | Acyl-protein thioesterase 1 OS=Bos taurus LYPLA1 PE=2 SV=1 | 124 | 80 | 196 | 83 | 90 | 96 | 72 | 58 | 0.63 | 0.26 |
| G3X847 | Uncharacterized protein OS=Bos taurus PI3 PE=4 SV=1 | 12 | 136 | 40 | 316 | 45 | 96 | 66 | 88 | 0.68 | 0.45 |
| Q28085 | Complement factor H OS=Bos taurus CFH PE=1 SV=3 | 60 | 87 | . | 36 | 190 | 157 | 183 | 88 | 3.14 | 0.07 |
| Q2HJ57 | Coactosin-like protein OS=Bos taurus COTL1 PE=2 SV=3 | 55 | 152 | 61 | 210 | 23 | 52 | 83 | 164 | 0.54 | 0.21 |
| A6QPT4 | MPO protein OS=Bos taurus MPO PE=1 SV=1 | 24 | 94 | 191 | 282 | 19 | 64 | 71 | 57 | 0.38 | 0.15 |
| Q32PA4 | 14 kDa phosphohistidine phosphatase OS=Bos taurus PHPT1 PE=2 SV=1 | 178 | 70 | 217 | 48 | 51 | 69 | 82 | 85 | 0.52 | 0.29 |
| E1BFV0 | Karyopherin subunit beta 1 OS=Bos taurus KPNB1 PE=1 SV=2 | 168 | 86 | 95 | 89 | . | 94 | 158 | 110 | 1.72 | 0.21 |
| F1MUB9 | Serine protease 8 OS=Bos taurus PRSS8 PE=3 SV=1 | 29 | 151 | 165 | 194 | . | 123 | 24 | 113 | 0.41 | 0.12 |
| G3X701 | Uncharacterized protein OS=Bos taurus LOC100300091 PE=3 SV=1 | 63 | 82 | 84 | 37 | 377 | 34 | 50 | 73 | 0.83 | 0.49 |
| Q32PA8 | Mth938 domain-containing protein OS=Bos taurus AAMDC PE=2 SV=1 | 121 | 76 | 210 | 191 | 29 | 75 | 37 | 62 | 0.29 | 0.07 |
| Q3SZ52 | Ubiquitin-conjugating enzyme E2 variant 1 OS=Bos taurus UBE2V1 PE=2 SV=1 | 248 | 74 | 127 | 64 | 37 | 72 | 91 | 89 | 0.41 | 0.37 |
| P01035 | Cystatin-C OS=Bos taurus CST3 PE=1 SV=2 | 45 | 118 | 85 | 126 | 27 | 117 | 139 | 142 | 0.95 | 0.47 |
| G3MZZ6 | Protein-L-isoaspartate O-methyltransferase OS=Bos taurus PCMT1 PE=1 SV=1 | 202 | 67 | 102 | 123 | 50 | 85 | 107 | 65 | 0.52 | 0.31 |
| F1MME1 | Uncharacterized protein OS=Bos taurus PTPRK PE=4 SV=2 | 50 | 166 | 101 | 139 | 94 | 80 | 31 | 139 | 0.58 | 0.43 |
| Q2TBN3 | Centrin-2 OS=Bos taurus CETN2 PE=2 SV=1 | 67 | 90 | 106 | 59 | 90 | 94 | 108 | 187 | 1.33 | 0.28 |
| O46375 | Transthyretin OS=Bos taurus TTR PE=1 SV=1 | 53 | 100 | 98 | 91 | 98 | 144 | 78 | 139 | 1.28 | 0.17 |
| Q3T0S6 | 60S ribosomal protein L8 OS=Bos taurus RPL8 PE=2 SV=3 | 132 | 120 | 144 | 117 | 96 | 81 | 85 | 26 | 0.44 | 0.02 |
| G3N3L9 | Uncharacterized protein OS=Bos taurus LOC613363 PE=3 SV=1 | 12 | 25 | 10 | 255 | 154 | 141 | 169 | 33 | 0.50 | 0.63 |
| P56652 | Inter-alpha-trypsin inhibitor heavy chain H3 OS=Bos taurus ITIH3 PE=1 SV=2 | 31 | 50 | 22 | 19 | 414 | 81 | 93 | 90 | 3.51 | 0.19 |
| Q1JPE3 | Carbohydrate kinase-like OS=Bos taurus CARKL PE=1 SV=1 | 103 | 99 | 237 | 80 | 13 | 116 | 69 | 84 | 0.30 | 0.26 |
| G3X702 | Caspase-6 OS=Bos taurus CASP6 PE=3 SV=1 | 93 | 125 | 169 | 100 | 89 | 67 | 59 | 98 | 0.59 | 0.19 |
| P61223 | Ras-related protein Rap-1b OS=Bos taurus RAP1B PE=2 SV=1 | 84 | 115 | 110 | 213 | 70 | 74 | 63 | 71 | 0.53 | 0.11 |
| A6QR61 | SNX12 protein OS=Bos taurus SNX12 PE=2 SV=1 | 117 | 61 | 92 | 76 | 70 | 127 | 208 | 50 | 0.97 | 0.53 |
| P52175 | Nucleoside diphosphate kinase A 2 OS=Bos taurus NME1-2 PE=1 SV=3 | 74 | 53 | 155 | 234 | . | 136 | 82 | 67 | 0.69 | 0.55 |
| P67774 | Serine/threonine-protein phosphatase 2A catalytic subunit alpha isoform OS=Bos taurus PPP2CA PE=1 SV=1 | 189 | 83 | 105 | 90 | 68 | 79 | 104 | 83 | 0.68 | 0.34 |
| A1A4P5 | Prefoldin subunit 2 OS=Bos taurus PFDN2 PE=2 SV=1 | 74 | 37 | 55 | 73 | 426 | 57 | 30 | 48 | 0.96 | 0.44 |
| Q0IIA3 | Sorcin OS=Bos taurus SRI PE=2 SV=1 | 82 | 109 | 75 | 116 | 131 | 111 | 101 | 76 | 1.03 | 0.66 |
| P00570 | Adenylate kinase isoenzyme 1 OS=Bos taurus AK1 PE=1 SV=2 | 82 | 85 | 167 | 143 | 37 | 114 | 68 | 106 | 0.59 | 0.25 |
| Q3MHR7 | Actin-related protein 2/3 complex subunit 2 OS=Bos taurus ARPC2 PE=1 SV=1 | 122 | 74 | 83 | 236 | . | 98 | 118 | 69 | 0.82 | 0.64 |
| Q3T0T9 | 20-beta-hydroxysteroid dehydrogenase-like OS=Bos taurus MGC127133 PE=2 SV=1 | 81 | 72 | 54 | 431 | . | 37 | 101 | 26 | 0.21 | 0.45 |
| G3MZZ0 | Glutathione peroxidase OS=Bos taurus GPX2 PE=3 SV=1 | 70 | 157 | 71 | 104 | 121 | 108 | 80 | 90 | 0.98 | 0.96 |
| Q3ZBA8 | Protein NDRG2 OS=Bos taurus NDRG2 PE=2 SV=1 | 192 | 66 | 280 | 44 | 53 | 83 | 33 | 49 | 0.29 | 0.25 |
| F1MUZ9 | 60 kDa heat shock protein, mitochondrial OS=Bos taurus HSPD1 PE=1 SV=1 | 54 | 118 | 184 | 134 | 70 | 89 | 80 | 73 | 0.64 | 0.18 |
| A5PJY9 | CPNE3 protein OS=Bos taurus CPNE3 PE=1 SV=1 | 72 | 93 | 209 | 112 | 112 | 90 | 63 | 49 | 0.55 | 0.36 |
| Q3T112 | Proteasome subunit beta type-8 OS=Bos taurus PSMB8 PE=1 SV=2 | 74 | 118 | 47 | 174 | 104 | 129 | 91 | 63 | 0.82 | 0.87 |
| G5E6G2 | Uncharacterized protein OS=Bos taurus OX=9913 PE=4 SV=1 | 247 | 56 | 250 | 76 | 32 | 62 | 42 | 35 | 0.24 | 0.14 |
| Q5EA61 | Creatine kinase B-type OS=Bos taurus CKB PE=1 SV=1 | 167 | 91 | 114 | 133 | 38 | 54 | 60 | 143 | 0.45 | 0.17 |
| G3X6S5 | SH3 domain-binding glutamic acid-rich-like protein 3 OS=Bos taurus SH3BGRL3 PE=4 SV=1 | 128 | 90 | 119 | 151 | 64 | 100 | 76 | 73 | 0.61 | 0.10 |
| F1MNI4 | RAB5B, member RAS oncogene family OS=Bos taurus RAB5B PE=1 SV=1 | 195 | 75 | 141 | 114 | 40 | 83 | 83 | 69 | 0.44 | 0.16 |
| G3X6Q8 | Pentraxin-related protein PTX3 OS=Bos taurus PTX3 PE=4 SV=1 | 31 | 64 | 61 | 91 | 191 | 70 | 80 | 211 | 1.76 | 0.14 |
| G3MXP6 | Histone H2B OS=Bos taurus OX=9913 PE=3 SV=1 | 15 | 55 | 41 | 366 | 106 | 113 | 84 | 22 | 0.22 | 0.73 |
| F1MJS9 | Protein tyrosine phosphatase, receptor type C OS=Bos taurus PTPRC PE=1 SV=2 | 178 | 53 | 232 | 126 | . | 70 | 77 | 64 | 0.70 | 0.31 |
| E1B8K6 | Uncharacterized protein OS=Bos taurus NCL PE=1 SV=1 | 17 | 245 | 180 | 134 | 22 | 142 | 14 | 46 | 0.22 | 0.09 |
| A7MB62 | Actin-related protein 2 OS=Bos taurus ACTR2 PE=1 SV=1 | 80 | 118 | 106 | 182 | 47 | 107 | 101 | 60 | 0.58 | 0.21 |
| F1MQF0 | Intercellular adhesion molecule 3 OS=Bos taurus ICAM3 PE=4 SV=1 | 36 | 62 | 34 | 117 | 218 | 189 | 49 | 95 | 1.66 | 0.21 |
| P26882 | Peptidyl-prolyl cis-trans isomerase D OS=Bos taurus PPID PE=1 SV=6 | 124 | 79 | 106 | 78 | 29 | 106 | 108 | 172 | 0.62 | 0.87 |
| G3MXC8 | Actin-related protein 2/3 complex subunit 5 OS=Bos taurus ARPC5 PE=3 SV=1 | 87 | 68 | 93 | 179 | 62 | 139 | 88 | 85 | 0.79 | 0.73 |
| P37980 | Inorganic pyrophosphatase OS=Bos taurus PPA1 PE=1 SV=2 | 228 | 77 | 99 | 93 | 42 | 112 | 107 | 42 | 0.43 | 0.40 |
| Q6URK6 | Cadherin-5 OS=Bos taurus CDH5 PE=2 SV=1 | 35 | 135 | 90 | 122 | 153 | 120 | 83 | 63 | 0.91 | 0.82 |
| Q2KIH2 | ApoN protein OS=Bos taurus ApoN PE=2 SV=1 | 40 | 56 | 16 | 37 | 23 | 245 | 279 | 104 | 1.70 | 0.14 |
| F1MKB7 | Glutathione-S-transferase omega 1 OS=Bos taurus GSTO1 PE=4 SV=2 | . | 72 | 122 | 134 | . | 94 | 259 | 120 | 1.70 | 0.40 |
| Q5EAC6 | Hsp90 co-chaperone Cdc37 OS=Bos taurus CDC37 PE=2 SV=1 | 223 | 106 | 99 | 62 | 91 | 54 | 82 | 84 | 0.63 | 0.26 |
| E1BKZ1 | Glutathione-disulfide reductase OS=Bos taurus GSR PE=4 SV=2 | 95 | 145 | 172 | 124 | 24 | 105 | 26 | 109 | 0.31 | 0.10 |
| Q1JPA0 | Aldehyde dehydrogenase family 3 member B1 OS=Bos taurus ALDH3B1 PE=2 SV=1 | 44 | 120 | 115 | 125 | 182 | 106 | 39 | 70 | 0.65 | 0.97 |
| P49951 | Clathrin heavy chain 1 OS=Bos taurus CLTC PE=1 SV=1 | 46 | 84 | 365 | 158 | 31 | 84 | 21 | 12 | 0.12 | 0.21 |
| F1N226 | Amyloid beta precursor like protein 2 OS=Bos taurus APLP2 PE=4 SV=2 | 49 | 258 | 59 | 106 | 41 | 95 | 53 | 140 | 0.69 | 0.47 |
| F1MCT8 | Synaptotagmin binding cytoplasmic RNA interacting protein OS=Bos taurus SYNCRIP PE=1 SV=2 | 64 | 140 | 125 | 110 | 83 | 47 | 83 | 149 | 0.67 | 0.57 |
| P28291 | C-C motif chemokine 2 OS=Bos taurus CCL2 PE=3 SV=1 | . | 109 | 61 | 84 | 155 | 147 | 202 | 42 | 1.32 | 0.48 |
| Q1RMH8 | Sorting nexin-3 OS=Bos taurus SNX3 PE=2 SV=3 | 176 | 51 | 205 | 74 | 78 | 65 | 69 | 84 | 0.58 | 0.26 |
| A6QQ11 | PGM2 protein (Fragment) OS=Bos taurus PGM2 PE=1 SV=1 | 148 | 103 | 162 | 90 | 31 | 83 | 95 | 87 | 0.46 | 0.14 |
| A7MBJ8 | LAMP3 protein OS=Bos taurus LAMP3 PE=2 SV=1 | 44 | 213 | 112 | 154 |  | 41 | 22 | 215 | 0.36 | 0.43 |
| Q3ZC84 | Cytosolic non-specific dipeptidase OS=Bos taurus CNDP2 PE=2 SV=1 | 125 | 78 | 121 | 64 | 62 | 57 | 91 | 203 | 0.80 | 0.89 |
| F1MWE0 | Proteasome 26S subunit, ATPase 3 OS=Bos taurus PSMC3 PE=1 SV=2 | 107 | 110 | 158 | 87 | 140 | 91 | 58 | 51 | 0.62 | 0.35 |
| Q27971 | Calpain-2 catalytic subunit OS=Bos taurus CAPN2 PE=2 SV=2 | 137 | 118 | 233 | 80 | 41 | 81 | 55 | 55 | 0.38 | 0.10 |
| Q148J6 | Actin-related protein 2/3 complex subunit 4 OS=Bos taurus ARPC4 PE=1 SV=3 | 50 | 111 | 80 | 159 | 129 | 116 | 80 | 76 | 0.90 | 0.99 |
| Q05927 | 5'-nucleotidase OS=Bos taurus NT5E PE=1 SV=2 | 23 | 71 | 60 | 312 | 77 | 221 | 18 | 18 | 0.19 | 0.75 |
| Q3SZK8 | Na(+)/H(+) exchange regulatory cofactor NHE-RF1 OS=Bos taurus SLC9A3R1 PE=2 SV=1 | 39 | 70 | 264 | 104 | 15 | 72 | 40 | 195 | 0.38 | 0.60 |
| Q0V8R6 | Beta-hexosaminidase subunit alpha OS=Bos taurus HEXA PE=2 SV=1 | 40 | 102 | 74 | 84 | 73 | 142 | 143 | 143 | 1.69 | 0.01 |
| P30932 | CD9 antigen OS=Bos taurus CD9 PE=2 SV=2 | 37 | 92 | 72 | 178 | 64 | 99 | 104 | 153 | 1.19 | 0.48 |
| O02659 | Mannose-binding protein C OS=Bos taurus MBL PE=2 SV=1 | 50 | 128 | 42 | 68 | 230 | 84 | 116 | 84 | 1.38 | 0.32 |
| P02633 | Protein S100-G OS=Bos taurus S100G PE=1 SV=3 | 116 | 118 | 70 | 112 | 45 | 66 | 126 | 147 | 0.71 | 0.82 |
| Q2HJH1 | Aspartyl aminopeptidase OS=Bos taurus DNPEP PE=1 SV=1 | 149 | 121 | 148 | 102 | 47 | 102 | 65 | 66 | 0.49 | 0.05 |
| F1MWK8 | Protein tyrosine kinase 7 (inactive) OS=Bos taurus PTK7 PE=3 SV=2 | 34 | 233 | 88 | 108 | . | 73 | 29 | 236 | 0.60 | 0.75 |
| F1MLI8 | Uncharacterized protein OS=Bos taurus PODXL PE=4 SV=2 | 41 | 171 | 116 | 94 | . | 98 | 34 | 248 | 0.72 | 0.99 |
| A7MBJ5 | Cullin-associated NEDD8-dissociated protein 1 OS=Bos taurus CAND1 PE=2 SV=1 | 188 | 137 | 112 | 88 | 62 | 108 | 79 | 26 | 0.44 | 0.07 |
| P00432 | Catalase OS=Bos taurus CAT PE=1 SV=3 | 56 | 91 | 107 | 97 | 103 | 103 | 145 | 98 | 1.26 | 0.11 |
| P31404 | V-type proton ATPase catalytic subunit A OS=Bos taurus ATP6V1A PE=2 SV=2 | 100 | 120 | 133 | 246 | . | 68 | 45 | 87 | 0.53 | 0.09 |
| G3X8E3 | Beta-microseminoprotein OS=Bos taurus MSMB PE=3 SV=1 | 15 | 62 | 97 | 399 | . | 22 | 20 | 185 | 0.41 | 0.17 |
| F1MRR1 | Kringle containing transmembrane protein 1 OS=Bos taurus KREMEN1 PE=4 SV=1 | 42 | 181 | 59 | 132 | 67 | 65 | 38 | 217 | 0.71 | 0.88 |
| F1MNN6 | Major vault protein OS=Bos taurus MVP PE=4 SV=1 | 95 | 110 | 39 | 97 | 127 | 80 | 151 | 102 | 1.20 | 0.40 |
| E1BLR9 | Carboxypeptidase D OS=Bos taurus CPD PE=4 SV=2 | 32 | 199 | . | 203 | . | 123 | 80 | 164 | . | 0.20 |
| E1B9H5 | Transforming growth factor beta receptor 3 OS=Bos taurus TGFBR3 PE=4 SV=2 | 36 | 217 | 101 | 142 | 67 | 105 | 35 | 97 | 0.58 | 0.21 |
| Q07130 | UTP--glucose-1-phosphate uridylyltransferase OS=Bos taurus UGP2 PE=1 SV=2 | 99 | 94 | 124 | 164 | . | 143 | 90 | 87 | 1.02 | 0.63 |
| F1MD44 | Uncharacterized protein OS=Bos taurus LOC537017 PE=4 SV=2 | 86 | 234 | 83 | 122 | . | 91 | 87 | 97 | 0.84 | 0.35 |
| Q5E9K0 | Proteasome subunit beta type-2 OS=Bos taurus PSMB2 PE=1 SV=1 | 96 | 140 | 103 | 242 | 18 | 87 | 60 | 54 | 0.31 | 0.07 |
| P19427 | Dermatopontin OS=Bos taurus DPT PE=1 SV=3 | 77 | 100 | 316 | 55 | . | 78 | 104 | 70 | 0.78 | 0.41 |
| A5PK51 | Nicotinate phosphoribosyltransferase OS=Bos taurus NAPRT PE=2 SV=2 | 186 | 44 | 199 | 91 | . | 78 | 92 | 110 | 1.13 | 0.73 |
| Q3SZA5 | Spermine synthase OS=Bos taurus SMS PE=2 SV=1 | 250 | 74 | 119 | 78 | 69 | 102 | 62 | 46 | 0.50 | 0.26 |
| Q3ZC42 | Alcohol dehydrogenase class-3 OS=Bos taurus ADH5 PE=2 SV=1 | 284 | 75 | 246 | 35 | 43 | 48 | 45 | 23 | 0.26 | 0.13 |
| F1MHF7 | Thioredoxin domain containing 17 OS=Bos taurus TXNDC17 PE=1 SV=1 | 189 | 69 | 137 | 46 | 72 | 58 | 111 | 118 | 0.74 | 0.63 |
| Q2TA49 | Vasodilator-stimulated phosphoprotein OS=Bos taurus VASP PE=2 SV=3 | 40 | 53 | 68 | 212 | 149 | 106 | 103 | 70 | 0.90 | 0.82 |
| E1BJH7 | Ribokinase OS=Bos taurus RBKS PE=3 SV=2 | 102 | 235 | 44 | 114 | 55 | 102 | 90 | 59 | 0.61 | 0.29 |
| E1BNR9 | Semaphorin-7A precursor OS=Bos taurus SEMA7A PE=3 SV=1 | 174 | 147 | 166 | 102 | . | 53 | 57 | 101 | 0.60 | 0.18 |
| Q3MHP1 | Ubiquitin-conjugating enzyme E2 L3 OS=Bos taurus UBE2L3 PE=2 SV=1 | 137 | 66 | 204 | 80 | 74 | 93 | 68 | 79 | 0.61 | 0.32 |
| Q3ZCF3 | S-phase kinase-associated protein 1 OS=Bos taurus SKP1 PE=2 SV=1 | 164 | 89 | 242 | 73 | 25 | 89 | 56 | 62 | 0.31 | 0.17 |
| A6H767 | Nucleosome assembly protein 1-like 1 OS=Bos taurus NAP1L1 PE=2 SV=1 | 136 | 66 | 71 | 86 | 122 | 119 | 126 | 75 | 1.18 | 0.36 |
| E1BJ78 | Sulfotransferase OS=Bos taurus SULT1C4 PE=3 SV=2 | 222 | 80 | 134 | 62 | 77 | 89 | 95 | 41 | 0.59 | 0.24 |
| P08904 | Ribonuclease K6 OS=Bos taurus RNASE6 PE=1 SV=3 | 59 | 142 | 87 | 101 | . | 83 | 66 | 262 | 1.18 | 0.73 |
| F1MMW8 | Serum amyloid A protein OS=Bos taurus M-SAA3.2 PE=3 SV=1 | . | . | 59 | 649 | . | 29 | 42 | 21 | . | 0.48 |
| F1MD66 | Annexin OS=Bos taurus ANXA11 PE=3 SV=2 | 126 | 100 | 92 | 140 | 89 | 96 | 89 | 69 | 0.73 | 0.18 |
| A6QR56 | Aldehyde dehydrogenase family 16 member A1 OS=Bos taurus ALDH16A1 PE=2 SV=1 | 137 | 104 | 101 | 80 | 124 | 69 | 95 | 91 | 0.88 | 0.35 |
| Q5E973 | 60S ribosomal protein L18 OS=Bos taurus RPL18 PE=2 SV=3 | 71 | 175 | 262 | 111 | . | 113 | 38 | 31 | 0.33 | 0.14 |
| G3MXJ5 | Lysosomal associated membrane protein 2 OS=Bos taurus LAMP2 PE=1 SV=1 | 73 | 197 | 90 | 134 | . | 60 | 44 | 201 | 0.67 | 0.58 |
| A6H768 | Galactokinase OS=Bos taurus GALK1 PE=2 SV=2 | 56 | 76 | 128 | 233 | . | 76 | 82 | 150 | 0.97 | 0.22 |
| A3KMX8 | Protein-glutamate O-methyltransferase OS=Bos taurus ARMT1 PE=2 SV=1 | 21 | 100 | 111 | 96 | 162 | 228 | 49 | 32 | 0.68 | 0.58 |
| F6PW02 | Protein TFG OS=Bos taurus TFG PE=1 SV=1 | 76 | 47 | 67 | 77 | 310 | 85 | 69 | 69 | 1.39 | 0.32 |
| Q1JPJ2 | Xaa-Pro aminopeptidase 1 OS=Bos taurus XPNPEP1 PE=2 SV=1 | 258 | 72 | 106 | 70 | . | 67 | 135 | 92 | 1.52 | 0.28 |
| Q3MHK9 | Fascin OS=Bos taurus FSCN1 PE=1 SV=1 | 326 | 144 | 82 | 56 | . | 45 | 46 | 100 | 0.73 | 0.54 |
| F1MV32 | Adhesion G protein-coupled receptor F5 OS=Bos taurus ADGRF5 PE=3 SV=2 | 55 | 215 | 68 | 148 | 52 | 103 | 36 | 123 | 0.65 | 0.17 |
| Q1RMR9 | Protein kinase C and casein kinase substrate in neurons 2 OS=Bos taurus PACSIN2 PE=2 SV=1 | 188 | 102 | 107 | 69 | 170 | 83 | 56 | 26 | 0.58 | 0.03 |
| Q32L46 | 5'-nucleotidase OS=Bos taurus NT5C3A PE=2 SV=1 | 162 | 148 | 147 | 73 | . | 66 | 83 | 121 | 0.87 | 0.51 |
| Q3ZC22 | Heat shock factor-binding protein 1 OS=Bos taurus HSBP1 PE=3 SV=1 | 150 | 71 | 95 | 51 | 166 | 65 | 108 | 95 | 1.17 | 0.21 |
| F6QLM5 | Glycogenin 1 OS=Bos taurus GYG1 PE=4 SV=1 | 24 | 140 | 129 | 92 | 100 | 240 | 43 | 34 | 0.61 | 0.88 |
| G3MYZ3 | Afamin OS=Bos taurus AFM PE=1 SV=1 | 68 | 88 | 60 | 46 | 175 | 117 | 157 | 90 | 1.97 | 0.04 |
| Q1RMX7 | N-acetylneuraminate synthase OS=Bos taurus NANS PE=1 SV=1 | 90 | 112 | 172 | 54 | 90 | 42 | 101 | 141 | 0.69 | 0.74 |
| Q3T147 | Spliceosome RNA helicase DDX39B OS=Bos taurus DDX39B PE=2 SV=1 | 91 | 130 | . | 82 | 109 | 101 | 187 | 100 | 1.36 | 0.90 |
| F1N081 | Protein farnesyltransferase/geranylgeranyltransferase type-1 subunit alpha OS=Bos taurus FNTA PE=4 SV=1 | 92 | 98 | 66 | 158 | 182 | 88 | 69 | 46 | 0.66 | 0.87 |
| Q5E9E2 | Myosin regulatory light polypeptide 9 OS=Bos taurus MYL9 PE=2 SV=3 | 172 | 87 | 194 | 133 | 63 | 44 | 47 | 61 | 0.36 | 0.03 |
| P63243 | Receptor of activated protein C kinase 1 OS=Bos taurus RACK1 PE=2 SV=3 | 115 | 91 | 85 | 67 | . | 69 | 275 | 99 | 1.74 | 0.40 |
| O77482 | Interleukin-1 receptor antagonist protein OS=Bos taurus IL1RN PE=2 SV=1 | 49 | 118 | 43 | 105 | 52 | 221 | 148 | 64 | 1.17 | 0.33 |
| P81623 | Endoplasmic reticulum resident protein 29 OS=Bos taurus ERP29 PE=1 SV=2 | 55 | 96 | 77 | 90 | 126 | 96 | 122 | 137 | 1.47 | 0.07 |
| E1BD36 | Interleukin 6 signal transducer OS=Bos taurus IL6ST PE=4 SV=1 | 40 | 228 | 64 | 108 | 65 | 97 | 39 | 160 | 0.76 | 0.66 |
| Q2KIC7 | Serine/threonine-protein phosphatase OS=Bos taurus PPP6C PE=2 SV=2 | 133 | 75 | 138 | 100 | 110 | 99 | 85 | 61 | 0.76 | 0.27 |
| Q2HJA6 | Neutrophil cytosolic factor 4, 40kDa OS=Bos taurus NCF4 PE=2 SV=1 | 42 | 70 | 113 | 71 | 146 | 75 | 80 | 205 | 1.35 | 0.28 |
| E1BEX4 | Uroporphyrinogen decarboxylase OS=Bos taurus UROD PE=3 SV=1 | 218 | 106 | 131 | 124 | . | 122 | 75 | 24 | 0.51 | 0.30 |
| A5PKK0 | FAM151B protein OS=Bos taurus FAM151B PE=2 SV=1 | 125 | 115 | 114 | 88 | 87 | 98 | 92 | 82 | 0.81 | 0.05 |
| Q3ZBN0 | Costars family protein ABRACL OS=Bos taurus ABRACL PE=3 SV=1 | 153 | 74 | 143 | 91 | . | 76 | 111 | 151 | 1.39 | 0.75 |
| G1K147 | Protein SGT1 homolog OS=Bos taurus SUGT1 PE=4 SV=2 | 147 | 77 | 199 | 69 | . | 112 | 64 | 133 | 0.92 | 0.86 |
| P68401 | Platelet-activating factor acetylhydrolase IB subunit beta OS=Bos taurus PAFAH1B2 PE=1 SV=1 | 206 | 89 | 103 | 89 | 93 | 88 | 78 | 56 | 0.65 | 0.18 |
| F1N1D5 | Glucosylceramidase OS=Bos taurus GBA PE=3 SV=1 | 87 | 121 | 82 | 68 | 53 | 142 | 104 | 143 | 1.06 | 0.42 |
| A6QLI0 | Mammalian ependymin-related protein 1 OS=Bos taurus EPDR1 PE=2 SV=1 | 64 | 211 | 98 | 242 | . | 62 | 44 | 80 | 0.46 | 0.07 |
| Q3SZ16 | Isoamyl acetate-hydrolyzing esterase 1 homolog OS=Bos taurus IAH1 PE=2 SV=1 | 39 | 56 | 137 | 127 | . | 98 | 167 | 176 | 1.90 | 0.02 |
| Q3MHM0 | Amyloid beta (A4) protein-binding, family B, member 1 interacting protein OS=Bos taurus APBB1IP PE=2 SV=1 | 120 | 69 | 29 | 76 | 131 | 298 | 39 | 39 | 1.04 | 0.44 |
| P35705 | Thioredoxin-dependent peroxide reductase, mitochondrial OS=Bos taurus PRDX3 PE=1 SV=2 | 38 | 78 | 230 | 109 | . | 46 | 53 | 246 | 0.62 | 0.81 |
| Q5E987 | Proteasome subunit alpha type-5 OS=Bos taurus PSMA5 PE=1 SV=1 | 115 | 105 | 106 | 92 | 137 | 82 | 72 | 91 | 0.87 | 0.52 |
| A2VDV7 | Transmembrane protease, serine 2 OS=Bos taurus TMPRSS2 PE=2 SV=1 | 163 | 106 | 224 | 89 | . | 66 | 82 | 70 | 0.71 | 0.22 |
| A6QQP4 | SCARB2 protein OS=Bos taurus SCARB2 PE=1 SV=1 | 48 | 221 | 112 | 139 | . | 59 | 26 | 195 | 0.46 | 0.42 |
| A6QNZ7 | Keratin 10 (Epidermolytic hyperkeratosis; keratosis palmaris et plantaris) OS=Bos taurus KRT10 PE=2 SV=1 | 47 | 87 | 47 | 194 | 203 | 85 | 76 | 62 | 0.80 | 0.84 |
| Q32PB8 | 40S ribosomal protein S21 OS=Bos taurus RPS21 PE=3 SV=1 | 187 | 94 | 80 | 91 | . | 52 | 136 | 161 | 1.35 | 0.51 |
| P62992 | Ubiquitin-40S ribosomal protein S27a OS=Bos taurus RPS27A PE=1 SV=2 | 192 | 102 | 134 | 127 | . | 73 | 79 | 94 | 0.90 | 0.04 |
| F1MS56 | Phosphoacetylglucosamine mutase OS=Bos taurus PGM3 PE=3 SV=1 | 107 | 119 | 176 | 69 | 72 | 104 | 86 | 66 | 0.70 | 0.16 |
| P54149 | Mitochondrial peptide methionine sulfoxide reductase OS=Bos taurus MSRA PE=1 SV=2 | 170 | 69 | 149 | 168 | 43 | 69 | 81 | 52 | 0.40 | 0.07 |
| G3N3P6 | Cystatin OS=Bos taurus OX=9913 PE=3 SV=1 | 51 | 124 | . | 148 | . | 206 | 73 | 197 | . | 0.16 |
| A0A0A0MPA3 | Beta-defensin 6 OS=Bos taurus DEFB6 PE=4 SV=1 | . | . | 60 | 441 | . | 74 | 186 | 38 | . | 0.69 |
| P00974 | Pancreatic trypsin inhibitor OS=Bos taurus OX=9913 PE=1 SV=2 | 39 | . | 47 | 466 | . | 119 | 99 | 31 | . | 0.58 |
| Q29460 | Platelet-activating factor acetylhydrolase IB subunit gamma OS=Bos taurus PAFAH1B3 PE=1 SV=1 | 247 | 56 | 117 | 61 | 93 | 95 | 90 | 43 | 0.67 | 0.40 |
| Q56JW4 | Adenine phosphoribosyltransferase OS=Bos taurus APRT PE=2 SV=1 | 64 | 91 | 124 | 62 | 34 | 126 | 151 | 149 | 1.04 | 0.30 |
| F1N514 | CD5 antigen-like precursor OS=Bos taurus CD5L PE=1 SV=2 | 59 | 160 | 58 | 69 | 84 | 132 | 88 | 151 | 1.32 | 0.31 |
| A5D7A2 | GARS protein OS=Bos taurus GARS PE=1 SV=1 | 112 | 147 | 48 | 80 | 111 | 127 | 116 | 59 | 1.02 | 0.78 |
| G3X743 | Arginyl aminopeptidase OS=Bos taurus RNPEP PE=4 SV=1 | 159 | 111 | 148 | 73 | 37 | 88 | 83 | 102 | 0.50 | 0.26 |
| Q2KJ63 | Plasma kallikrein OS=Bos taurus KLKB1 PE=2 SV=1 | 46 | 90 | . | 71 | . | 186 | 274 | 133 | . | 0.14 |
| A6H744 | SUMO-conjugating enzyme UBC9 OS=Bos taurus UBE2I PE=2 SV=1 | 114 | 54 | 257 | 94 | 52 | 90 | 53 | 86 | 0.46 | 0.34 |
| Q17QC7 | Nectin cell adhesion molecule 2 OS=Bos taurus NECTIN2 PE=2 SV=1 | 52 | 170 | 104 | 106 | 44 | 80 | 70 | 173 | 0.74 | 0.66 |
| Q17QB3 | Acid ceramidase OS=Bos taurus ASAH1 PE=2 SV=3 | 76 | 117 | . | 85 | . | 167 | 270 | 85 | . | 0.50 |
| H9GW42 | Dipeptidyl peptidase 7 OS=Bos taurus DPP7 PE=4 SV=1 | 60 | 115 | 82 | 152 | 165 | 106 | 44 | 77 | 0.76 | 0.92 |
| P07456 | Insulin-like growth factor II OS=Bos taurus IGF2 PE=1 SV=4 | 32 | 166 | 85 | 139 | . | 187 | 81 | 110 | 1.26 | 0.82 |
| G3MYG3 | Complement C3d receptor 2 OS=Bos taurus CR2 PE=4 SV=1 | 61 | 194 | 123 | 126 | . | 106 | 36 | 155 | 0.66 | 0.34 |
| Q0IIM2 | ADP-ribosylation factor-like protein 6 OS=Bos taurus ARL6 PE=1 SV=1 | 135 | 96 | 86 | 114 | 31 | 85 | 167 | 87 | 0.54 | 0.71 |
| A0A140T866 | D-3-phosphoglycerate dehydrogenase OS=Bos taurus PHGDH PE=1 SV=1 | 154 | 139 | 167 | 61 | . | 83 | 107 | 90 | 1.02 | 0.42 |
| G3MX90 | Serine/threonine-protein phosphatase OS=Bos taurus OX=9913 PE=3 SV=1 | 174 | 74 | 107 | 126 | 30 | 75 | 109 | 104 | 0.44 | 0.33 |
| G5E5V1 | Uncharacterized protein OS=Bos taurus OX=9913 PE=4 SV=1 | 64 | 125 | 19 | 100 | 93 | 129 | 106 | 164 | 1.63 | 0.09 |
| F1MGK8 | Oncostatin M receptor OS=Bos taurus OSMR PE=4 SV=2 | 102 | 78 | 172 | 79 | . | 79 | 100 | 190 | 1.28 | 0.82 |
| Q3MHH4 | Glutamine--tRNA ligase OS=Bos taurus QARS PE=2 SV=1 | 154 | 91 | 208 | 76 | . | 87 | 76 | 109 | 0.90 | 0.57 |
| F1N0Y0 | Uridine phosphorylase OS=Bos taurus UPP1 PE=1 SV=1 | 14 | 42 | . | 310 | 44 | 134 | 201 | 55 | 0.64 | 0.72 |
| F1MJ11 | Plakophilin 4 OS=Bos taurus PKP4 PE=4 SV=2 | 42 | 93 | 53 | 242 | 79 | 65 | 87 | 140 | 0.93 | 0.69 |
| Q0VCY8 | Phosphoprotein enriched in astrocytes 15 OS=Bos taurus PEA15 PE=2 SV=1 | 310 | 88 | 294 | 21 | . | 25 | 29 | 33 | 0.28 | 0.33 |
| Q3T0F7 | Myotrophin OS=Bos taurus MTPN PE=1 SV=3 | 116 | 113 | 173 | 161 | 13 | 86 | 41 | 98 | 0.25 | 0.04 |
| Q32KR9 | Charged multivesicular body protein 1A OS=Bos taurus CHMP1A PE=2 SV=1 | 205 | 67 | 103 | 93 | 111 | 67 | 53 | 101 | 0.70 | 0.25 |
| Q148I1 | Proteasomal ATPase-associated factor 1 OS=Bos taurus PAAF1 PE=2 SV=1 | 68 | . | 149 | 131 | . | . | 38 | 413 | . | 0.74 |
| F1MMR5 | Tetratricopeptide repeat domain 38 OS=Bos taurus TTC38 PE=4 SV=1 | 39 | 60 | 82 | 62 | 164 | 101 | 181 | 112 | 2.18 | 0.03 |
| Q5E9J1 | Heterogeneous nuclear ribonucleoprotein F OS=Bos taurus HNRNPF PE=2 SV=3 | 64 | 122 | 244 | 165 | . | 60 | 66 | 80 | 0.51 | 0.09 |
| Q9N0T1 | Stanniocalcin-1 OS=Bos taurus STC1 PE=2 SV=2 | 47 | 249 | 54 | 93 | . | 95 | 67 | 195 | 1.02 | 0.87 |
| Q2TBG8 | Ubiquitin carboxyl-terminal hydrolase isozyme L3 OS=Bos taurus UCHL3 PE=2 SV=1 | 188 | 75 | 153 | 79 | 57 | 70 | 103 | 76 | 0.58 | 0.21 |
| F1MYG5 | Lamin A/C OS=Bos taurus LMNA PE=1 SV=1 | 55 | 67 | 58 | 337 | 43 | 64 | 77 | 100 | 0.62 | 0.40 |
| Q3T0G3 | AHA1, activator of heat shock 90kDa protein ATPase homolog 1 (Yeast) OS=Bos taurus AHSA1 PE=2 SV=1 | 100 | 112 | 47 | 76 | 147 | 109 | 145 | 63 | 1.24 | 0.29 |
| Q2KID4 | Dynein light chain 1, axonemal OS=Bos taurus DNAL1 PE=2 SV=1 | 40 | 26 | 20 | 262 | 347 | 45 | 38 | 22 | 0.31 | 0.83 |
| P62958 | Histidine triad nucleotide-binding protein 1 OS=Bos taurus HINT1 PE=1 SV=2 | 178 | 84 | 133 | 88 | 74 | 119 | 72 | 52 | 0.60 | 0.25 |
| A6QLG5 | 40S ribosomal protein S9 OS=Bos taurus RPS9 PE=2 SV=1 | 139 | 109 | 129 | 96 | 97 | 63 | 113 | 54 | 0.66 | 0.01 |
| Q2KJH6 | Serpin H1 OS=Bos taurus SERPINH1 PE=2 SV=1 | 16 | 45 | 27 | 78 | 498 | 48 | 44 | 42 | 1.17 | 0.41 |
| Q0III8 | RNASET2 protein (Fragment) OS=Bos taurus RNASET2 PE=2 SV=1 | 29 | 237 | 67 | 196 | . | 50 | 39 | 181 | 0.53 | 0.30 |
| F1N6W9 | Collagen type XVIII alpha 1 chain OS=Bos taurus COL18A1 PE=4 SV=2 | 118 | 100 | 252 | 83 | 45 | 79 | 62 | 62 | 0.43 | 0.15 |
| P25068 | Tracheal antimicrobial peptide OS=Bos taurus OX=9913 PE=1 SV=1 | 52 | 154 | 69 | 107 | . | 98 | 150 | 170 | 1.50 | 0.57 |
| F1MBP7 | Beta-defensin 10 OS=Bos taurus DEFB10 PE=4 SV=1 | . | . | 31 | 479 | . | 177 | 97 | 17 | . | 0.59 |
| A0A140T850 | Glutathione synthetase OS=Bos taurus GSS PE=3 SV=1 | 136 | 112 | 87 | 107 | 61 | 163 | 91 | 43 | 0.63 | 0.53 |
| F1N5S6 | Serine/threonine kinase 24 OS=Bos taurus STK24 PE=4 SV=1 | 73 | 90 | 144 | 188 | 56 | 70 | 49 | 132 | 0.57 | 0.08 |
| P80177 | Macrophage migration inhibitory factor OS=Bos taurus MIF PE=1 SV=6 | 298 | 62 | 92 | 107 | . | 85 | 98 | 58 | 1.13 | 0.78 |
| P20000 | Aldehyde dehydrogenase, mitochondrial OS=Bos taurus ALDH2 PE=1 SV=2 | . | . | 28 | 733 | . | 29 | 4 | 6 | . | 0.48 |
| G8JKV5 | 60S ribosomal protein L14 OS=Bos taurus RPL14 PE=4 SV=1 | 62 | 83 | 137 | 100 |  | 94 | 76 | 247 | 1.29 | 0.65 |
| Q3SZF0 | Acyl-CoA-binding domain-containing protein 7 OS=Bos taurus ACBD7 PE=3 SV=1 | 174 | 67 | 74 | 61 |  | 101 | 186 | 138 | 2.65 | 0.08 |
| G3N3S3 | Uncharacterized protein OS=Bos taurus OX=9913 PE=3 SV=1 | 143 | 122 | 124 | 88 | 56 | 90 | 112 | 64 | 0.63 | 0.10 |
| F1N2I5 | Carboxymethylenebutenolidase homolog OS=Bos taurus CMBL PE=4 SV=1 | 207 | 98 | 158 | 88 | 6 | 43 | 68 | 134 | 0.09 | 0.24 |
| Q3SZJ9 | Phosphomannomutase 2 OS=Bos taurus PMM2 PE=2 SV=1 | 96 | 55 | 67 | 81 | 225 | 80 | 110 | 85 | 1.49 | 0.17 |
| Q769I5 | Hepatocyte growth factor receptor OS=Bos taurus MET PE=2 SV=1 | 45 | 191 | 54 | 105 |  | 147 | 69 | 190 | 1.52 | 0.66 |
| F1MF56 | Matrix metalloproteinase-9 OS=Bos taurus MMP9 PE=3 SV=1 | 29 | 85 | 40 | 405 | 45 | 99 | 58 | 38 | 0.31 | 0.47 |
| Q9BDK2 | Allograft inflammatory factor 1 OS=Bos taurus AIF1 PE=2 SV=1 | 57 | 98 | 86 | 219 | 26 | 114 | 101 | 99 | 0.66 | 0.42 |
| Q3T0E0 | Copper transport protein ATOX1 OS=Bos taurus ATOX1 PE=3 SV=1 | 166 | 83 | 177 | 56 | 88 | 103 | 76 | 52 | 0.66 | 0.26 |
| E1BAD2 | Uncharacterized protein OS=Bos taurus LOC509956 PE=3 SV=1 | 9 | 96 | 26 | 315 | 186 | 75 | 69 | 24 | 0.27 | 0.83 |
| Q9TS87 | Transgelin OS=Bos taurus TAGLN PE=1 SV=4 | 47 | 127 | 229 | 28 | 47 | 83 | 125 | 115 | 0.87 | 0.73 |
| G3N132 | Beta-centractin OS=Bos taurus ACTR1B PE=3 SV=1 | 167 | 76 | 131 | 98 |  | 97 | 133 | 97 | 1.44 | 0.39 |
| Q08DS7 | AP-1 complex subunit beta-1 OS=Bos taurus AP2B1 PE=2 SV=1 | 47 | 180 | 48 | 126 | 208 | 71 | 57 | 64 | 0.72 | 0.99 |
| E1BN63 | WNK lysine deficient protein kinase 1 OS=Bos taurus WNK1 PE=4 SV=2 | 39 | 97 | 76 | 76 | 249 | 116 | 60 | 88 | 1.28 | 0.36 |
| Q0VC83 | Hexosyltransferase OS=Bos taurus B3GNT8 PE=2 SV=1 | 59 | 193 | 72 | 157 | 25 | 109 | 34 | 152 | 0.54 | 0.09 |
| F1N2T0 | Heterogeneous nuclear ribonucleoprotein D like OS=Bos taurus HNRNPDL PE=1 SV=1 | 55 | 88 | 71 | 306 | 35 | 71 | 71 | 103 | 0.59 | 0.30 |
| Q0II48 | Bcl-2-like protein 15 OS=Bos taurus BCL2L15 PE=2 SV=1 | 23 | 58 | 48 | 339 | 71 | 127 | 98 | 38 | 0.39 | 0.73 |
| F1MP48 | T-complex protein 1 subunit zeta-2 OS=Bos taurus CCT6B PE=3 SV=1 | 107 | 97 | 156 | 82 | 136 | 92 | 75 | 56 | 0.74 | 0.43 |
| E1BE25 | Filamin C OS=Bos taurus FLNC PE=1 SV=1 | 173 | 69 | 132 | 177 | 58 | 42 | 94 | 55 | 0.43 | 0.06 |
| A8DC37 | Fc-gamma-RII-D OS=Bos taurus FCGR2A PE=1 SV=1 | 12 | 184 | 89 | 65 | 63 | 110 | 42 | 235 | 0.95 | 0.68 |
| E1BEM3 | Uncharacterized protein OS=Bos taurus CDV3 PE=1 SV=2 | 198 | 75 | 154 | 73 | 62 | 67 | 82 | 89 | 0.57 | 0.24 |
| A4IFL4 | PPARD protein OS=Bos taurus PPARD PE=2 SV=1 | 34 | 99 | 52 | 70 | 219 | 98 | 105 | 124 | 1.80 | 0.16 |
| Q29RL2 | Cysteine and histidine-rich domain-containing protein 1 OS=Bos taurus CHORDC1 PE=2 SV=1 | 203 | 95 | 89 | 67 | 52 | 87 | 105 | 103 | 0.62 | 0.57 |
| F1N412 | Acetoacetyl-CoA synthetase OS=Bos taurus AACS PE=4 SV=1 | 185 | 78 | 56 | 79 | 53 | 163 | 170 | 17 | 0.45 | 0.99 |
| A7MBH9 | G protein subunit alpha i2 OS=Bos taurus GNAI2 PE=2 SV=1 | 53 | 94 | 100 | 206 | . | 79 | 113 | 156 | 1.18 | 0.44 |
| Q2T9Y6 | Glutamate--cysteine ligase regulatory subunit OS=Bos taurus GCLM PE=2 SV=1 | 202 | 93 | 169 | 56 | 50 | 93 | 99 | 38 | 0.49 | 0.18 |
| Q5E964 | 26S proteasome non-ATPase regulatory subunit 13 OS=Bos taurus PSMD13 PE=2 SV=1 | 109 | 119 | 118 | 69 | 161 | 75 | 80 | 69 | 0.84 | 0.75 |
| F1MJQ1 | Ras-related protein Rab-7a OS=Bos taurus RAB7A PE=1 SV=1 | 68 | 104 | 73 | 252 | 60 | 106 | 80 | 57 | 0.53 | 0.39 |
| Q3ZBI3 | Ribonuclease H2 subunit B OS=Bos taurus RNASEH2B PE=2 SV=2 | . | 158 | 87 | 331 | . | 153 | 29 | 43 | 0.34 | 0.31 |
| A6QLY4 | Isochorismatase domain-containing protein 1 OS=Bos taurus ISOC1 PE=2 SV=1 | 144 | 94 | 96 | 94 | 117 | 94 | 91 | 71 | 0.87 | 0.12 |
| A7MB07 | ADAM metallopeptidase with thrombospondin type 1 motif 1 OS=Bos taurus ADAMTS1 PE=2 SV=1 | 50 | 168 | 49 | 86 | 123 | 167 | 58 | 99 | 1.28 | 0.26 |
| Q0VFX9 | Gamma-glutamylaminecyclotransferase OS=Bos taurus GGACT PE=2 SV=1 | 139 | 112 | 78 | 73 | 253 | 51 | 54 | 40 | 0.67 | 0.98 |
| Q0VCQ6 | Programmed cell death 10 OS=Bos taurus PDCD10 PE=2 SV=1 | 68 | 45 | 300 | 84 | 69 | 184 | 29 | 20 | 0.25 | 0.61 |
| A7Z024 | Protein XRP2 OS=Bos taurus RP2 PE=2 SV=1 | 52 | 115 | 117 | 192 | 82 | 77 | 79 | 87 | 0.69 | 0.27 |
| F1MM86 | Complement component C6 OS=Bos taurus C6 PE=4 SV=1 | 44 | 58 | 72 | 43 | 323 | 91 | 98 | 71 | 1.90 | 0.24 |
| P80311 | Peptidyl-prolyl cis-trans isomerase B OS=Bos taurus PPIB PE=1 SV=4 | 122 | 84 | 157 | 135 | 22 | 56 | 82 | 143 | 0.40 | 0.14 |
| F1MK08 | Tripeptidyl-peptidase 1 OS=Bos taurus TPP1 PE=4 SV=2 | 57 | 121 | 58 | 95 | 110 | 181 | 89 | 89 | 1.37 | 0.10 |
| Q3ZCH9 | Haloacid dehalogenase-like hydrolase domain-containing protein 2 OS=Bos taurus HDHD2 PE=2 SV=1 | 193 | 87 | 174 | 80 | . | 69 | 115 | 81 | 1.07 | 0.29 |
| Q3MHR3 | Dynein light chain 2, cytoplasmic OS=Bos taurus DYNLL2 PE=3 SV=1 | 165 | 77 | 135 | 111 | 43 | 78 | 86 | 105 | 0.54 | 0.22 |
| A2VE29 | Inter-alpha-trypsin inhibitor heavy chain H5 OS=Bos taurus ITIH5 PE=2 SV=1 | 29 | 266 | 66 | 141 | 64 | 54 | 27 | 154 | 0.46 | 0.43 |
| F1N719 | Serine/threonine-protein phosphatase OS=Bos taurus PPP5C PE=3 SV=1 | 181 |  | 110 | 104 | 160 | 72 | 82 | 91 | 1.11 | 0.04 |
| G3MYW9 | Deoxyribonuclease OS=Bos taurus DNASE1 PE=3 SV=1 | 11 | 107 | 48 | 438 | 34 | 123 | 17 | 23 | 0.17 | 0.40 |
| A6H7G2 | Drebrin-like protein OS=Bos taurus DBNL PE=2 SV=1 | 171 | 56 | 142 | 114 | 76 | 66 | 62 | 114 | 0.63 | 0.22 |
| A0JN39 | Coatomer subunit beta OS=Bos taurus COPB1 PE=1 SV=1 | 63 | 84 | 64 | 214 | 224 | 54 | 37 | 60 | 0.56 | 0.85 |
| Q2KIM0 | Tissue alpha-L-fucosidase OS=Bos taurus FUCA1 PE=2 SV=1 | 359 | 187 | 50 | 50 | . | 39 | 62 | 55 | 0.62 | 0.49 |
| P20456 | Inositol monophosphatase 1 OS=Bos taurus IMPA1 PE=1 SV=1 | 187 | 87 | 115 | 126 | . | 116 | 114 | 55 | 0.99 | 0.68 |
| A6QP30 | CPN2 protein OS=Bos taurus CPN2 PE=2 SV=1 | 87 | . | 84 | 112 | 127 | 149 | 151 | 90 | 1.61 | 0.39 |
| A0A0A0MP88 | Uncharacterized protein OS=Bos taurus OX=9913 PE=3 SV=1 | 8 | 41 | 139 | . | 123 | 41 | 67 | 381 | 1.27 | 0.82 |
| F1MX51 | Far upstream element-binding protein 1 OS=Bos taurus FUBP1 PE=1 SV=2 | 103 | 74 | 209 | 190 | 31 | 79 | 55 | 59 | 0.35 | 0.09 |
| P32592 | Integrin beta-2 OS=Bos taurus ITGB2 PE=1 SV=1 | 16 | 36 | 24 | 31 | 488 | 49 | 73 | 84 | 2.75 | 0.27 |
| Q3SZ18 | Hypoxanthine-guanine phosphoribosyltransferase OS=Bos taurus HPRT1 PE=2 SV=3 | 256 | 46 | 148 | 78 | 57 | 57 | 80 | 78 | 0.49 | 0.28 |
| Q58DU7 | SH3 domain-binding glutamic acid-rich-like protein OS=Bos taurus SH3BGRL PE=3 SV=1 | 129 | 75 | 276 | 105 | 36 | 63 | 38 | 78 | 0.30 | 0.17 |
| F1N269 | Uncharacterized protein OS=Bos taurus OX=9913 PE=4 SV=2 | 99 | 82 | 212 | 121 | . | 67 | 52 | 167 | 0.66 | 0.55 |
| A6QPS1 | PPCS protein OS=Bos taurus PPCS PE=2 SV=1 | 145 | 98 | 91 | 103 | 48 | 107 | 113 | 96 | 0.69 | 0.55 |
| A4FV69 | SERPINB5 protein OS=Bos taurus SERPINB5 PE=1 SV=1 | 32 | 157 | 22 | 96 | 388 | 40 | 28 | 36 | 0.54 | 0.69 |
| F1MZC0 | Aflatoxin B1 aldehyde reductase member 2 OS=Bos taurus AKR7A2 PE=1 SV=1 | 15 | 117 | 27 | 36 | 316 | 152 | 81 | 54 | 2.20 | 0.22 |
| F1MDM6 | DCN1-like protein OS=Bos taurus DCUN1D1 PE=4 SV=2 | 239 | 69 | 178 | 69 | 32 | 63 | 52 | 98 | 0.31 | 0.25 |
| Q5E983 | Elongation factor 1-beta OS=Bos taurus EEF1B PE=2 SV=3 | 105 | 106 | 95 | 98 | 60 | 76 | 158 | 103 | 0.85 | 0.95 |
| P00586 | Thiosulfate sulfurtransferase OS=Bos taurus TST PE=1 SV=3 | 131 | 70 | 124 | 97 | 59 | 154 | 101 | 64 | 0.74 | 0.77 |
| Q29RY7 | Fibroleukin OS=Bos taurus FGL2 PE=2 SV=1 | 53 | 165 | 63 | 103 | . | 169 | 136 | 111 | 1.69 | 0.33 |
| E1B925 | AT-rich interaction domain 1A OS=Bos taurus ARID1A PE=4 SV=2 | 269 | 19 | . | 173 | 125 | 34 | 174 | 7 | 0.14 | 0.23 |
| G3MYE2 | Uncharacterized protein OS=Bos taurus OX=9913 PE=3 SV=1 | 178 | 70 | 148 | 90 | . | 79 | 130 | 105 | 1.39 | 0.87 |
| P79342 | Protein S100-A13 OS=Bos taurus S100A13 PE=3 SV=2 | 152 | 102 | 124 | 92 | 22 | 88 | 95 | 127 | 0.39 | 0.40 |
| P61603 | 10 kDa heat shock protein, mitochondrial OS=Bos taurus HSPE1 PE=3 SV=2 | 96 | 129 | 72 | 206 | 42 | 59 | 123 | 73 | 0.51 | 0.27 |
| E1BJ20 | Uncharacterized protein OS=Bos taurus OX=9913 PE=4 SV=1 | 200 | 84 | 209 | 87 | 35 | 59 | 48 | 80 | 0.32 | 0.13 |
| Q17QS1 | Sorting nexin-24 OS=Bos taurus SNX24 PE=2 SV=1 | . | 137 | 137 | 146 | 70 | 229 | 46 | 37 | 0.53 | 0.63 |
| P48427 | Tubulin-specific chaperone A OS=Bos taurus TBCA PE=1 SV=3 | 202 | 87 | 165 | 76 | 48 | 94 | 75 | 53 | 0.46 | 0.17 |
| E1BFJ4 | Ring finger protein 17 OS=Bos taurus RNF17 PE=4 SV=1 | 276 | 53 | 24 | 249 | 40 | 24 | 113 | 21 | 0.19 | 0.29 |
| Q0P5I9 | C-X-C motif chemokine ligand 12 OS=Bos taurus CXCL12 PE=2 SV=1 | 81 | 134 | 77 | 173 | . | 69 | 43 | 223 | 0.89 | 0.68 |
| F1N0F0 | Uncharacterized protein OS=Bos taurus LOC782366 PE=3 SV=2 | . | . | . | 8 | 739 | 19 | 14 | 20 | . | #DIV/0! |
| Q9N0T5 | Eukaryotic translation initiation factor 4E OS=Bos taurus EIF4E PE=2 SV=2 | 204 | 82 | 101 | 71 | 72 | 80 | 116 | 75 | 0.71 | 0.47 |
| F1N3U5 | Vanin 2 OS=Bos taurus VNN2 PE=4 SV=2 | 31 | 69 | 42 | 131 | 52 | 52 | 111 | 313 | 1.46 | 0.24 |
| G5E5K1 | Prefoldin subunit 3 OS=Bos taurus VBP1 PE=3 SV=1 | 181 | 101 | 107 | 87 | 68 | 99 | 92 | 66 | 0.65 | 0.23 |
| P80025 | Lactoperoxidase OS=Bos taurus LPO PE=1 SV=1 | 26 | 106 | 50 | 557 | . | 27 | 13 | 21 | 0.11 | 0.31 |
| E1BF81 | Corticosteroid-binding globulin OS=Bos taurus SERPINA6 PE=3 SV=1 | 46 | 128 | 47 | 61 | 80 | 130 | 171 | 137 | 1.75 | 0.11 |
| E1BHA5 | Uncharacterized protein OS=Bos taurus OX=9913 PE=4 SV=1 | 173 | 126 | 78 | 109 | 90 | 88 | 103 | 33 | 0.54 | 0.18 |
| P08037 | Beta-1,4-galactosyltransferase 1 OS=Bos taurus B4GALT1 PE=1 SV=3 | 61 | 266 | 33 | 152 | 60 | 114 | 50 | 65 | 0.63 | 0.25 |
| G3MY11 | MARCKS like 1 OS=Bos taurus MARCKSL1 PE=4 SV=1 | 78 | 45 | . | 154 | 94 | 104 | 260 | 65 | 1.11 | 0.93 |
| G3N2L2 | Reticulocalbin 1 OS=Bos taurus RCN1 PE=1 SV=1 | 47 | 197 | 105 | 85 | 136 | 57 | 62 | 111 | 0.64 | 0.75 |
| F1MMK2 | Glucose-6-phosphate 1-dehydrogenase OS=Bos taurus G6PD PE=1 SV=1 | 161 | 68 | 99 | 118 | 59 | 69 | 118 | 109 | 0.71 | 0.46 |
| F1MG94 | WD repeat domain 82 OS=Bos taurus WDR82 PE=4 SV=1 | 75 | 291 | 80 | 83 | . | 164 | 55 | 54 | 0.84 | 0.21 |
| Q2TBX4 | Heat shock 70 kDa protein 13 OS=Bos taurus HSPA13 PE=2 SV=1 | 59 | 63 | 35 | 161 | 94 | 147 | 146 | 96 | 1.35 | 0.36 |
| E1BI01 | Mitogen-activated protein kinase kinase 5 OS=Bos taurus MAP2K5 PE=3 SV=2 | 15 | 38 | 141 | 202 | 49 | 32 | 40 | 283 | 0.70 | 0.96 |
| F1N2N5 | Myristoylated alanine-rich C-kinase substrate OS=Bos taurus MARCKS PE=4 SV=1 | 41 | 71 | 40 | 185 | 106 | 84 | 185 | 88 | 1.13 | 0.58 |
| F1MX87 | Complement C8 alpha chain OS=Bos taurus C8A PE=4 SV=1 | 161 | 67 | 133 | 60 | 184 | 91 | 52 | 54 | 0.76 | 0.71 |
| F1ML72 | Ribosomal protein L34 OS=Bos taurus RPL34 PE=4 SV=1 | 115 | 71 | 130 | 79 | 124 | 126 | 104 | 52 | 0.94 | 0.89 |
| Q0P5D0 | Peptidyl-prolyl cis-trans isomerase H OS=Bos taurus PPIH PE=2 SV=1 | 300 | 82 | 211 | 47 | . | 43 | 62 | 56 | 0.65 | 0.33 |
| A2VE79 | Diphosphoinositol polyphosphate phosphohydrolase 1 OS=Bos taurus NUDT3 PE=2 SV=1 | 144 | 100 | 150 | 135 | . | 90 | 90 | 91 | 0.94 | 0.12 |
| Q2KJ25 | 26S proteasome non-ATPase regulatory subunit 12 OS=Bos taurus PSMD12 PE=2 SV=3 | 115 | 91 | 102 | 103 | 119 | 86 | 106 | 79 | 0.93 | 0.50 |
| Q29451 | Lysosomal alpha-mannosidase OS=Bos taurus MAN2B1 PE=1 SV=4 | 38 | 82 | 43 | 88 | 234 | 99 | 98 | 118 | 1.84 | 0.17 |
| Q17QQ2 | Thiopurine S-methyltransferase OS=Bos taurus TPMT PE=2 SV=1 | 72 | 99 | 79 | 137 | 185 | 88 | 85 | 56 | 0.81 | 0.88 |
| P80724 | Brain acid soluble protein 1 OS=Bos taurus BASP1 PE=1 SV=3 | 211 | 98 | 97 | 135 | 90 | 142 | 17 | 11 | 0.19 | 0.17 |
| F1MLX9 | Guanine deaminase OS=Bos taurus GDA PE=1 SV=2 | 46 | 144 | 63 | 131 | 78 | 108 | 150 | 81 | 1.01 | 0.81 |
| E1BN82 | Mitogen-activated protein kinase 8 interacting protein 3 isoform 1 OS=Bos taurus MAPK8IP3 PE=4 SV=2 | 106 | 89 | 245 | 162 | . | 108 | 34 | 56 | 0.37 | 0.27 |
| P33672 | Proteasome subunit beta type-3 OS=Bos taurus PSMB3 PE=1 SV=3 | 159 | 164 | 94 | 120 | 44 | 81 | 89 | 49 | 0.44 | 0.06 |
| F1MRZ6 | Tenascin C OS=Bos taurus TNC PE=4 SV=2 |  | 79 | 85 | 30 | 413 | 27 | 84 | 83 | 0.93 | 1.00 |
| P55918 | Microfibril-associated glycoprotein 4 OS=Bos taurus MFAP4 PE=1 SV=2 | 30 | 245 | 80 | 70 | 85 | 147 | 72 | 74 | 0.98 | 0.74 |
| A4IFR0 | C-X-C motif chemokine 17 OS=Bos taurus CXCL17 PE=3 SV=1 | 22 | 111 | 93 | 87 | 90 | 333 | 28 | 36 | 0.63 | 0.56 |
| F1N6Z0 | 26S proteasome non-ATPase regulatory subunit 5 OS=Bos taurus PSMD5 PE=4 SV=1 | 85 | 58 | . | 87 | . | 106 | 420 | 44 | . | 0.97 |
| G5E5M5 | Membrane-associated progesterone receptor component 1 OS=Bos taurus PGRMC1 PE=3 SV=1 | 76 | 54 | 32 | 201 | 83 | 66 | 124 | 163 | 1.24 | 0.55 |
| A0A140T892 | Protein-glutamine gamma-glutamyltransferase 2 OS=Bos taurus TGM2 PE=1 SV=1 | 285 | 93 | 169 | 62 | 38 | 69 | 38 | 47 | 0.27 | 0.15 |
| A0A140T853 | Isopentenyl-diphosphate Delta-isomerase 1 OS=Bos taurus IDI1 PE=4 SV=1 | 51 | 83 | 137 | 195 | . | 76 | 122 | 136 | 1.10 | 0.24 |
| G3MY01 | C7orf55-LUC7L2 readthrough OS=Bos taurus C7orf55-LUC7L2 PE=4 SV=1 | 109 | 120 | 99 | 71 | 153 | 85 | 90 | 72 | 0.95 | 0.98 |
| G3N3M2 | TBC1 domain family member 4 OS=Bos taurus TBC1D4 PE=4 SV=1 | 325 | 30 | 242 | 16 | 45 | 46 | 48 | 49 | 0.30 | 0.26 |
| Q3T0Y5 | Proteasome subunit alpha type-2 OS=Bos taurus PSMA2 PE=1 SV=3 | 99 | 146 | 152 | 95 | 117 | 80 | 59 | 53 | 0.57 | 0.15 |
| E1BPQ9 | Immunoglobulin superfamily member 5 OS=Bos taurus IGSF5 PE=4 SV=1 | 81 | 305 | 43 | 192 | 12 | 100 | 22 | 46 | 0.25 | 0.07 |
| F1MHS5 | Protein S100-A9 OS=Bos taurus S100A9 PE=1 SV=2 | 23 | 53 | 55 | 426 | 13 | 92 | 58 | 80 | 0.46 | 0.45 |
| F1MHM5 | Tyrosine--tRNA ligase OS=Bos taurus YARS PE=3 SV=1 | 73 | 50 | 37 | 61 | 58 | 366 | 72 | 83 | 1.51 | 0.33 |
| F1MLV3 | Thioredoxin like 1 OS=Bos taurus TXNL1 PE=1 SV=1 | 240 | 95 | 85 | 71 | 51 | 83 | 114 | 61 | 0.51 | 0.42 |
| Q0P5D6 | Retinoic acid receptor responder (Tazarotene induced) 1 OS=Bos taurus RARRES1 PE=2 SV=1 | 80 | 50 | 500 | 86 | . | 70 | 8 | 6 | 0.05 | 0.36 |
| A6QM11 | Pro-thyrotropin-releasing hormone OS=Bos taurus TRH PE=2 SV=1 | 17 | 71 | 76 | 49 | 176 | 68 | 78 | 266 | 1.73 | 0.19 |
| F1MXE0 | Heart development protein with EGF like domains 1 OS=Bos taurus HEG1 PE=4 SV=2 | 44 | 152 | 46 | 120 | 167 | 58 | 46 | 167 | 0.87 | 0.70 |
| C4T8B4 | Pentaxin OS=Bos taurus CRP PE=2 SV=1 | 32 | 57 | 61 | 27 | 419 | 101 | 63 | 42 | 1.78 | 0.31 |
| F1MHH9 | Low affinity immunoglobulin gamma Fc region receptor II OS=Bos taurus FCGR2B PE=4 SV=2 | 150 | 227 | 27 | 49 | 65 | 175 | 81 | 26 | 0.69 | 0.44 |
| P25975 | Cathepsin L1 OS=Bos taurus CTSL PE=1 SV=3 | 44 | 105 | 111 | 105 | 91 | 67 | 49 | 228 | 0.83 | 0.71 |
| Q0P565 | HD domain-containing protein 2 OS=Bos taurus HDDC2 PE=2 SV=1 | 174 | 76 | 83 | 66 | 75 | 78 | 119 | 130 | 0.89 | 0.98 |
| E1BNG1 | AarF domain containing kinase 2 OS=Bos taurus ADCK2 PE=4 SV=1 | 20 | 22 | 16 | 37 | 659 | 23 | 8 | 15 | 0.71 | 0.42 |
| Q2NL29 | Inositol-3-phosphate synthase 1 OS=Bos taurus ISYNA1 PE=2 SV=1 | . | 93 | 104 | 83 | . | 51 | 59 | 410 | 1.06 | 0.58 |
| E1B9R5 | Dynein axonemal heavy chain 8 OS=Bos taurus DNAH8 PE=4 SV=2 | . | 24 | 100 | . | 201 | 33 | 35 | 408 | . | 0.59 |
| G3N1S7 | Uncharacterized protein OS=Bos taurus OX=9913 PE=4 SV=1 | 234 | 56 | 194 | 64 | . | 46 | 98 | 108 | 1.05 | 0.66 |
| Q0P5E8 | Chromatin accessibility complex 1 OS=Bos taurus CHRAC1 PE=2 SV=1 | 55 | 91 | 247 | 66 | 101 | 207 | 20 | 13 | 0.22 | 0.72 |
| Q3T0Y1 | Ubiquitin thioesterase OS=Bos taurus OTUB1 PE=1 SV=1 | 244 | 100 | 117 | 72 | . | 76 | 111 | 80 | 1.23 | 0.53 |
| G3N2Y1 | Uncharacterized protein OS=Bos taurus OX=9913 PE=4 SV=1 | 58 | 124 | 96 | 75 | 149 | 155 | 71 | 70 | 1.11 | 0.43 |
| E1BIU7 | Protein tyrosine phosphatase, non-receptor type 23 OS=Bos taurus PTPN23 PE=4 SV=2 | 16 | 57 | 30 | 38 | 310 | 112 | 94 | 143 | 3.49 | 0.10 |
| A1A4J1 | ATP-dependent 6-phosphofructokinase, liver type OS=Bos taurus PFKL PE=2 SV=1 | 114 | 94 | 94 | 128 | 101 | 93 | 76 | 102 | 0.86 | 0.06 |
| A7YWC4 | ATPase family AAA domain-containing protein 3 OS=Bos taurus ATAD3 PE=2 SV=1 | 21 | 61 | 299 | 168 | 61 | 185 | 4 | 3 | 0.03 | 0.49 |
| E1BEQ4 | Uncharacterized protein OS=Bos taurus LOC505658 PE=3 SV=2 | 23 | 47 | 30 | 352 | 24 | 128 | 145 | 51 | 0.47 | 0.80 |
| G3MYX8 | Tyrosine-protein kinase receptor OS=Bos taurus ROS1 PE=3 SV=1 | 70 | 161 | 118 | 218 | . | 65 | 34 | 135 | 0.53 | 0.00 |
| E1BK63 | Ribosomal protein L15 OS=Bos taurus OX=9913 PE=3 SV=1 | 90 | 96 | 133 | 134 | 127 | 116 | 68 | 37 | 0.56 | 0.48 |
| F1MI67 | BPI fold containing family B member 2 OS=Bos taurus BPIFB2 PE=4 SV=2 | 88 | 50 | 185 | 204 | . | 41 | 32 | 200 | #VALUE! | 0.38 |
