# Supplementary material for: Pulmonary and systemic responses to aerosolized lysate of Staphylococcus aureus and Escherichia coli in calves
Source: BMC Vet Res. 2020 May 29;16:168. doi: 10.1186/s12917-020-02383-7 (PMC7260748; doi:10.1186/s12917-020-02383-7)
Supplement: Supplementary file 7 — Additional file 7. PANTHER classification of 19 upregulated proteins and 26 downregulated proteins in bronchoalveolar lavage fluid following aerosolization of bacterial lysate in 4 calves. [file 12917_2020_2383_MOESM7_ESM.docx]

Additional file 7. PANTHER classification of 19 upregulated proteins and 26 downregulated proteins in bronchoalveolar lavage fluid following aerosolization of bacterial lysate in 4 calves.

A) PANTHER classification of 19 proteins that were increased 24 hours after aerosolization of bacterial lysate in 4 calves.

| **Protein ID** | **Gene Name** | **PANTHER Protein Class** |
| --- | --- | --- |
| **P82943** | Regakine-1 | Chemokine (PC00074) |
| **E1BH06** | Uncharacterized protein | Complement component (PC00078); cytokine (PC00083); serine protease inhibitor (PC00204) |
| **P01888** | Beta-2-microglobulin | Major histocompatibility complex antigen (PC00149) |
| **Q29437** | Primary amine oxidase, liver isozyme |  |
| **Q3ZBS7** | Vitronectin |  |
| **G3MYZ3** | Afamin | Transfer/carrier protein (PC00219) |
| **G3N1U4** | Serpin A3-3 | Serine protease inhibitor (PC00204) |
| **F1MMR5** | Tetratricopeptide repeat domain 38 |  |
| **F1MJK3** | Uncharacterized protein | Complement component (PC00078); cytokine (PC00083); serine protease inhibitor (PC00204) |
| **Q0V8R6** | Beta-hexosaminidase subunit alpha | Glycosidase (PC00110) |
| **F1N076** | Ceruloplasmin | Oxidase (PC00175) |
| **Q2KIS7** | Tetranectin | Extracellular matrix structural protein (PC00103) |
| **A5PJT7** | ECM1 protein |  |
| **Q28085** | Complement factor H |  |
| **Q7SIH1** | Alpha-2-macroglobulin | Complement component (PC00078); cytokine(PC00083); serine protease inhibitor (PC00204) |
| **Q29443** | Serotransferrin (transferrin); ortholog | Receptor (PC00197); serine protease (PC00203); transfer/carrier protein (PC00219) |
| **F1MCF8** | Uncharacterized protein | Immunoglobulin (PC00123) |
| **P81187** | Complement factor B |  |
| **F1MVK1** | Uncharacterized protein | Complement component (PC00078); cytokine (PC00083); serine protease inhibitor (PC00204) |

B) PANTHER classification of 26 proteins that were reduced 24 hours after aerosolization of bacterial lysate in 4 calves.

| **Accession** | **Gene Name** | **PANTHER Protein Class** |
| --- | --- | --- |
| **A6QLG5** | 40S ribosomal protein S9 | Ribosomal protein (PC00202) |
| **Q3T0S6** | 60S ribosomal protein L8 | Ribosomal protein (PC00202) |
| **P04272** | Annexin A2 |  |
| **A0A140T843** | Beta-2-glycoprotein 1 |  |
| **F1MG05** | Elongation factor 1-gamma |  |
| **P11116** | Galectin-1 | Cell adhesion molecule (PC00069);signaling molecule (PC00207) |
| **Q0IIM3** | Heat shock protein 105 kDa |  |
| **Q3ZC44** | Heterogeneous nuclear ribonucleoprotein A/B |  |
| **Q5E9J1** | Heterogeneous nuclear ribonucleoprotein F | Ribosomal protein(PC00202) |
| **Q3T0D0** | Heterogeneous nuclear ribonucleoprotein K | Enzyme modulator (PC00095); mrna splicing factor (PC00148); ribonucleoprotein (PC00201); serine protease (PC00203) |
| **G3MXB5** | IG alpha chain C region | Immunoglobulin receptor superfamily |
| **F1MC48** | IQ motif containing gtpase activating protein 1 | G-protein modulator (PC00022) |
| **A6QLI0** | Mammalian ependymin-related protein 1 |  |
| **Q5E9E2** | Myosin regulatory light polypeptide 9 | Actin family cytoskeletal protein (PC00041); calmodulin (PC00061) |
| **Q3T0F7** | Myotrophin |  |
| **Q2YDE4** | Proteasome subunit alpha type-6 |  |
| **A5PKK0** | Protein fam151b |  |
| **Q1RMR9** | Protein kinase C and casein kinase substrate in neurons 2 | Actin family cytoskeletal protein (PC00041); membrane trafficking regulatory protein (PC00151) |
| **G3MYX8** | Proto-oncogene tyrosine-protein kinase ROS |  |
| **F1MM32** | Sulfhydryl oxidase | Oxidase (PC00175) |
| **F1MSE7** | Sushi domain containing 2 |  |
| **Q3SZA6** | Syndecan binding protein | Membrane trafficking regulatory protein (PC00151) |
| **G3MYX8** | Tyrosine-protein kinase receptor |  |
| **G3MXB5** | Uncharacterized protein | Immunoglobulin receptor superfamily(PC00124) |
| **Q3T0M0** | Vacuolar protein sorting-associated protein 29 | Vesicle coat protein(PC00235) |
| **P31404** | V-type proton atpase catalytic subunit A | ATP synthase(PC00002);DNA binding protein(PC00009);anion channel(PC00049);ligand-gated ion channel(PC00141);protease(PC00190) |

C) PANTHER protein class for bronchoalveolar lavage proteins. Mass spectrometry identified proteins that were increased (A) or decreased (B) in bronchoalveolar lavage 24 hours after aerosolization of bacterial lysate. Included are proteins that were different from baseline, P ≤0.10, paired t-test. Protein classes that contained only one protein were excluded.
