# Supplementary material for: Pulmonary and systemic responses to aerosolized lysate of Staphylococcus aureus and Escherichia coli in calves
Source: BMC Vet Res. 2020 May 29;16:168. doi: 10.1186/s12917-020-02383-7 (PMC7260748; doi:10.1186/s12917-020-02383-7)
Supplement: Supplementary file 8 — Additional file 8. Reactome pathway analysis for differently expressed proteins in bronchoalveolar lavage fluid in 4 calves. [file 12917_2020_2383_MOESM8_ESM.docx]

Additional File 8. Reactome pathway analysis for differently expressed proteins in bronchoalveolar lavage fluid in 4 calves. For each Reactome pathway, the listed proteins were increased (P<0.05, or P>0.05 but <0.10) following aerosolization of bacterial lysate compared to baseline levels.

| Reactome pathway | P<0.05 | 0.05<P<0.10 |
| --- | --- | --- |
| Complement  (activation of C3 and C5 / regulation of Complement cascade) | Uncharacterized protein, C4a gene (E1BH06)  Vitronectin (Q3ZBS7)  Uncharacterized protein (F1MVK1) | Complement component C9 (Q3MHN2)  Complement factor I (F1N4M7) |
| Platelet responses (platelet degranulation/response to elevated platelet cytosolic Ca2+/platelet signaling and activation) | ECM1 protein (A5PJT7)  Tetranectin (Q2KIS7)  Alpha-2-macroglobulin (Q7SIH1) | Inter-alpha-trypsin inhibitor heavy chain H4 (F1MMD7) |
| Post-translational protein phosphorylation | Uncharacterized protein (F1MVK1)  Ceruloplasmin (F1N076) | Kininogen-1 (A0A140T8C8) |
| Regulation of Insulin-like Growth Factor transport and uptake by IGF Binding Proteins | Uncharacterized protein (F1MVK1)  Ceruloplasmin (F1N076) | Kininogen-1 (A0A140T8C8) |

| Pathway name | Entities | | | | Reactions | |
| --- | --- | --- | --- | --- | --- | --- |
|  | found | ratio | p-value | FDR | found | ratio |
| Neutrophil degranulation | 132/446 | 0.056 | 1.11e-16 | 5.73e-14 | 10/10 | 0.002 |
| Innate immune system | 185/775 | 0.097 | 1.11e-16 | 5.73e-14 | 126/347 | 0.055 |
| Platelet degranulation | 36/102 | 0.013 | 2.47e-08 | 8.50e-06 | 6/8 | 0.001 |
| AUF1 (hnRNP D0) binds and destabilizes mRNA | 23/47 | 0.006 | 3.31e-08 | 8.54e-06 | 2/3 | 0.0005 |
| Response to elevated platelet cytosolic Ca2+ | 36/106 | 0.013 | 6.28e-08 | 1.29e-05 | 6/11 | 0.002 |
| Dectin-1 mediated noncanonical NF-kB signaling | 23/51 | 0.006 | 1.38e-07 | 1.93e-05 | 3/8 | 0.001 |
| NIK→noncanonical NF-kB signaling | 23/51 | 0.006 | 1.38e-07 | 1.93e-05 | 3/9 | 0.001 |
