# Supplementary material for: Pulmonary and systemic responses to aerosolized lysate of Staphylococcus aureus and Escherichia coli in calves
Source: BMC Vet Res. 2020 May 29;16:168. doi: 10.1186/s12917-020-02383-7 (PMC7260748; doi:10.1186/s12917-020-02383-7)

Additional File 9. Gross lung lesions at 24 hours after aerosol administration of *Staphylococcus aureus* and *Escherichia coli* lysate. **(A)** Calf 1 received 10^8^ cfu-equivalents and had no gross lesions in the lungs. **(B-D)** Calf 4; 10^11^ cfu-equivalents**;** multifocal areas of consolidation (arrow), interlobular edema and small red foci within the lung tissue. (**E-F)** Calf 5; 10^12^ cfu-equivalents*;* mild edema with red mottling and rare small red-purple foci.


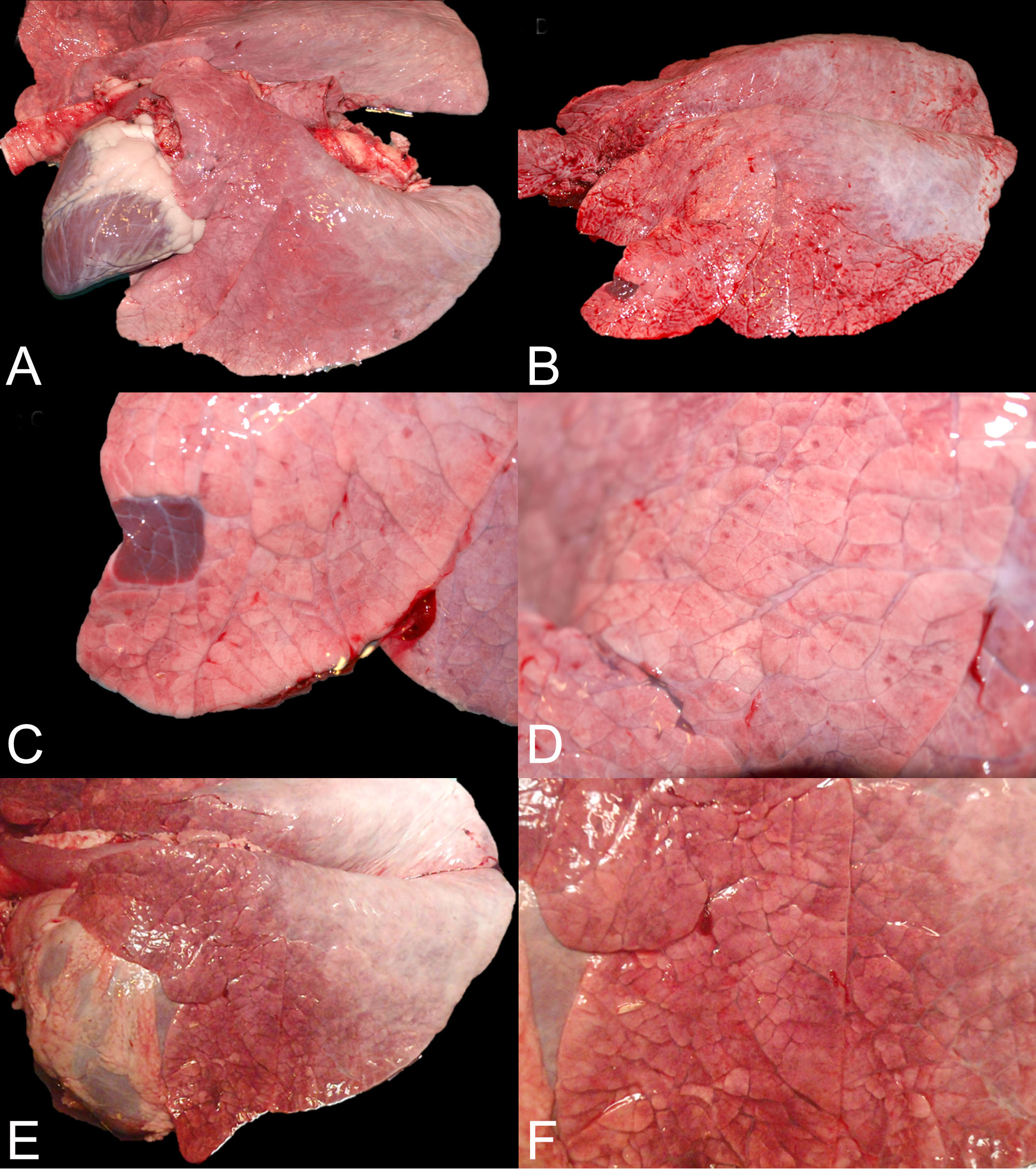

Supplement: Supplementary file 9 — Additional file 9. Gross lung lesions at 24 h after aerosol administration of Staphylococcus aureus and Escherichia coli lysate. [file 12917_2020_2383_MOESM9_ESM.docx]
