# Supplementary material for: Pulmonary and systemic responses to aerosolized lysate of Staphylococcus aureus and Escherichia coli in calves
Source: BMC Vet Res. 2020 May 29;16:168. doi: 10.1186/s12917-020-02383-7 (PMC7260748; doi:10.1186/s12917-020-02383-7)
Supplement: Supplementary file 10 — Additional file 10. Histologic lesions in respiratory tissues from 4 calves treated with aerosolized Staphylococcus aureus and Escherichia coli lysate. [file 12917_2020_2383_MOESM10_ESM.docx]

Additional File 10. Histologic lesions in respiratory tissues from 4 calves treated with aerosolized *Staphylococcus aureus* and *Escherichia coli* lysate at 10^8^ (A1-4), 10^9^ (B1-4), 10^10^ (C1-4), and 10^11^ (D1-4) colony forming unit equivalents. All sections of upper (thoracic) trachea (A1-D1) and lower (cervical) trachea (A2-D2) have small numbers of neutrophils within the lamina epithelium and submucosa, and variable numbers of lymphocytes and plasma cells. Bronchioles (A3-D3) have neutrophils within the submucosa, epithelium and lumens. Alveoli (A4-D4) contain increasing numbers of neutrophils with higher doses of lysate.

**­**

**
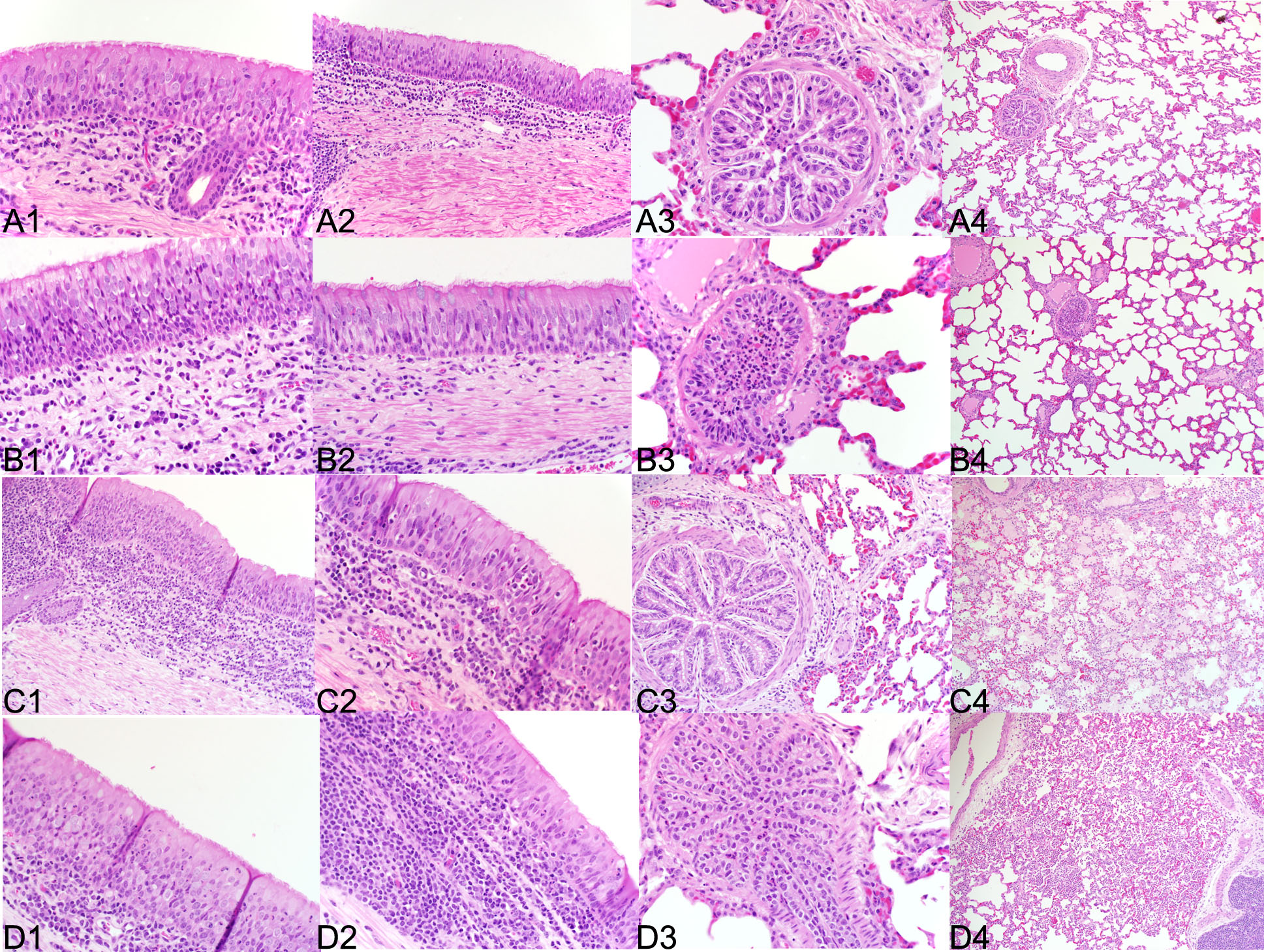
**
