# Supplementary material for: Pulmonary and systemic responses to aerosolized lysate of Staphylococcus aureus and Escherichia coli in calves
Source: BMC Vet Res. 2020 May 29;16:168. doi: 10.1186/s12917-020-02383-7 (PMC7260748; doi:10.1186/s12917-020-02383-7)
Supplement: Supplementary file 11 — Additional file 11. Clinical scoring system. [file 12917_2020_2383_MOESM11_ESM.docx]

Additional File 11. Clinical scoring system. Adapted from Potter et al., 1999.

| **Feature** | **Score Description** | |
| --- | --- | --- |
| **Demeanor** | 0 | Not depressed. |
|  | 1 | Rarely stands alone, ears droop slightly, moves away when people approach. |
|  | 2 | Walks slowly, lethargic, sometimes stands with head low, easy to corner but difficult to catch. |
|  | 3 | Uninterested in environment, little response when people enter pen, easy to catch, lies in sternal recumbency frequently. |
|  | 4 | Lies down most of the time, stands only occasionally, doesn’t respond when people enter pen. |
| **Appetite**  **(only assessed at feedings)** | 0 | Animal is seen eating with normal vigour. |
|  | 1 | Nibbles food but little is consumed. |
|  | 2 | Doesn’t eat. |
| **Strength** | 0 | Normal and difficult to catch. |
|  | 1 | Walks slowly, mildly unsteady gait, easier to catch than normal. |
|  | 2 | Staggers or knuckles occasionally, recumbent less than 50% of the time, becomes recumbent from standing when people are in the pen, obviously unsteady gait. |
|  | 3 | Recumbent most of the time but will rise when stimulated. |
|  | 4 | Recumbent and will not rise when stimulated. |
| **Effort of breathing** | 0 | Normal breathing pattern. Mild but detectable increase in respiratory effort when stressed. |
|  | 1 | Obvious increase in respiratory effort when stressed, subtle increase when not stressed. |
|  | 2 | Obvious increase in respiratory effort when not stressed (observed from a distance). |
|  | 3 | Open mouth breathing or marked increase in respiratory effort. |
