# Supplementary material for: Pulmonary and systemic responses to aerosolized lysate of Staphylococcus aureus and Escherichia coli in calves
Source: BMC Vet Res. 2020 May 29;16:168. doi: 10.1186/s12917-020-02383-7 (PMC7260748; doi:10.1186/s12917-020-02383-7)
Supplement: Supplementary file 12 — Additional File 12. Additional methods for cytokine analysis and mass spectrometry. [file 12917_2020_2383_MOESM12_ESM.docx]

Additional File 12. Additional methods for cytokine analysis and mass spectrometry.

A. Quantification of cytokines.

Cytokines were quantified using the U-plex assay platform (MSD, Rockville, MD) assembled according to manufacturer’s instructions using a chemiluminescent readout. In brief, 0.5 mL of all biotinylated antibodies were diluted to 10 µg/mL with Diluent 100, and paired to their respective linkers by adding 750 mL of supplied Linker solution and incubated for 30 minutes followed by addition of Stop solution for another 30 minutes. The linked antibodies for panel 1 were then diluted 10-fold into Stop solution. The panel 2 linked antibodies were diluted 20-fold (IL-10 and IL-17A) or 40-fold (IL-8 and IFN-γ) into Stop solution. Plates were coated by adding 50 µL of antibody solutions per well and were incubated covered with shaking for 1 hour at room temperature. Plates were washed 3 times with PBS/Tween20 prior to samples or standards being added at 30 mL per well and incubated as above. Plates were washed 3 times before detection antibodies were added along with 2% rabbit serum to block and incubated as above. Plates were washed 3 times before addition of 150 µL 2X Read buffer and Results were read immediately on the SI6000 instrument (MSD, Rockville, MD) and were reported as pg/mL. For soluble protein levels, a BCA protein kit (Thermofisher, Rockford, IL) was used. The cytokine concentrations were normalized to the total protein levels for each sample.

B. Methods for mass spectrometric analysis of bronchoalveolar lavage fluid.

Lyophilized peptide mixtures were dissolved in 0.1% formic acid and loaded onto a 75 μm x 50 cm PepMax RSLC EASY-Spray column filled with 2 μM C18 beads (ThermoFisher San, Jose CA) at a pressure of 900 Bar and a temperature of 60C. Peptides were eluted over 180 minutes at a rate of 250 nL/min using a gradient set up as described in supplementar S2.XX.follows, where Buffer A is 0.1% Formic acid and Buffer B is 80% Acetonitrie, 0.1% Formic Acid, all v/v in HPLC grade water.

| Time | Duration | % B |
| --- | --- | --- |
| 0 | 0 | 0 |
| 168 | 168 | 42 |
| 170 | 2 | 100 |
| 180 | 10 | 100 |

Peptides were introduced by nano-electrospray into the Fusion- Lumos mass spectrometer (Thermo-Fisher). Data were acquired using the MultiNotch MS3 acquisition with synchronous precursor selection (SPS) with a cycle time of 5 seconds. MS1 acquisition was performed with a scan range of 550m/z - 1800 m/z with resolution set to 120 000, maximum injection time of 50ms and AGC target set to 4e5. Isolation for MS2 scans was performed in the quadrupole, with an isolation window of 0.7. MS2 scans were done in the linear ion trap with a maximum injection time of 50ms and a normalized collision energy of 35%. For MS3 scans, HCD was used, with a collision energy of 30% and scans were measured in the orbitrap with a resolution of 50000, a scan range of 100m/z-500m/z, an AGC Target of 3e4, and a maximum injection time of 50ms. The dynamic exclusion was applied using a maximum exclusion list of 500 with one an exclusion duration of 20 s.
